# A Knowledge-Based Molecular Single-Source Precursor Approach to Nickel Chalcogenide Precatalysts for Electrocatalytic Water, Alcohol, and Aldehyde Oxidations

**DOI:** 10.1021/acsnano.4c08058

**Published:** 2024-12-03

**Authors:** Basundhara Dasgupta, Shenglai Yao, Indranil Mondal, Stefan Mebs, Johannes Schmidt, Konstantin Laun, Ingo Zebger, Holger Dau, Matthias Driess, Prashanth W. Menezes

**Affiliations:** § Department of Chemistry: Metalorganics and Inorganic Materials, Technische Universität Berlin, Straße des 17. Juni 115, Sekr. C2, Berlin 10623, Germany; † School of Chemistry, Indian Institute of Science Education and Research, Thiruvananthapuram, Kerala 695551, India; ‡ Department of Physics, Freie Universität Berlin, Arnimallee 14, Berlin 14195, Germany; £ Department of Chemistry: Functional Materials, Technische Universität Berlin, Hardenbergstraße 40, Berlin 10623, Germany; ∥ Department of Chemistry: Physical Chemistry/Biophysical Chemistry, Technische Universität Berlin, Straße des 17 Juni 135, Sekr. PC14, Berlin 10623, Germany; ¶ Materials Chemistry Group for Thin Film Catalysis − CatLab, Helmholtz-Zentrum Berlin für Materialien und Energie, Albert-Einstein-Straße 15, Berlin 12489, Germany

**Keywords:** nickel chalcogenides, oxygen evolution reaction, organic oxidation reaction, nickel oxyhydroxide, single-source precursor

## Abstract

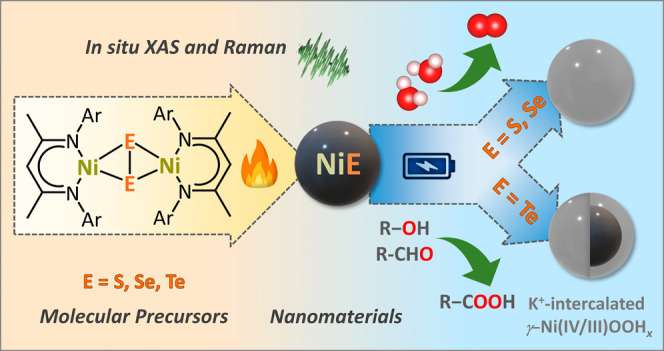

The development and comprehensive understanding of nickel chalcogenides are critical since they constitute a class of efficient electro(pre)catalysts for the oxygen evolution reaction (OER) and value-added organic oxidations. This study introduces a knowledge-based facile approach to analogous NiE (E = S, Se, Te) phases, originating from molecular β-diketiminato [Ni_2_E_2_] complexes and their application for OER and organic oxidations. The recorded activity trends for both target reactions follow the order NiSe > NiS > NiTe. Notably, NiSe displayed efficient performance for both OER and the selective oxidation of benzyl alcohol and 5-hydroxymethylfurfural, exhibiting stability in OER for 11 days under industrially pertinent conditions. Comprehensive analysis, including quasi *in situ* X-ray absorption and Raman spectroscopy, in combination with several *ex situ* techniques, revealed a material reconstruction process under alkaline OER conditions, involving chalcogen leaching. While NiS and NiSe experienced full chalcogen leaching and reconstruction into Ni^III/IV^ oxyhydroxide active phases with intercalated potassium ions, the transformation of NiTe is incomplete. This study highlights the structure–activity relationship of a whole series of analogous nickel chalcogenides, directly linking material activity to the availability of active sites for catalysis. Such findings hold great promise for the development of efficient electrocatalysts for a wide range of applications, impacting various industrial processes and sustainable energy solutions.

## Introduction

Electrolysis of water for green hydrogen fuel production has emerged as a compelling carbon-neutral approach to address the world’s growing energy requirements.^1,2^ However, the large-scale implementation of water-splitting is hindered by the anodic oxygen evolution reaction (OER), a process entailing four-electron transfer and numerous high-energy reaction intermediates.^3−6^ Partly, organic oxidation reactions can effectively supersede the sluggish OER (hybrid-water electrolysis), and accelerate the overall hydrogen production rate.^7−9^ This also significantly enhances the techno-economic viability of water-splitting as selective valued-added organic oxidation products are formed at the anode, as opposed to the less valuable oxygen. In this regard, Ni chalcogenide-based materials have evolved as prudent choices for developing electrooxidation catalysts -due to their low-cost, earth-abundant, and non-toxic nature, and the high catalytic activity and stability of their active phases, which have been consistently observed under varied electrolyte environments.^10−16^

Interestingly, the significantly lower oxidation potentials of Ni and chalcogens compared to water causes them to undergo oxidation under anodic potential, forming Ni oxyhydroxides (NiO_*x*_H_*y*_) and water-soluble oxyanions (XO_3_^2–^, XO_4_^2–,^ X = S, Se, Te, *etc*.).^11,12,17−19^ Therefore, Ni chalcogenides typically function as precatalysts and undergo reconstruction via chalcogen leaching under oxidation potentials to form catalytically active NiO_*x*_H_*y*_ phases. The extent of chalcogen leaching has been found to depend on the nature of chalcogen, with sulfides and selenides tending to leach more readily, while tellurides exhibit more resistance.^12^ For example, Mabayoje et al. and Xu et al. showed that S and Se leach out completely from NiS and NiSe precatalysts, respectively, during OER to form NiO_*x*_H_*y*_ phases.^20,21^ However, Wang et al. found that NiTe_2_ undergoes partial transformation to NiO_*x*_H_*y*_ during OER.^22^ Nevertheless, in all the cases, the leaching of chalcogens has consistently resulted in the formation of NiO_*x*_H_*y*_ active phases with large electrochemically active surface areas (ECSA) and enriched with active sites, edge-sites, vacancies, and defects- thereby leading to superior activities in contrast to NiO_*x*_H_*y*_ phases formed without sacrificial chalcogens.^11,12,17−22^ Interestingly, the leached chalcogenate oxyanions have also been observed to adsorb on the surface of NiO_*x*_H_*y*_ active catalysts during both OER^11,23^ and organic oxidations (for example, amine oxidation^24^), and act as a secondary binding site to stabilize the reaction intermediates and boost the overall catalytic performance. Furthermore, reports have also shown that the presence of chalcogens facilitates access to the higher oxidation state Ni^IV^ during electrooxidations, for example, through either Se leaching-induced effects in (Ni_*x*_Co_1*–x*_)Se_2_^25^ and (Fe,Ni)Se_2_,^26^ or through S doping effects in Ni/Fe-coordination polymers.^27^ The existence of a high oxidation state of the Ni center has consistently proven to enhance catalytic performance by stabilizing and promoting the formation of reaction intermediates.^28−30^

In this regard, understanding the behavior of different Ni chalcogenide precatalysts during OER and organic oxidation, and the impact of the leaching of chalcogens on the structure and performance of the active catalyst is crucial. Several studies have compared the activity and structural reconstruction of different chalcogenides; for example, hydrothermally prepared NiS_2_, NiSe_2_ and NiTe precatalysts tested for OER provided insights into the role of different chalcogenate oxyanions in influencing the activity.^31^ Similarly, comparing NiS_*x*_ and NiSe_*x*_,^32^ as well as Ni_3_Se_2_ and Ni_3_Te_2_^33^ revealed how varying degrees of chalcogen leaching affect the OER activity. In the case of ethanol electrooxidation, Se-modification was observed to enhance the activity of a NiS_*x*_ catalyst, compared to unmodified NiS_*x*_ and NiSe_*x*_ catalysts.^34^ However, a comprehensive study comparing and interconnecting the catalytic performance with the active structural features for a series of structurally and compositionally similar Ni chalcogenide (S, Se, Te, *etc.*) precatalysts is notably lacking. The availability of the full series of analogous Ni chalcogenide precatalysts would clarify the specific role of chalcogens in influencing the properties of the *in situ* derived NiO_*x*_H_*y*_ active catalyst while keeping other crucial variables like Ni-to-chalcogen ratio, particle size and shape, morphology, *etc*. constant. The absence of such studies likely stems from the lack of suitable precursors that can yield analogous Ni chalcogenides under identical preparation conditions.

To address the above challenge, the single-source precursor (SSP) method can be employed. By leveraging previous knowledge, SSPs can be strategically designed to incorporate all the defined chalcogens and therefore, their decomposition under identical conditions would readily yield the desired Ni chalcogenide phases, with similar properties. This method also offers advantages over conventional solid-state techniques by operating at lower temperatures and requiring fewer reaction times.^35−38^ Moreover, it predominantly produces pure phases with nanosized particles and a homogeneous elemental composition, as opposed to the high-temperature methods which mainly produce large particle sizes and often lead to mixed phases. Such an SSP approach has previously been used to isolate several challenging phases, including metal tellurides, intermetallics, metal-rich phases, heterostructures, etc., which are typically acquired through high-temperature solid-state methods.^35,39−43^ Consequently, there has been a surge of interest and expansion in the realm of materials derived from SSPs for electrocatalytic applications.

Given the aforementioned points, the current work aims to address the following research questions: (1) Can we strategically devise SSPs to enable the synthesis of analogous nickel chalcogenide (S, Se, and Te) phases that are otherwise difficult to achieve through alternative energy-consuming synthetic methods? (2) Do these materials demonstrate good OER activity and durability under both ambient and industrial conditions, and what is the observed activity trend for these materials? (3) What reconstructions and active structural features do these precatalysts exhibit under OER conditions? (4) How can we provide a reasonable explanation for any variations in activity among these materials, and do the chalcogens play a role during catalysis? And lastly, (5) Can these materials also be harnessed for the selective value-added oxidation of organic substrates?

**Scheme 1:**
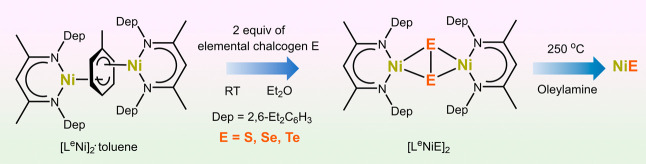
Synthesis of the Molecular **[L**^**e**^**NiE]**_**2**_ SSPs from the Toluene-Masked Ni^I^ Complex **[L**^**e**^**Ni]**_**2**_**·Toluene** and Their Subsequent Decomposition via Hot Injection to Produce Nanostructured Ni Chalcogenides (NiE)

In this work, we introduce a class of **[L**^**e**^**NiE]**_**2**_ (E = S, Se, Te; L^e^ = HC(CMeNC_6_H_3_Et_2_)_2_]) SSPs, prepared by reaction of the toluene-masked β-diketiminato nickel(I) complex with elemental chalcogens. The SSPs were subjected to solvothermolysis, affording the desired crystalline and nanostructured analogous NiE phases (Scheme 1). The as-prepared materials exhibit promising OER performances and follow an activity trend of NiSe > NiS > NiTe. Notably, NiSe demonstrated high OER activity, requiring an overpotential of 247 ± 2 mV to reach the benchmark current density of 10 mA/cm^2^. Additionally, it displayed a Faradaic efficiency (FE) of ≈97%, and under industrially relevant conditions (500 mA/cm^2^, 65 °C and 6 M KOH), showed durability for 11 days. A range of quasi *in situ* and *ex situ* techniques unveiled that, under oxidation potentials, the chalcogens undergo leaching, resulting in complete reconstructions of NiS and NiSe, while NiTe experiences partial reconstruction, forming γ-Ni^IV/III^OOH_*x*_ active phases, with intercalated potassium ions. The variations in activity among these materials primarily arises from the differences in the availability of active sites for catalysis. Furthermore, we employed these materials for the value-added oxidation of benzyl alcohol (BA) and observed a similar activity trend as determined for the OER. The NiSe precatalyst was also applied for the oxidation of 5-hydroxymethylfurfural (HMF), demonstrating current densities surpassing 600 mA/cm^2^ and FEs of ≈99–100%, for both HMF and BA oxidations.

## Results and Discussion

### Synthesis and Characterization of the **[L^e^NiE]_2_** SSPs

Monovalent nickel complexes represent a convenient class of moderately strong one-electron reducing agents that have been successfully employed for small molecule activation.^44,45^ In fact, the toluene-masked β-diketiminato nickel(I) complex [L^D^Ni]_2_·toluene (L^D^ = HC(CMeNC_6_H_3_*i*Pr_2_)_2_])^46^ engenders the formation of the corresponding superoxonickel(II) complex through dioxygen activation,^47^ whereas its reaction with elemental chalcogens E (E = S, Se, Te) affords the [L^D^NiE]_2_ complexes^48,49^ with a butterfly like [Ni_2_E_2_] core. Building upon this foundation, we have successfully designed isostructural **[L**^**e**^**NiE]**_**2**_ complexes in this work, with the aim that these {Ni_2_E_2_} core complexes will serve as molecular SSPs for the facile synthesis of the whole series of Ni chalcogenides under similar synthetic conditions.

**Figure 1:**
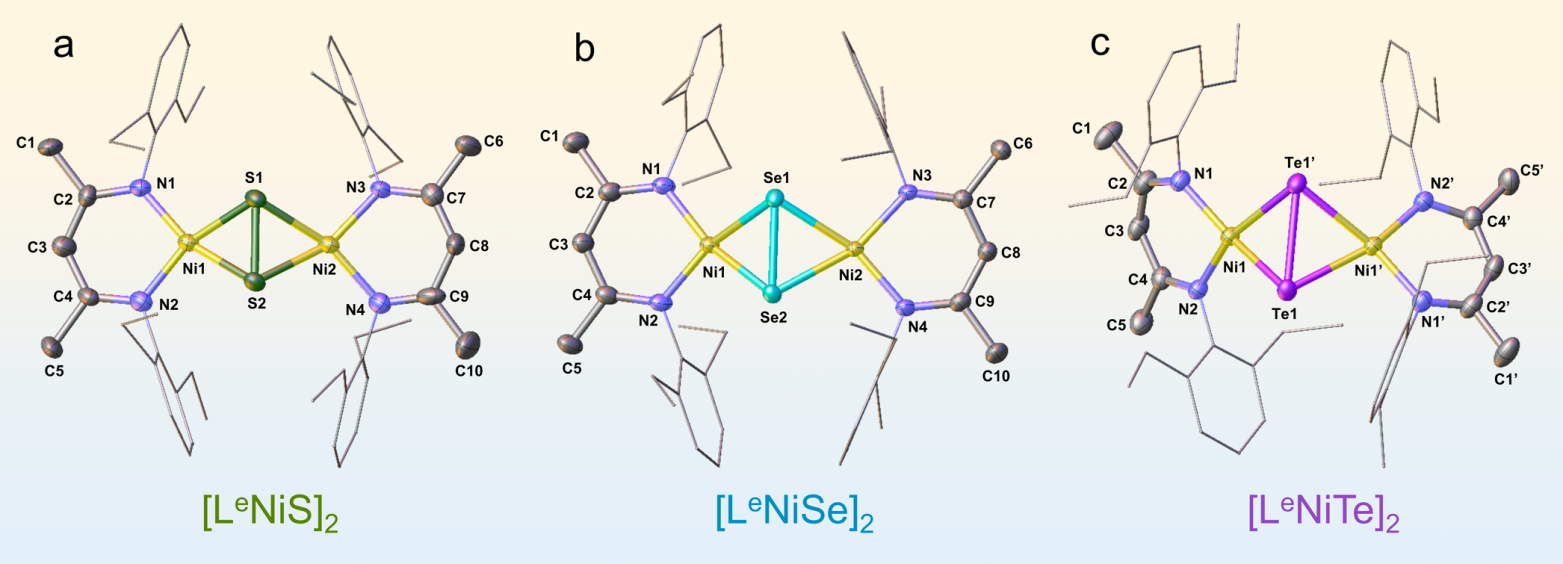
Molecular structures of the SSPs (a) **[L**^**e**^**NiS]**_**2**_, (b) **[L**^**e**^**NiSe]**_**2**_, and (c) **[L**^**e**^**NiTe]**_**2**_. Thermal ellipsoids are drawn at a 50% probability level. H atoms are omitted for clarity. Symmetry transformations are used to generate equivalent atoms with “′”: −*x* + 1/2, *y*, −z + 1/2.

The treatment of the toluene-masked Ni^I^ complex **[L**^**e**^**Ni]**_**2**_**·toluene** with 0.25 mol equiv of elemental sulfur (S_8_) and red selenium (Se_8_) in diethyl ether at room temperature promptly yielded **[L**^**e**^**NiS]**_**2**_ and **[L**^**e**^**NiSe]**_**2**_, respectively (Scheme 1). Due to the moderate reactivity of elemental tellurium, its reaction with **[L**^**e**^**Ni]**_**2**_**·toluene** required prolonged reaction time (3 days) but ultimately formed the desired tellurium congener, **[L**^**e**^**NiTe]**_**2**_. The synthesized complexes were unambiguously characterized, revealing their diamagnetic nature and the presence of two chemically equivalent β-diketiminato ligands (Figures S1–S6). Single-crystal X-ray diffraction (XRD) analyses revealed a butterfly like structure with a puckered Ni_2_E_2_ core for all three complexes, with two slightly distorted square-planar, tetracoordinate Ni^II^ atoms (Figure 1, Figures S7–S9, Tables S1–S6). As expected, the E–E distances are very close to the corresponding distances observed in the [L^D^NiE]_2_ complexes.^48,49^

### Synthesis and Characterization of Nanostructured Ni Chalcogenides

**Figure 2:**
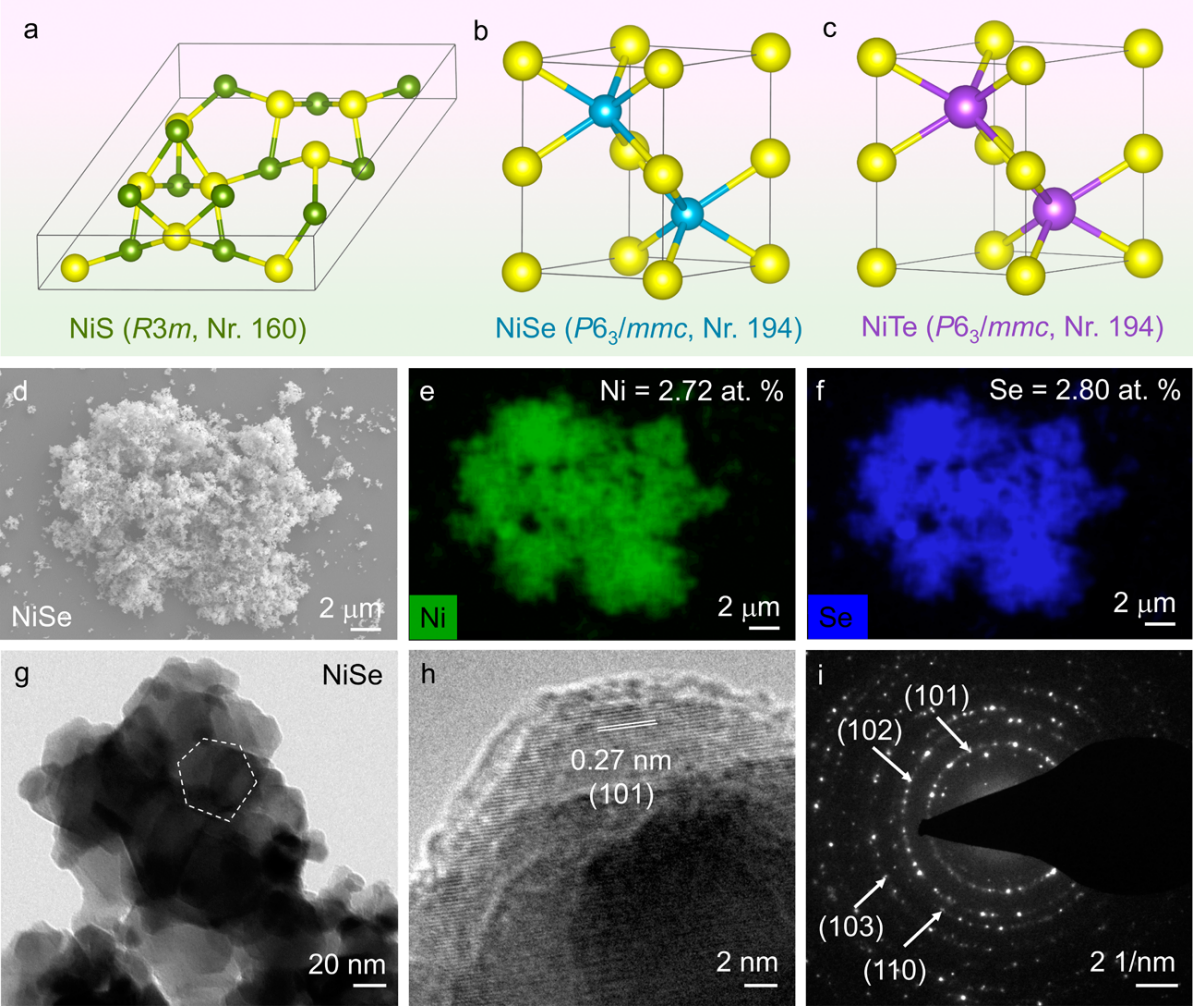
Crystal structures of (a) NiS, (b) NiSe, and (c) NiTe, illustrating Ni (yellow) and S (green)/Se (blue)/Te (purple) atoms within their respective unit cells (gray lines). (d) SEM image and elemental mapping showing a homogeneous distribution of (e) Ni (green) and (f) Se (blue) in NiSe. (g) TEM image of NiSe displaying irregular hexagon-shaped particles, (h) HR-TEM displaying a lattice fringe spacing of 0.27 nm corresponding to the (101) plane of NiSe, and (i) SAED exhibiting a ring pattern typical for polycrystalline materials, where the (101), (102), (110), and (103) planes can be identified (JCPDS 2-892). The SEM and TEM characterizations of NiS and NiTe are provided in Figures S14 and S16–S18 of the Supporting Information.

The SSPs **[L**^**e**^**NiE]**_**2**_ were subjected to hot-injection at 250 °C in oleylamine to yield the Ni chalcogenide nanomaterials (Scheme 1). Phase-pure powder X-ray diffraction (PXRD) patterns were acquired for the materials, which showed that NiS crystallizes in the rhombohedral space group *R*3*m* (Nr.160), while NiSe and NiTe both crystallize in the hexagonal space group *P*6_3_/*mmc* (Nr. 194) (Figure 2a–c and Figures S10–S13). Scanning electron microscopy (SEM) images and elemental mapping of the materials displayed agglomerated nanostructured particles, with homogeneously distributed Ni and chalcogens (Figure 2d–f and Figures S14–16). Moreover, inductively coupled plasma optical emission spectroscopy (ICP-OES) and energy dispersive X-ray (EDX) quantification confirmed ≈1:1 ratio of Ni and chalcogens in the materials (Figures S14–16, Table S7). Investigation of the atomic structure of the materials was conducted using transmission electron microscopy (TEM), which showed the presence of distorted hexagon-shaped particles with dimensions ranging from 30 to 50 nm (Figure 2g, Figures S17 and S18). Furthermore, high-resolution TEM (HR-TEM) exhibited lattice fringes, while selected area electron diffraction (SAED) displayed diffraction rings corresponding to the crystal structures of the materials, aligning with the data obtained from the PXRD patterns (Figure 2h,i, Figures S17 and S18). Moreover, the as-synthesized chalcogenides exhibited a distinct polycrystalline nature, as substantiated by the scattered ring patterns observed in SAED.

Consequently, the knowledge-based low-temperature SSP approach readily yielded a series of Ni chalcogenides that have similar composition, morphology, and particle shape and sizes. This offers a compelling foundation for precisely comparing their activities and behaviors under electrocatalytic conditions, offering valuable insights into the complete series of chalcogenides (NiS, NiSe and NiTe).

### Electrochemical Performance for OER

To assess the OER performance of the materials, we applied the as-synthesized materials onto nickel foam (NF) and fluorine-doped tin oxide (FTO) substrates through electrophoretic deposition (EPD). This process yielded uniform films with a mass loading of 0.9 ± 0.1 mg/cm^2^ on NF and 0.5 ± 0.1 mg/cm^2^ on FTO. Subsequent characterization of the films confirmed that the identity of the materials was conserved during EPD (Figures S19 and S20, Table S7). The electrochemical OER measurements were first recorded on the conducting and porous NF substrate, in 1 M KOH, using a three-electrode setup (see details in the Supporting Information).

**Figure 3:**
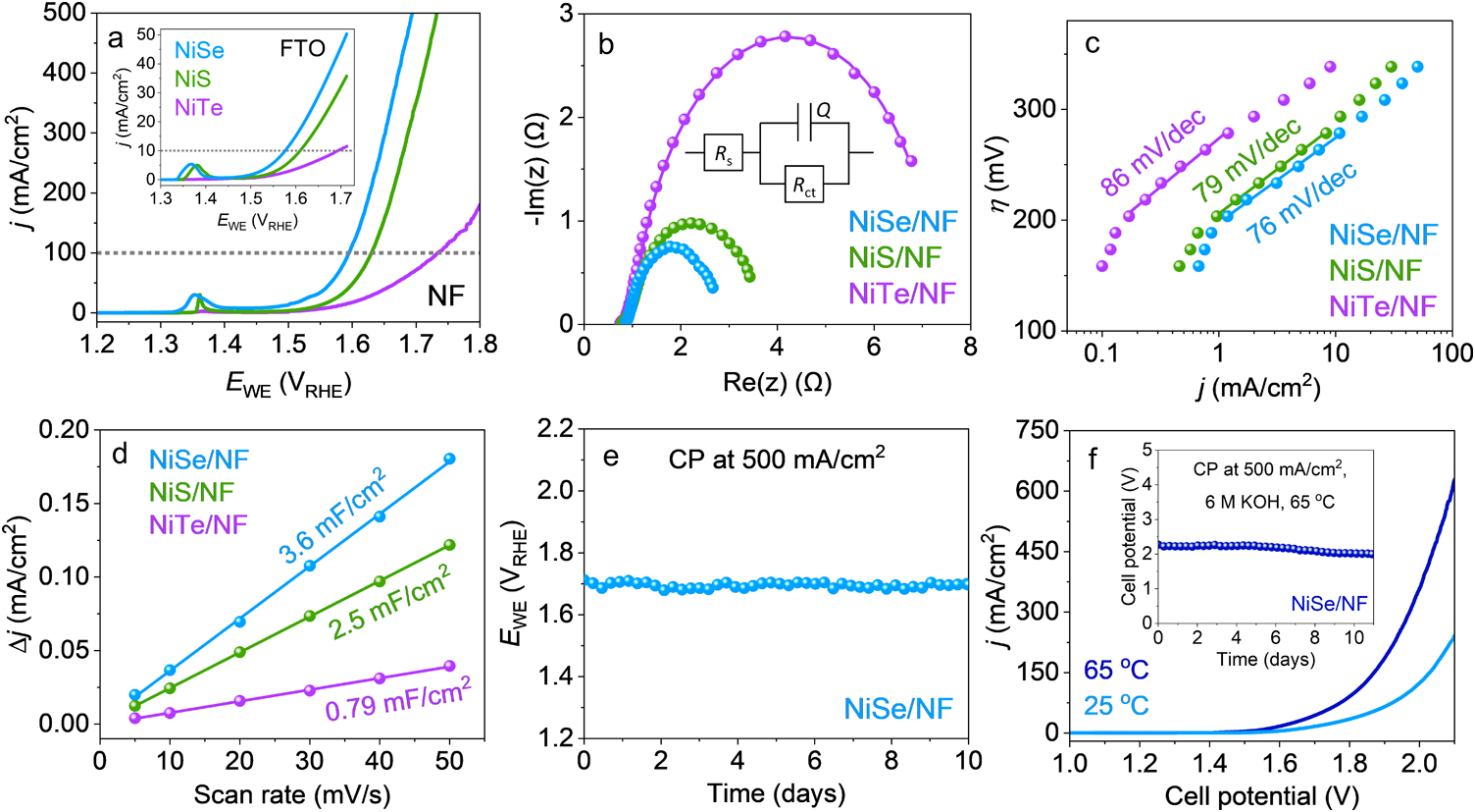
(a–e) Electrochemical measurements for OER recorded in a three-electrode setup, with 1 M KOH and at 25 °C. (a) LSV plots to determine the OER activities of NiE on NF at a scan speed of 1 mV/s and on FTO at a scan speed of 5 mV/s (inset). (b) Nyquist plots of NiE/NF measured at 1.56 *V*_RHE_ and fitted with an equivalent Randles circuit (inset). (c) Steady-state Tafel slopes of NiE/NF, measured by applying constant potentials with a step size of 15 mV. (d) *C*_dl_ values of NiE/NF after CP at 10 mA/cm^2^ for 24 h, determined from CVs recorded in a non-Faradaic potential range. (e) CP at 500 mA/cm^2^ for NiSe/NF showing a stable activity for 10 days. (f) LSVs (at 5 mV/s, without *iR* compensation) recorded for NiSe/NF(+)||Pt(−) at 25 and 65 °C in 6 M KOH, using a two-electrode setup. CP for overall water-splitting at 500 mA/cm^2^, 65 °C, in 6 M KOH with the NiSe/NF anode showing a stable activity for 11 days (inset).

Prior to commencing the measurements, the as-deposited materials were activated by performing continuous cyclic voltammetry (CV) cycles until a stable current response were obtained (10 cycles). Interestingly, the lowest overpotential (η) was obtained for NiSe [η at 10 mA/cm^2^ (η_10_) = 247 ± 2 mV], followed by NiS (η_10_ = 310 ± 3 mV), and then NiTe (η_10_ = 339 ± 5 mV) (Figure 3a). The observed overpotentials, particularly that of NiSe, are similar to the values reported for other Ni chalcogenide materials in previous studies (Table S8). Notably, all the examined materials showed a redox feature between 1.20 and 1.45 *V*_RHE_ corresponding to the Ni^II^ → Ni^III/IV^ oxidation (Figure S21), consistent with prior findings on Ni-based materials.^28-30^^,^^50-53^ Upon integration and subsequent charge calculation from the reduction peak, it was evident that NiSe displayed the highest amount of redox active Ni electrons compared to the other chalcogenides. Nyquist plots constructed from electrochemical impedance spectroscopy showed the smallest charge transfer resistance (*R*_ct_) for NiSe (Figure 3b, Table S9). Furthermore, turnover frequency (TOF), which is a reliable parameter to study the intrinsic activity of a catalyst,^54,55^ was observed to be similar for all three materials (Table S10). Similar Tafel slopes were obtained for all the materials, implying the presence of similar active sites, OER mechanism, and rate-determining step for all the presented Ni-based materials (Figure 3c).^56,57^ The ECSA was estimated by calculating the double-layer capacitance (*C*_dl_) values, which are directly proportional to ECSA,^58^ after exposing the samples to chronopotentiometry (CP) at 10 mA/cm^2^ for 24 h (Figure S22). The highest *C*_dl_ was attained for NiSe as compared to NiS and NiTe (please note that the *C*_dl_ measurements can be unprecise due to conductivity limitations^59−61^) (Figure 3d and Figure S23).

Furthermore, a OER FE of ≈97% was obtained for the best-performing material, i.e., NiSe, which was determined by measuring the evolved O_2_ gas (Table S11). To test the industrial importance of the current OER activity of NiSe, the NiSe/NF was first fully activated using CV cycling (10 cycles) and then tested for chronopotentiometry (CP) at a high current density of 500 mA/cm^2^.^62^ Interestingly, a stable performance was observed for 10 days (Figure 3e). Given the demanding operational conditions of industrial alkaline water electrolyzers, requiring high temperatures and harsh electrolyte environments,^62^ we further employed the CV-activated NiSe/NF as the anode in a two-electrode setup, with Pt wire as the cathode, and performed overall water-splitting at 65 °C in 6 M KOH, (see details in the Supporting Information). Notably, the LSV current density of the overall water-splitting process increased significantly with increasing temperature (from 25 to 65 °C) (Figure 3f), which could be attributed to the enhanced mass transport of the KOH electrolyte at higher temperatures. Importantly, even under such harsh electrolyte conditions, no significant potential drop at 500 mA/cm^2^ was observed for 11 days (Figure 3f, inset). These results demonstrate the substantial potential of NiSe/NF OER material to be used for practical water-splitting applications in industrial settings.

To eliminate any potential contributions of the NF substrate to the electrocatalytic activity, the LSVs of the materials were further measured on FTO. The observed activity trend was similar to that on NF, displaying η_10_ values of 340 ± 6 mV, 378 ± 8 mV, and 464 ± 11 mV for NiSe/FTO, NiS/FTO, and NiTe/FTO, respectively (Figure 3a inset, Table S8). Similarly, the *R*_ct_ values exhibited a consistent trend as obtained on NF (Figure S25, Table S12). Therefore, the overpotential trend from the geometric current densities of the materials aligns with the amount of Ni redox active electrons, *R*_ct_ and *C*_dl_ trend, while their intrinsic activities and type of Ni active sites, as determined from the TOFs and tafel slopes, are similar. Furthermore, the best-performing material, NiSe, demonstrated stable performance under industrially pertinent conditions.

### *Ex Situ* Post-OER Characterization

**Figure 4:**
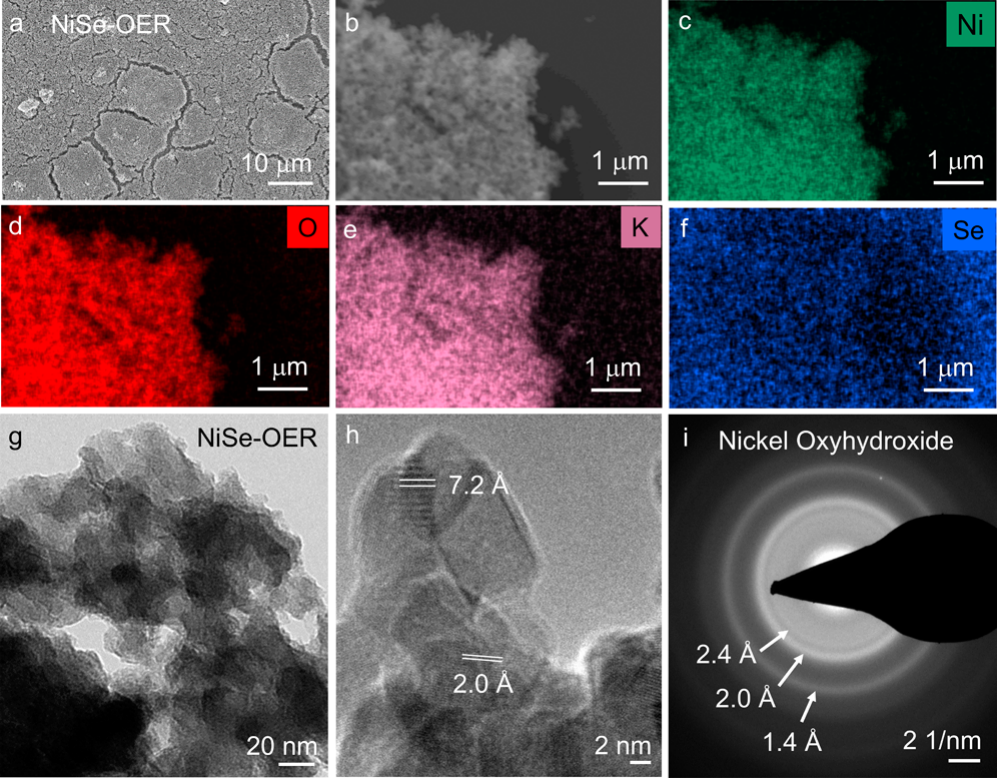
Characterization of NiSe after 24 h of CP at 10 mA/cm^2^ in 1 M KOH. (a) SEM image of the NiSe film on FTO reveals the appearance of cracks after OER treatment. (b) SEM image and elemental mapping of NiSe after (recorded by scratching off the film from FTO) shows a homogeneous distribution of (c) Ni (green), (d) O (red), (e) K (purple), and an absence of (f) Se (blue) in the sample. The post-OER (g) TEM image of NiSe shows a sheet-like structure, and the (h) HR-TEM image and the corresponding (i) SAED rings confirm the complete transformation of NiSe into a γ-NiOOH_*x*_ active phase (JCPDS 6-75), with a *d*-spacing of ≈7.2 Å. The post-OER SEM and TEM characterizations of NiS and NiTe are given in Figures S28, S30, S32, and S33 of the Supporting Information.

To gain a comprehensive insight into the structural transformations occurring within the materials during OER, we subjected them to a constant current density of 10 mA/cm^2^ for 24 h on FTO substrates (Figure S26) and subsequently, characterized them systematically. Interestingly, SEM analysis of the NiS and NiSe films after OER reveal that the surfaces of the films were substantially altered, whereas the NiTe film displayed no discernible change (Figure 4a and Figure S27). Elemental mapping, EDX, and ICP-OES, after both CV activation and OER CP, unveiled a complete leaching of S and Se, and only ≈35% loss of Te, from the respective materials (Figure 4b–f, Figures S28–S31, Table S7). This implies that the chalcogens dissolve as water-soluble (oxy)anionic species in the electrolyte.^63,64^ Additionally, elemental mapping showed a homogeneous distribution of Ni, K, and O throughout the samples, indicating the formation of an oxidic Ni–K phase originating from the Ni chalcogenide precatalysts during OER (Figure 4b–f, Figures S28 and S30). Moreover, TEM, HR-TEM and corresponding SAED analyses conducted on the post-OER samples uncovered a complete transformation of NiS and NiSe and a partial transformation of NiTe into nanocrystalline nickel oxyhydroxide (NiOOH) phases (Figure 4g–i, Figures S32 and S33). Notably, the *d*-spacing for the NiSe-derived layered NiOOH phase was obtained to be ≈7.2 Å. The observed *d*-spacing and the homogeneous distribution of Ni, K and O in NiSe after OER are consistent with the recently reported deprotonated γ-NiOOH_*x*_ phase, with intercalated potassium ions and water molecules.^65,66^ Similar conclusions regarding the formation of K^+^-intercalated γ-NiOOH_*x*_ active phases can be drawn for the NiS and NiTe precatalysts as well. Furthermore, no significant iron incorporation from the KOH electrolyte in the samples after OER was observed from EDX, which is crucial to note here since iron incorporation is known to enhance the activity of Ni-based materials (Figures S28–S30).^67^

To elucidate the changes in electronic features during OER, XPS measurements were performed. The Ni and chalcogen XPS spectra of the as-synthesized and as-deposited samples showed similar surface electronic structures, indicating that the properties of the materials did not alter after EPD (Figure S34). The deconvoluted Ni 2p XPS spectra of the Ni chalcogenides revealed the presence of Ni^II/III^ species at the surfaces of the catalysts, in contrast to the as-synthesized and as-deposited samples, indicating the transformation of the materials to the γ-NiOOH_*x*_ phases (Figures S34a and S35).^68,69^ In the S 2p and Se 3d XPS spectra, no peaks above noise level were detected after OER, due to the complete leaching of the chalcogens from NiS and NiSe, respectively (Figure S34b). However, the Te 3d spectrum displayed Te^IV^ peaks, which could be attributed to the remaining oxidic Te species present on the surface of NiTe (Figure S34b).^70^ The Te^2–^ species from the untransformed NiTe was not detected due to this oxidic Te species on the surface.

We further treated the materials to a constant current of 500 mA/cm^2^ on NF for 24 h to evaluate the stability and structural changes under industrially relevant high current densities (Figure S24). SEM images revealed significant surface alterations, with the formation of porous structures (Figure S36). Elemental mapping and SEM-EDX analysis showed a complete leaching of S and Se from NiS and NiSe, respectively, while a small amount of Te remained in NiTe (Figures S37–S39). Moreover, significant potassium incorporation and negligible iron incorporation from the KOH electrolyte were observed. TEM, HR-TEM and SAED analysis of NiSe confirmed a complete reconstruction into the nanocrystalline NiOOH phase, which is a partly deprotonated, K^+^-intercalated γ-NiOOH_*x*_, as reasoned previously (Figure S40). However, for NiTe, the γ-NiOOH_*x*_ was formed majorly, with some residual NiTe phase (Figure S41). TEM-EDX quantification revealed a Ni:Te ratio of 1:0.25, indicating that ≈75% of the Te had leached out. Therefore, the results from *ex situ* characterizations after 24 h CP at both 10 mA/cm^2^ and 500 mA/cm^2^ reveal that NiS and NiSe reconstruct completely while NiTe reconstructs partially into γ-NiOOH_*x,*_ with intercalated K^+^ ions. The facile leaching of S and Se, and the resistance of Te to oxidize and leach out, likely due to the increased metallic character of Te and hence the Ni–Te bond, is consistent with previous reports.^12,20−22^

### Quasi *In Situ* OER Characterization

**Figure 5:**
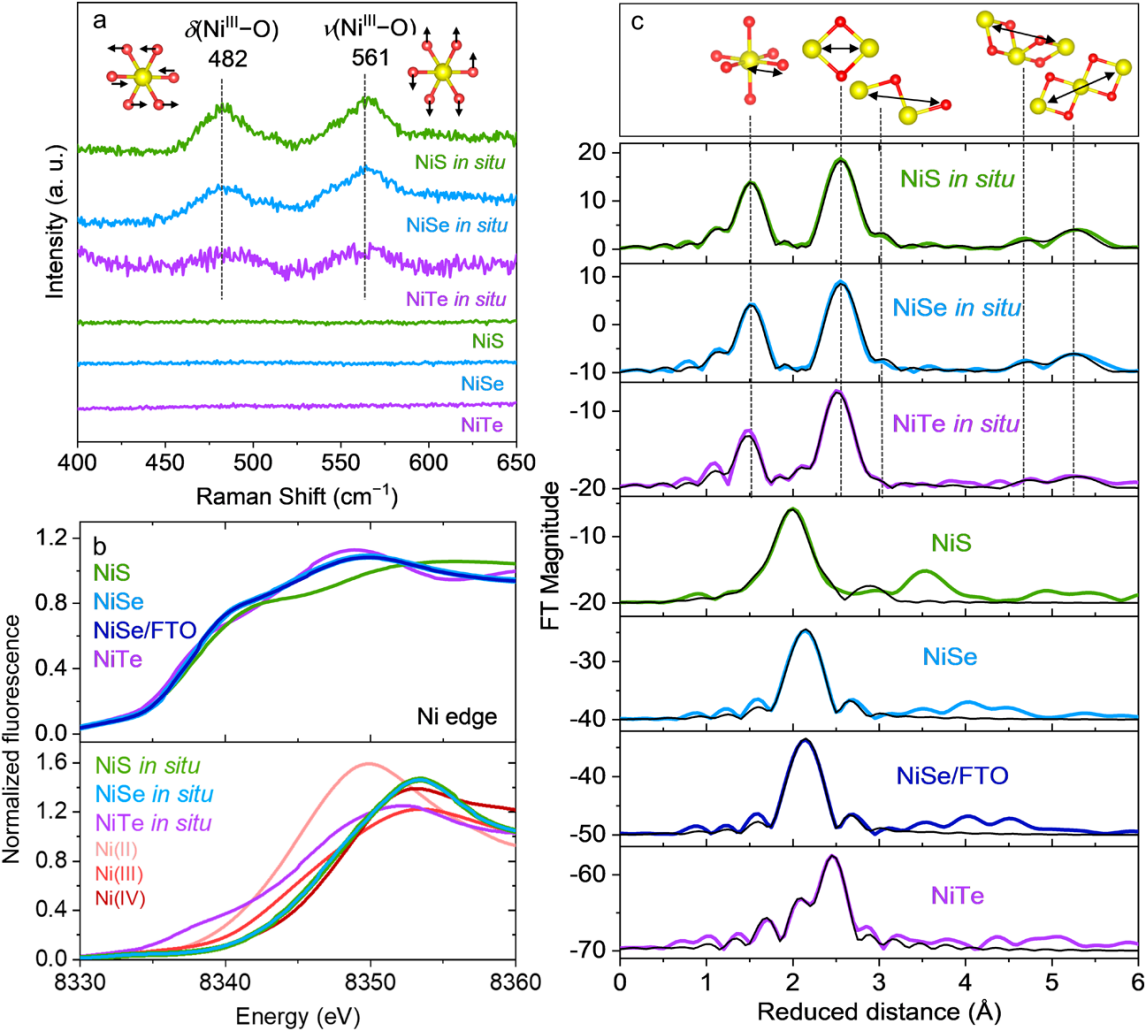
(a) Raman spectra of as-synthesized NiE powders and quasi *in situ* NiE samples freeze-quenched at 1.56 *V*_RHE_ after 24 h of CP at 10 mA/cm^2^. The bending [δ(Ni^III^–O)] and stretching [ν(Ni^III^–O)] vibrational modes of γ-NiOOH_*x*_ (Ni: yellow, O: red) were observed in the *in situ* samples. (b) Ni K-edge XANES spectra of the as-synthesized NiE powders, as-deposited NiSe on FTO, and quasi *in situ* NiE samples freeze-quenched at 1.56 *V*_RHE_ after 24 h of CP at 10 mA/cm^2^; the Ni^II^, Ni^III^, and Ni^IV^ spectra were collected for NiO, NiO_2_Li, and K_2_(Ni(H_2_IO_6_)_2_, respectively. (c) Fourier-transformed EXAFS data of the samples mentioned in panel (b), together with their simulations in black (see Tables S13–S19 for simulation parameters).

To acquire a deeper understanding of the dynamic active structure and oxidation states of the materials under operando conditions, quasi *in situ* Raman and X-ray absorption spectroscopy (XAS) were adopted. The *in situ* samples were prepared by freeze-quenching the samples in liquid N_2_ (at −196 °C) under applied chronoamperometric (CA) conditions (see the Supporting Information for more details). The *in situ* as well as *ex situ* OER Raman spectra of Ni chalcogenides exhibited distinctive peaks centered at 482 and 561 cm^–1^, corresponding to depolarized bending [δ(Ni^III^–O)] and polarized stretching [ν(Ni^III^–O)] vibrations of a γ-NiOOH_*x*_ phase (Figure 5a and Figure S42a).^71,72^ In contrast, for the as-synthesized Ni chalcogenides, no peaks evolved above the noise level. For the *in situ* samples, an additional band at ≈1070 cm^–1^ was observed (Figure S42b); in refs (71 and 72), a similar band has been assigned to an “active oxygen species”.

The Ni XANES spectra of the *in situ* NiS and NiSe samples displayed significant alterations in both shape and position, compared to the as-synthesized and as-deposited samples, strongly implicating a complete transformation of the materials into oxidic species (Figures 5b and S43). Interestingly, for the *in situ* samples, the increased K-edge energy indicated an average Ni oxidation state of ≈3.6, signifying the simultaneous presence of Ni^IV^ and Ni^III^ species during OER.^28–30,50–52^ Conversely, for NiTe, a much less pronounced shift in the Ni edge was observed in the *in situ* sample compared to the as-synthesized sample, which suggests a partial transformation of the material to a higher oxidation state active phase (Figure 5b and Figure S43). Furthermore, the *in situ* Se XANES spectrum of NiSe showed minimal intensity when compared to the as-synthesized and as-deposited NiSe, confirming a complete leaching of Se from the system, consistent with previous characterization results (Figure S44).

The Ni EXAFS spectra of as-synthesized NiS and NiSe powders were successfully simulated providing Ni–S/Se and Ni–Ni bond distances and coordination numbers that agree well with the crystallographic data (Figure 5c, Figure S45, Tables S13 and S14). Similarly, simulation of the EXAFS spectrum of as-deposited NiSe on FTO confirmed that the material remained unchanged after EPD (Table S15). For the as-synthesized NiTe powder, the Ni–Te and Ni–Ni distances could be effectively fitted only after considering a minor preoxidation Ni–O shell (Table S16). To derive deeper insights into the structural differences between the active phases of Ni chalcogenides for OER, *in situ* Ni EXAFS spectra were recorded and successfully simulated for five coordination shells of the expected NiOOH phase, formed by layers of edge-sharing [NiO_6_] octahedra (Figures 5c and 7b, Figure S45, Tables S17–S19).^59^ For all three samples, the first two closest shells, at distances of 1.87 and 2.83 Å, respectively, correspond to the Ni–O bond in the [NiO_6_] octahedron and the Ni–Ni bond between two edge-sharing [NiO_6_] octahedra.^73^ For *in situ* NiS (and NiSe), a population of 4.7 (and 4.8) and 4.7 (and 4.8) were observed for the two shells, respectively (Tables S17 and S18). Given that the theoretical population of both these shells is six, these results strongly indicate an essentially complete transformation of NiS and NiSe to the NiOOH phase during OER. As confirmed previously by characteristic Raman peaks at 482 and 561 cm^–1^ (Figure 5a and Figure S42), and the interlayer spacing of ≈7.2 Å in HR-TEM (Figure 4h), this NiOOH phase is identified as γ-NiOOH_*x*_.^65,71,72^ However, for *in situ* NiTe, a population of only 2.6 for the first Ni–O shell, and 1.9 for the second Ni–Ni shell was observed, along with the retention of the Ni–Te shell with a population of 1.0 (Table S19). These results indicate that NiTe transforms partially to the γ-NiOOH_*x*_ phase during OER. Similarly, the third Ni–O, fourth Ni–Ni and fifth Ni–Ni shells of the edge-sharing [NiO_6_] octahedra layer were also successfully fitted for the three *in situ* samples, with minimal variation in the distances, further confirming the formation of the γ-NiOOH_*x*_ active phase during OER (Figures 5c and 7b, Figure S45, Tables S17–S19).^59^ The populations of these shells were also the lowest for NiTe, compared to NiS and NiSe, both of which exhibited similar high populations. These results further confirm that NiTe undergoes partial while NiS and NiSe transform fully into the γ-NiOOH_*x*_ active phase. Therefore, the results from quasi *in situ* Raman and XAS measurements strongly support the findings from the post-OER *ex situ* characterizations. While Raman analyses confirmed the surface structure of the γ-NiOOH_*x*_ active phases by identifying the characteristic Ni^III^–O bonds, the XANES and EXAFS analyses revealed the different operando Ni oxidation states and local structures for the different Ni chalcogenide-derived active phases.

**Figure 6:**
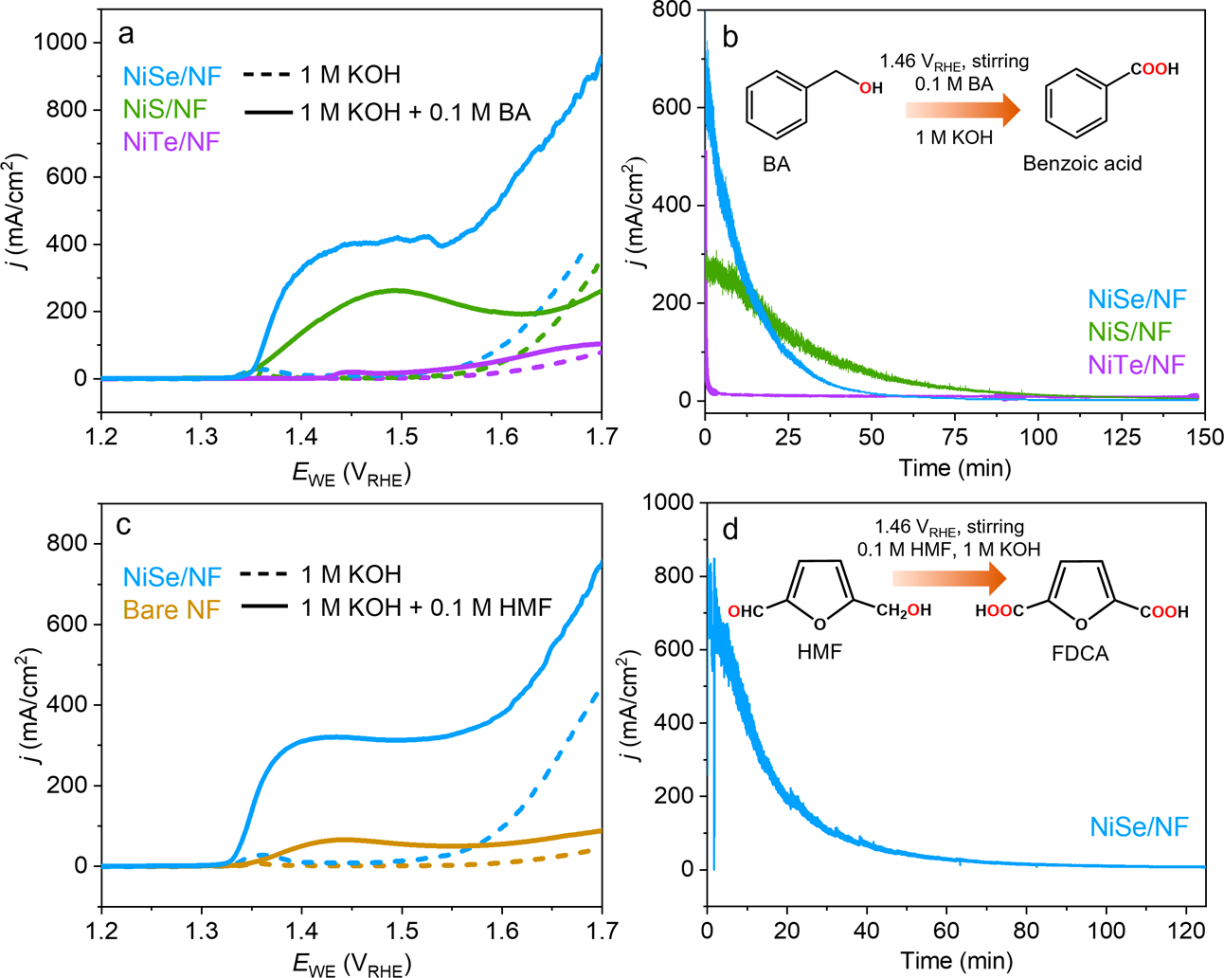
Value-added selective oxidation of BA and HMF performed in 1 M KOH at 25 °C. (a) LSV curves (at 5 mV/s) in the absence and presence of 0.1 M BA, without stirring, for NiE/NF. (b) Bulk CA electrolysis of 0.1 M BA, with stirring, at 1.46 *V*_RHE_ for NiE/NF, which was terminated after exactly passing the charge required for full conversion (578.9 C). (c) LSV curves (at 5 mV/s) of NiSe/NF in the absence and presence of 0.1 M HMF, without stirring. (d) Bulk CA electrolysis of 0.1 M HMF, with stirring, at 1.46 *V*_RHE_ for NiSe/NF, which was terminated after reaching the exact charge required for full conversion (868.4 C).

### Value-Added Selective Oxidation of BA and HMF

We further harnessed the potential of our NiE materials for the value-added oxidation of BA to benzoic acid, which finds significant applications in industry, with an annual production exceeding 640 kt.^74,75^ Traditionally, its production heavily depends on resource- and energy-demanding toluene oxidation. In pursuit of a greener synthetic approach, we substituted the OER with BA oxidation at the anode and coupled it with the hydrogen evolution reaction at the cathode. To achieve this, the Ni chalcogenide precatalysts were first activated through CV cycling on NF, in 1 M KOH, until stable current responses were obtained (10 cycles). Subsequently, LSVs were recorded with and without 0.1 M BA, under unstirred conditions (see details in the Supporting Information). Interestingly, for NiS and NiSe, the onset potential of BA oxidation (≈1.35 *V*_RHE_) preceded the onset of OER (≈1.47 *V*_RHE_) and displayed a substantial redox peak indicative of Ni^II^ → Ni^III/IV^ conversion (Figure 6a). This behavior is likely attributed to the reduction of partial Ni^III/IV^ species by BA to Ni^II^, which undergoes rapid oxidation and generates a larger current response.^76^ The BA oxidation activity trend was observed to be NiSe > NiS > NiTe, consistent with the OER activity trend. To quantitatively comprehend the product formations and FEs, the bulk oxidation of 0.1 M BA with NiE/NF electrodes were conducted by performing CA at 1.46 *V*_RHE_, with stirring (Figure 6b). Under these steady-state conditions, NiSe/NF achieved a high current density of over 600 mA/cm^2^. After 148 min, the exact charge required for full conversion (578.9 C) was passed for NiSe and ^1^H NMR confirmed the full conversion of BA to benzoic acid, thereby giving a FE of ≈100% (Figure S46). However, for NiS, and NiTe, after 148 min, the charge passed was 520.2 and 190.1 C, with NMR yields of ≈64% and ≈20%, respectively (Figure S47).

The high efficiency of NiSe in catalyzing the oxidation of BA encouraged us to explore its potential in the value-added oxidation of HMF. HMF holds significant importance as a biomass-derived platform chemical, and its electrocatalytic conversion to 2,5-furandicarboxylic acid (FDCA) can facilitate environmentally friendly polymer synthesis.^77^ FDCA serves as a vital precursor for the production of polyethylene-2,5-furandicarboxylate and poly(ethylene terephthalate) in the polymer industry.^77^ We recorded LSVs using the activated NiSe/NF electrode in the presence and absence of 0.1 M HMF, without stirring. Similar to BA oxidation, the introduction of HMF led to a substantial increase in the current density (Figure 6c). To further investigate, bulk electrolysis was performed with 0.1 M HMF at 1.46 *V*_RHE_ resulting in a current density exceeding 600 mA/cm^2^ (Figure 6d). Subsequent analysis of the reaction mixture using ^1^H NMR after the complete charge transfer (868.4 C in 125 min) indicated the selective formation of FDCA, with ≈99% yield; only a minor amount of formate as a degradation product was detected.^78^ Consequently, the obtained FE for HMF oxidation is ≈99% (Figure S48). As a control experiment, we performed HMF oxidation with bare NF and observed a very low current density both in the absence and presence of HMF (Figure 6c). The observed FE of BA and HMF oxidation with NiSe are comparable to those reported for other Ni-based catalysts in the literature (Tables S20 and S21). This emphasizes the potential of NiSe to be used for the selective value-added transformation of organic substrates.^79−82^

### Active Structures for OER and Variations in Catalytic Performances

**Figure 7:**
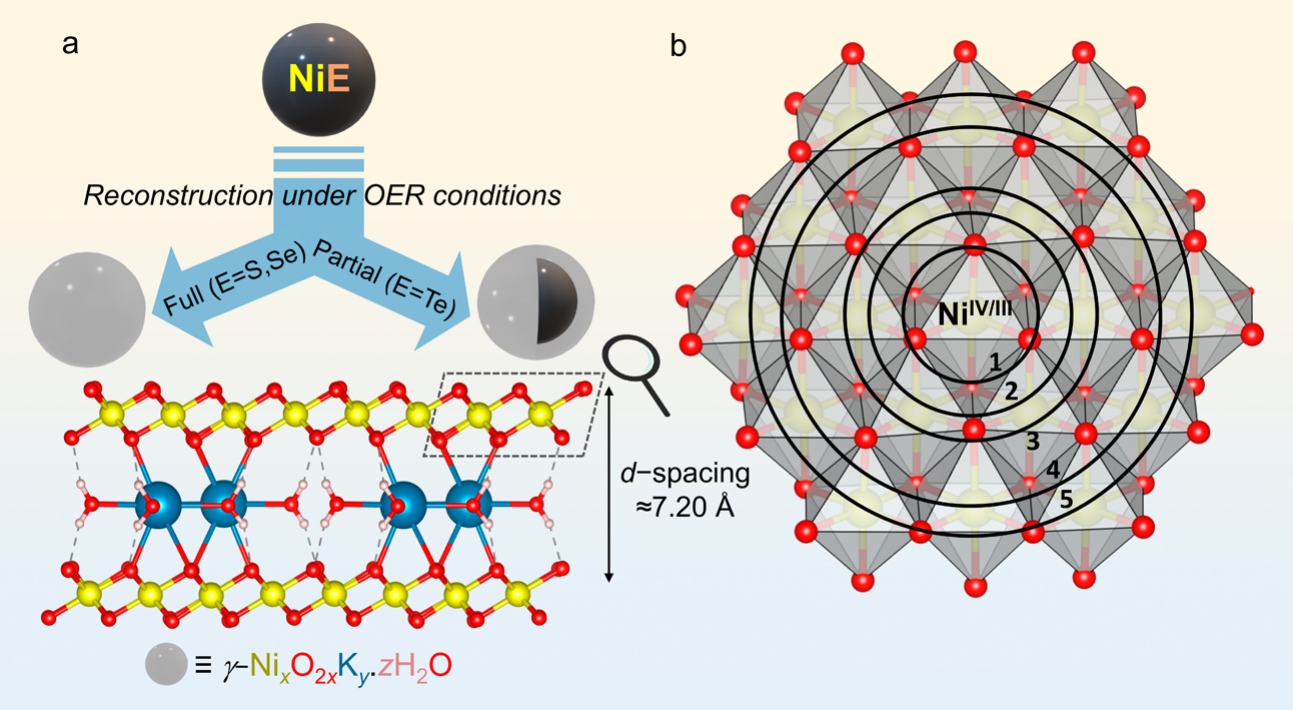
(a) Schematic illustration of the full and partial reconstructions NiS/NiSe and NiTe, respectively, under OER conditions into γ-Ni_*x*_O_2*x*_K_*y*_.*z*H_2_O active structures, with a *d*-spacing of 7.20 Å, which is a deprotonated form of the γ-NiOOH_*x*_ phase, with K^+^ and water intercalation. (b) The layers consist of edge-sharing [NiO_6_] octahedra, drawn up to five shells, with Ni in the IV and III oxidation states.

As elucidated through the post-OER characterizations, the NiE precatalysts, when subjected to alkaline OER conditions, exhibit distinct transformation behaviors. NiS and NiSe undergo rapid and full reconstruction, while NiTe experiences partial reconstruction to K^+^ intercalated γ-NiOOH_*x*_ active phases, driven by the complete leaching of S and Se, and partial leaching of Te, respectively (Figure 7a). The degree of reconstruction is significantly influenced by the type of chalcogen, with S and Se demonstrating a propensity for facile leaching, while Te exhibits resistance to oxidation and leaching possibly due to the increased metallic nature of Te which strengthens the Ni–Te bond.^12,20−22^ As validated by the *in situ* XAS analysis, the γ-NiOOH_*x*_ phase is structured with layers comprising edge-sharing [NiO_6_] octahedra, with Ni atoms in both IV and III oxidation states (Figure 7b). Dionigi et al., through DFT calculations, observed that under OER conditions, γ-NiOOH_*x*_ forms the structure Ni_*x*_O_2*x*_K_*y*_.*z*H_2_O, in which the bridging hydroxyl groups are deprotonated, forming NiO_2_ layers, and K^+^ ions and water molecules are intercalated from the KOH electrolyte.^65^ The K^+^ ions interconnect these layers through O–K–O ionic bonds and stabilize the structure. Therefore, substantiated by the detection of Ni^IV^ species in EXAFS, the observed K^+^-intercalated γ-NiOOH_*x*_ phase, in this case, should also predominantly consist of deprotonated NiO_2_ layers (Figure 7). Due to the presence of the “oxo wall” for d^6^ metal centers (like Ni^IV^), the Ni^IV^···O species possess a substantial Ni^III^–O**·** resonance character, and these oxygen radicals play a pivotal role in promoting the O–O bond formation, a critical step in the production of O_2_.^28−30^ Therefore, the availability of Ni^IV^ species is crucial for boosting OER activities. The proposed mechanism of OER is described in Figure S49 of the Supporting Information.^28−30,83^ Furthermore, we conducted a comparative assessment of the activity of the NiSe precatalyst with sacrificial anion-free Ni nanoparticles (obtained through the decomposition of the precursor **[L**^**e**^**Ni]**_**2**_**·toluene** at 250 °C, see the Supporting Information for details) and directly synthesized NiOOH (prepared using a wet chemical approach^15^) (Figures S51–S53). The results demonstrate a higher activity of NiSe precatalyst as compared to Ni nanoparticles and NiOOH, which underlines the advantage of *in situ* derived NiOOH phases from Ni precatalysts toward OER (Figure S54). Such beneficial reconstructions are also applied in industries, wherein high surface area Raney Ni is formed via the sacrificial leaching of aluminum.^12^

To understand the origin of activity difference between the materials, we normalized the geometric current densities of the Ni chalcogenides by their post-OER *C*_dl_ values and the amount of Ni redox active electrons, which revealed similar intrinsic activities for the materials (Figure S55). This result, coupled with the similar Tafel slopes and TOFs (Figure 3c, Table S10), strongly suggests the presence of identical active sites within the materials, which agrees with the same atomical structure of their active phases. Consequently, any disparities in their electrochemical activity likely originate from the availability of active sites. The number of active sites can be estimated by determining quantities directly proportional to it like the amount of Ni redox active electrons and ECSA (or *C*_dl_). The fully reconstructed NiS- and NiSe-derived γ-NiOOH_*x*_ active phases possess a higher number of active sites than the partially reconstructed NiTe-derived γ-NiOOH_*x*_ phase, explaining its lowest activity. Between NiS and NiSe, the NiSe-derived γ-NiOOH_*x*_ active phase exhibits a significantly larger amount of Ni redox active electrons and *C*_dl_ in comparison to the NiS-derived one, likely due to the larger volume of the leaching Se species, compared to S species, resulting in a more porous structure in the former (Figure 3d, Figures S21 and S23).^12^ Therefore, even though the NiS and NiSe-derived γ-NiOOH_*x*_ phases undergo complete reconstructions, the pivotal factor determining the activity of the catalysts is the number of Ni sites electronically wired to the anode and accessible to the electrolyte to actively participate in catalysis, which is higher in the NiSe-derived catalyst.

Furthermore, under harsh alkaline conditions (similar to the 1 M KOH electrolyte used in this work), the formation of electrophilic oxygen species marks the initial step for both alcohol/aldehyde and water oxidation.^84^ Therefore, similar active sites are likely responsible for catalyzing both OER and alcohol/aldehyde oxidation, which is also established in our case, wherein an analogous trend in BA oxidation and OER activity for the Ni chalcogenide materials is observed. The proposed mechanism for BA and HMF oxidations on the Ni chalcogenide-derived γ-NiOOH_*x*_ phases is illustrated in Figure S50 of the Supporting Information.^85^ For the oxidation of alcohol, such as the conversion of BA to benzoic acid, the BA molecules are first adsorbed on the surface of the catalyst and then oxidized to benzoic acid either via a hydride transfer mechanism or a hydrogen atom transfer mechanism. During the oxidation process, NiOOH is reduced to Ni(OH)_2_, which is then oxidized again, completing the catalytic cycle. For HMF oxidation, in pH > 13 conditions, the aldehyde group is first oxidized to form the 5-hydroxymethyl-2-furancarboxylic acid intermediate, followed by oxidation of the alcohol group through the 5-formyl-2-furancarboxylic acid intermediate.

## Conclusions

We have successfully designed molecular SSPs that enable a straightforward pathway for accessing nanostructured phases of the full series Ni chacogenides, that is, NiS, NiSe, and the thermodynamically challenging NiTe. Our investigations unveiled an observable trend in OER activity among these materials, with NiSe outperforming NiS and NiTe. Notably, NiSe demonstrated durability under industrially relevant conditions, maintaining its performance for 11 days. Detailed *ex situ* and quasi *in situ* Raman and XAS characterizations uncovered that, under OER conditions, NiS and NiSe undergo a complete transformation, while NiTe undergoes a partial transformation, into K^+^-intercalated γ-Ni^IV/III^OOH_*x*_ active phases. The activity difference arises from a difference in the availability of active sites, as indicated by the amount of redox active Ni electrons and *C*_dl_ (or ECSA) values. The NiSe-derived γ-NiOOH_*x*_ active phase has significantly more active sites than the NiS-derived γ-NiOOH_*x*_ phase, likely due to the larger volume of the leached Se species as compared to S, which results in a more porous active structure. Therefore, NiSe exhibits the highest activity, despite both NiSe and NiS undergoing complete reconstructions. NiTe, on the other hand, undergoes partial reconstruction, leading to fewer active sites and the lowest activity. Therefore, our results emphasize the substantial influence of the chalcogen type on the extent of reconstruction, active structure, and performance of the Ni chalcogenides. We further employed our Ni chalcogenide materials for the selective oxidation of BA and noted an activity trend similar to that for OER, reiterating that similar active sites catalyze both OER and organic oxidations. Besides, NiSe was also applied for HMF oxidation, yielding high FEs for both BA and HMF oxidations, producing industrially valuable chemicals. We anticipate that the insights provided in this work will be valuable for the development of efficient transition metal-based electrooxidation precatalysts through the low-temperature SSP approach and contribute toward the ongoing efforts aimed at deciphering the role of chalcogens in influencing the activity and reconstruction of chalcogenide-based precatalysts.

## Experimental Section

### Synthesis of the SSPs

The starting material **[L^e^Ni]_2_·toluene** (L^e^ = HC(CMeNC_6_H_3_Et_2_)_2_])^45^ and red selenium^86^ were prepared according to the literature procedures. For **[L^e^NiS]_2_**, to a Schlenk flask charged with **[L^e^Ni]_2_·toluene** (2.14 g, 2.29 mmol) and 60 mL diethyl ether, elemental sulfur (S_8_, 0.147 g, 0.574 mmol) was added at room temperature with stirring. After stirring for 2 h, the resulting dark green solution was filtered, concentrated under reduced pressure, and cooled at −20 °C overnight to give **[L^e^NiS]_2_** as black crystals (Yield: 1.95 g, 2.16 mmol, 94%). Mp 157 °C (decomp.). For **[L^e^NiSe]_2_**, to a Schlenk flask charged with **[L^e^Ni]_2_·toluene** (0.93 g, 1.00 mmol) and 30 mL diethyl ether, elemental red selenium (Se_8_, 0.157 g, 0.25 mmol) was added at room temperature with stirring. After stirring for 20 h, the resulting brown-red solution was filtered, concentrated under reduced pressure, and cooled at −20 °C overnight to give **[L^e^NiSe]_2_** as brown-red crystals (Yield: 0.90 g, 0.91 mmol, 91%). Mp 169 °C (decomp.). For **[L^e^NiTe]_2_**, to a Schlenk flask charged with **[L^e^Ni]_2_·toluene** (1.00g, 1.07 mmol) and 30 mL diethyl ether, elemental tellurium powder (Te in excess: 0.87 g, 6.82 mmol) was added at room temperature with stirring. After stirring for 3 days, the dark violet solution was filtered, concentrated under reduced pressure, and cooled at −20 °C overnight to give **[L^e^NiTe]_2_** dark brown crystals (Yield: 1.08 g, 0.98 mmol, 92%). Mp 176 °C (decomp.).

### Synthesis of the Materials

The oleylamine solvent was first degassed using a 3-cycle freeze-pump method. Then, 15 mL of the dried oleylamine was taken in a round-bottom Schlenk flask equipped with a condenser and heated to 250 °C under inert atmosphere. In a separate Schlenk flask, the precursor **[L**^**e**^**NiS]**_**2**_ (0.452 g, 0.5 mmol, for NiS)/ **[L**^**e**^**NiSe]**_**2**_ (0.500 g, 0.5 mmol, for NiSe)/ **[L**^**e**^**NiTe]**_**2**_ (0.547 g, 0.5 mmol, for NiTe)/ **[L**^**e**^**Ni]**_**2**_**·toluene** (0.467 g, 0.5 mmol, for Ni nanoparticles) was dissolved in 5 mL of dried oleylamine at 30 °C. This solution was rapidly injected into the first Schlenk flask at 250 °C under inert atmosphere, with stirring. After stirring for 1 h at 250 °C, the reaction mixture was allowed to cool to room temperature. The solution was then centrifuged which yielded a black solid precipitate. The product was washed thrice with a 1:1 mixture of ethanol and hexane to remove any impurities, including excess ligand and oleylamine, and then dried overnight at 60 °C. NiOOH was synthesized according to the literature report.^15^

### Electrochemical Measurements

The typical electrochemical measurements were performed in a standard three-electrode (working, counter, and reference) cell using a potentiostat (SP-200, BioLogic Science Instruments) equipped with the EC-Lab v10.20 software package. The catalysts deposited on NF and FTO substrates were used as the working electrode, Pt wire as the counter electrode, Hg/HgO as the reference electrode, and 1 M aqueous KOH as the electrolyte. Cyclic voltammetry CV), linear sweep voltammetry (LSV), chronoamperometry (CA), and chronopotentiometry (CP) were measured with an *iR* compensation of 90%. The potentials are reported with respect to the reversible hydrogen electrode (RHE) in 1 M aqueous KOH (pH = 13.89)^87^ using *V*_RHE_ = *V*_Hg/HgO_ + *V*°_Hg/HgO_ + (0.000198 × *T* × pH) V K^–1^, where *V*_RHE_ is the potential of the working electrode vs RHE, *V*_Hg/HgO_ is the potential of the working electrode vs Hg/HgO reference electrode, *V*°_Hg/HgO_ is the standard electrode potential of the Hg/HgO reference electrode, which is 0.098 V, and *T* is the temperature of the system, which is 298 K. The details of Tafel slopes, TOF, EIS, FE, and industrially relevant water-splitting are described in the Supporting Information.

### Electrocatalytic Oxidation of Organic Substrates

The electrocatalytic oxidation of BA and HMF were performed in a three-electrode undivided cell. The catalysts deposited on NF were used as the working electrode, Pt wire as the counter electrode, Hg/HgO electrode as the reference electrode and 15 mL of 1 M aqueous KOH containing 0.1 M BA or 0.1 M HMF as the electrolyte. LSVs were recorded at 5 mV/s without stirring. Bulk electrolyses were performed using CAs at 1.46 *V*_RHE_, with stirring at 350 rpm. The BA and HMF oxidation products were quantified using ^1^H NMR, by calculating the relative intensity of the proton signals of the reaction mixture. The product yield and FE were calculated using the following equations: product yield (%) = [*n*_product_/*n*_reactant_] × 100%, FE (%) = [(*n*_product_·*n*_e_·*F*/*Q*)] × 100%, where *F* is the Faraday constant (96485 C/mol), *n*_reactant_ is the initial number of moles of the reactant (1.5 mmol), *n*_product_ is the number of moles of product quantified from ^1^H NMR, *n*_e_ is the number of electrons needed for the oxidation process (which is 4 for BA to benzoic acid oxidation and 6 for HMF to 2,5 furandicarboxylic acid oxidation), and *Q* is the charge in Coulombs passed through the solution. The ^1^H NMR data are provided in the Supporting Information.

## Supporting Information Available

General considerations, chemicals and materials, characterization methods, additional characterization of the molecular precursors and the materials before and after OER, additional OER measurements, and ^1^H NMR analysis of the organic oxidation reactions (PDF)
