# Supplementary material for: A Knowledge-Based Molecular Single-Source Precursor Approach to Nickel Chalcogenide Precatalysts for Electrocatalytic Water, Alcohol, and Aldehyde Oxidations
Source: ACS Nano. 2024 Dec 3;18(50):33964–76. doi: 10.1021/acsnano.4c08058 (PMC11656844; doi:10.1021/acsnano.4c08058)
Supplement: Supplementary file 1 — nn4c08058_si_001.pdf [file nn4c08058_si_001.pdf]

Supporting Information

A Knowledge-Based Molecular Single-Source Precursor Approach to Nickel Chalcogenide Precatalysts for Electrocatalytic Water, Alcohol and Aldehyde Oxidations

Basundhara Dasgupta,[§] Shenglai Yao,[§] Indranil Mondal,[§†] Stefan Mebs,[‡] Johannes Schmidt,[£] Konstantin Laun,[fi] Ingo Zebger,[fi] Holger Dau,[‡] Matthias Driess,[§] and Prashanth W. Menezes[§¶]**

AUTHOR ADDRESS

[§]Department of Chemistry: Metalorganics and Inorganic Materials, Technische Universität Berlin, Straße des 17. Juni 115, Sekr. C2, 10623 Berlin, Germany

Email: matthias.driess@tu-berlin.de, prashanth.menezes@mailbox.tu-berlin.de

[†]School of Chemistry, Indian Institute of Science Education and Research, Thiruvananthapuram, Kerala 695551, India

[‡]Department of Physics, Freie Universität Berlin, Arnimallee 14, 14195 Berlin, Germany

[£]Department of Chemistry: Functional Materials, Technische Universität Berlin, Hardenbergstraße 40, 10623 Berlin, Germany

[fi]Department of Chemistry: Physical Chemistry/Biophysical Chemistry, Technische Universität Berlin, Straße des 17 Juni 135, Sekr. PC14, 10623 Berlin, Germany

[¶]Materials Chemistry Group for Thin Film Catalysis – CatLab, Helmholtz-Zentrum Berlin für Materialien und Energie, Albert-Einstein-Straße 15, 12489 Berlin, Germany

E-mail: prashanth.menezes@helmholtz-berlin.de

General considerations

Chemicals and reagents (analytical grade) were used as purchased without any further purification. Deionized water was used throughout the experiments. The synthesis and characterization of molecular complexes were carried out under inert conditions using moisture and oxygen-free standard Schlenk techniques or an MBraun glove box fitted with a gas purification and recirculation unit. Solvents were dried by standard methods and freshly distilled prior to use. The starting material **[L^{e}Ni]$_2$toluene** (L^e = HC(CMeNC$_6$H$_3$Et$_2$)$_2$])[1] and red selenium[2] were prepared according to the literature procedures.

Chemicals and materials

Elemental sulfur and tellurium were obtained from Sigma Aldrich. Benzyl alcohol (BA, 99%) was obtained from ACROS Organics. 1 M aqueous potassium hydroxide (KOH; Fe<0.05 ppm determined by ICP-OES) and 5-Hydroxymethyl-2-furaldehyde (HMF, 97%) were purchased from Thermo Fischer Scientific. The electrode substrate nickel foam (NF) and fluorine-doped tin oxide (FTO, resistivity 8−12 Ω/sq) were obtained from Recemat BV and Sigma Aldrich, respectively. The Pt wire counter electrode (0.5 mm diameter × 230 mm length; A-002234) was purchased from BioLogic and the Hg/HgO reference electrode was purchased from CH Instruments, Inc.

Characterization methods

Nuclear Magnetic Resonance (NMR) spectroscopy. NMR spectra were recorded with Bruker spectrometers ARX200 and AV400 referenced to residual solvent signals as internal standards. Abbreviations: *s* = singlet; *d* = doublet; *t* = triplet; *sept* = septet; *m* = multiplet; *br* = broad.

Elemental Analyses. Elemental analyses were performed by the analytical labor service at the Institute of Chemistry, Technical University (TU) of Berlin, Germany.

High Resolution Electrospray Ionization Mass Spectroscopy (HR ESI-MS). HR ESI-MS were measured on a Thermo Scientific LTQ orbitrap XL.

Fourier-transform Infrared spectroscopy (FT-IR). FT-IR spectra were measured with a Nicolet iS5 FT-IR-Spectrometer from the company of Thermo Scientific.

Single-Crystal X-ray Diffraction (SC-XRD). The crystal was mounted on a glass capillary in per-fluorinated oil and measured in a cold N_2 flow. The data of compounds **[L^{e}NiS]$_2$**, **[L^{e}NiSe]$_2$**, and **[L^{e}NiTe]$_2$** was collected on an Oxford Diffraction Supernova, Single source at offset, Atlas at 150 K (Cu- Kα-radiation, λ = 1.5418 Å). The structures were solved by direct methods with the program SHELXT[3] and refined with Olex2.[4] In the molecule structure of **[L^{e}NiS]$_2$**, the Ni_2S_2 moiety was disordered over two positions in a ratio of 0.50:0.50. In the molecule structure of **[L^{e}NiSe]$_2$**, the Ni_2Se_2 moiety was disordered over two positions in a ratio of 0.93:0.07. Two independent molecules are observed in the asymmetric unit for **[L^{e}NiTe]$_2$**. In one of the latter molecules, the Ni_2Te_2 moiety was disordered over two positions in a ratio of 0.88:0.12.

Powder X-ray Diffraction (PXRD). A Panalytical X'Pert PRO diffractometer (Bragg-Brentano geometry, Cu-Kα radiation) was used for PXRD measurements.

Inductively Coupled Plasma Optical Emission Spectroscopy (ICP-OES). ICP-OES was carried out on a Thermo Jarrell Ash Trace Scan analyzer. The presented materials were digested in 1:3 HNO_3: HCl v/v (nitric acid, SUPRA-Qualität ROTIPURAN® Supra 69%, and hydrochloric acid, SUPRA-Qualität ROTIPURAN® Supra 30%), and the average content of three reproducible independent experiments were presented. The digestion volume (1 mL) was diluted with Milli-Q water up to 15 mL. Calibration curves were prepared for nickel, sulfur, selenium, and tellurium with concentrations between 0.1 mg/L and 100 mg/L from standard solutions (1000 mg/L single element ICP-Standard Solution ROTI®STAR).

Electron Microscopy. To gain more insight into the surface morphology and structures of our samples, scanning electron microscopy (SEM) was performed in a Zeiss Gemini 500 microscope integrated with energy-dispersive X-ray (EDX) (EDAX, Apollo XPP). Data handling and analysis were attained with the software package EDAX. The nanostructure of the samples was further investigated using an FEI Tecnai G2 20 S-TWIN (FEI Company, Eindhoven, Netherlands) transmission electron microscopy (TEM), equipped with a LaB_6 source at 200 kV acceleration voltage. For the measurement of the sample after electrocatalysis, the catalyst films were scraped from the FTO electrode substrate, dispersed in acetone, and drop-casted onto a carbon-coated copper grid. The images were recorded with a GATAN MS794 P CCD camera. The SEM and TEM experiments were conducted at the Zentrum für Elektronenmikroskopie (ZELMI) of the TU Berlin.

X-ray Photoelectron Spectroscopy (XPS). The X-ray photoelectron spectroscopy (XPS) measurements were conducted on a Kratos Axis Ultra X-ray photoelectron spectrometer (Kratos Analytical Ltd., Manchester, U.K.) using an Al-Kα monochromatic radiation source (1486.7 eV) with 90° takeoff angle (normal to analyzer). The vacuum pressure in the analyzing chamber was kept at 2×10^{-9} Torr. The XPS spectra were collected for Ni 2p, S 2p, Se 3d and Te 3d levels with pass energy 20 eV and step 0.1 eV. The binding energies were calibrated relative to the C 1s peak energy position as 285.0 eV. Data analyses were carried out using Casa XPS (Casa Software Ltd.) and the Vision data processing program (Kratos Analytical Ltd.).

Raman spectroscopy. Quasi *in situ* and *ex situ* Raman spectra were recorded using the 458 nm emission of an Argon ion laser (Innova 70, Coherent) for excitation and a confocal Raman spectrometer (Lab Ram HR- 800 Jobin Yvon) equipped with a liquid-N_2 cooled charge-coupled device (CCD) camera for data acquisition. The typical laser power at the sample was 1 mW. An average spectrum was obtained by recording three consecutive scans for 240 s in a single spot. Measurements were performed using a Linkam Cryostage THMS600 cryostat associated with the sample holder, in which the film temperature was kept at 80 K throughout the measurements. These were conducted in sum at three different spots of the film and were consistent with respect to the monitored peak positions. Quasi *in situ* experiments were performed after treating the materials at chronopotentiometry (CP) at 10 mA/cm^2 for 24 h and then freeze quenching at 1.56 V_{RHE}. The films were freeze-quenched using liquid N_2 under vigorous Ar gas flow and stored in liquid N_2.

X-ray Absorption Spectroscopy (XAS). To get insights on the local atomic and electronic structure X-ray absorption near-edge structure (XANES) and extended X-ray absorption fine-structure (EXAFS), studies were carried out under quasi *in situ* and *ex situ* conditions. The XAS spectra (XANES/ EXAFS) were recorded at the BESSY II synchrotron radiation source operated by the Helmholtz-Zentrum Berlin. The measurements were performed at the KMC-3 bending-magnet beamline at 20 K in a helium-flow cryostat (Oxford-Danfysik). The incident beam energy was selected by a Si(111) double-crystal monochromator. The measurements at the nickel and selen K-edges were performed in fluorescence mode ($K_{\langle}$ fluorescence energy region selected) using a 13 elements silicon drift detector (Rayspec). Over 20 spectra were averaged for each compound to improve the signal-to-noise ratio. Averaged spectra were background-corrected and normalized using in-house software. The extracted spectra were weighted by k^3 and simulated in *k*-space (E_0 = 8333 eV for Ni, 12658 eV for Se). The XANES edge position was determined by the integration method. All EXAFS simulations were performed using in-house software (SimXLite, programmed by Dr. Petko Chernev) after calculation of the phase functions with the FEFF program (version 8.4, self-consistent field option activated). The data range used in the simulation of the EXAFS spectra was k = (2-14) $Å^{-1}$. The EXAFS simulation was optimized by a minimization of the gg sum obtained by the summation of the squared deviations between measured and simulated values (least-squares fit). The fit was performed using the Levenberg-Marquardt method with numerical derivatives. The error ranges of the fit parameters were estimated from the covariance matrix of the fit. Cosine windows covering 10% of the low *k*-side and 10% of the high *k*-side of the spectra were applied for the calculation of the Fourier transforms. Further details are given elsewhere. Quasi *in situ* experiments were performed after treating the materials at CP at 10 mA/cm^2 for 24 h and then freeze quenching at 1.56 V_{RHE}. The films were freeze-quenched using liquid N_2 under vigorous Ar gas flow and stored in liquid N_2.

Electrophoretic deposition (EPD) on substrates

The investigated materials were deposited on both, NF and FTO, electrophoretically, by applying a potential difference of 10 V in a mixture of iodine and acetone on a 1 × 1 cm^2 area. For the typical deposition protocol, 20 mg of the catalyst powder was suspended in 10 ml acetone, and 3 mg of iodine was then added. This solution was agitated in an ultrasonic bath for 30 min. Before EPD, the empty electrodes were weighed using an analytical balance, and the weights were noted. Thin uniform films were achieved by applying a potential of 10 V for 30-180 s by stirring the solution continuously at room temperature. After each EPD, the increase in weight of the electrodes was monitored carefully. For each catalyst, the EPD time was adjusted and a loading of ≈0.9 mg/cm^2 and ≈0.5 mg/cm^2 on NF and FTO was obtained, respectively. The mass loading was reproducible within the margins of an experimental error (± 0.1 mg).

Electrochemical measurements

The steady-state Tafel slopes were determined by potentiostatic measurements employing stepwise changes of the potential by 15 mV. The current density at each potential was measured for a constant potential for 300 s and the average current values were used for the determination of Tafel plots. The Tafel slope was calculated according to the Tafel equation:

$$\eta = \mathrm{b}\log j + \mathrm{a}$$

where η is the overpotential, b is the Tafel slope, j is the current density, and a is a variable proportional to the logarithm of the exchange current density.

A qualitative idea of the electrochemically active surface area of the catalyst films was obtained by determining their double-layer capacitances (C_{dl}) from the CV (cycled between 0.895 and 0.955 V_{RHE}), where no apparent faradaic process occurred. Half of the potential difference at 0.925 V_{RHE} were plotted as a function of the scan rate and from the slope, the C_{dl} was estimated.

Electrochemical impedance spectroscopy (EIS) was recorded at 1.56 V_{RHE} to obtain the Nyquist plots. The amplitude of the sinusoidal wave was determined in a frequency range of 100 kHz to 1 mHz. The plots were fitted using a simple Randles circuit with a constant phase element.

Turnover Frequency (TOF) was calculated according to the following equation:

$$\mathrm{TOF} = (j \cdot \mathrm{A}) / (\mathrm{z} \cdot \mathrm{F} \cdot \mathrm{m})$$

where j is the OER current density at 1.63 V_{RHE}, A is the geometrical surface area of the electrode (1 cm^2), z is the unitless number of electrons needed to form O_2 (4), F is the faradaic constant (96485 C/mol), and m is the amount of redox active Ni sites, which were calculated based on the equations of Figure S21 and under the assumption that 1.6 electrons per active nickel site were transferred during the redox process.[5]

The Faradaic Efficiency (FE) of NiSe in 1 M aqueous KOH towards OER was measured on NF in a closed two-electrode cell. The cell comprised of two burettes, which were connected using a glass pipe. NiSe/NF anode was fixed in one burette while the Pt wire cathode was fixed in the other burette. A constant current density of 50 mA/cm^2 was applied for 1 hour. A stoichiometric H_2 and O_2 gases were generated in the headspace of the cathode and anode burette, respectively, and resulted in a decrease in water volume in the burettes. The FE is calculated based on:

$$FE\ (O_2,\%) = [(4 \cdot F \cdot p \cdot V_{O2}) / (R \cdot T \cdot j \cdot t)] \times 100\%$$

where F is the Faraday constant (96485 C/mol), p is the pressure (101 kPa), V_{O2} is the evolved volume of oxygen, which is equal to the amount of water volume decrease in the anode burette after CP, R is the ideal gas constant (8.314 J/mol-K), T is the temperature (298 K), *j* is the current density (the potentiostat was set to 50 mA/cm^2 but the real current that was measured on average by the potentiostat was only 49.3 mA/cm^2), and t is the period of electrolysis (3600 s).

The overall water-splitting measurements under industrial conditions were carried out in a two-electrode closed cell, with NiSe/NF as the anode and Pt wire as the cathode, in 6 M KOH. LSVs were recorded at both room temperature (25 °C) and a high temperature (65 °C). CP was recorded at 500 mA/cm^2 in 6 M KOH, at 65 °C, with stirring at 350 rpm. The measurements were recorded without any *iR* correction.

Characterization of compounds [L^{e}NiE]$_2$ (E = S, Se, Te)

Characterization of [L^{e}NiS]$_2$. ^{1}H NMR (200 MHz, D$_6$-benzene, 298K): δ = 1.18 (s, 12 H, β-C*H*$_3$), 1.39 (t, 24H, $^3J_{H,H}$ = 7.5 Hz, NCH$_2$C*H*$_3$), 2.81 (sept, 8H, $^3J_{H,H}$ = 7.5 Hz, NC*H*$_2$CH$_3$), 2.91 (sept, 8H, $^3J_{H,H}$ = 7.5 Hz, NC*H*$_2$CH$_3$), 4.78 (s, 2 H, γ-C*H*), 6.78-6.92 (m, 12 H, Ph-*H*) ppm. **^{13}C{^{1}H} NMR** (50 MHz, D$_6$-benzene, 298K): δ = 13.9 (s, NCH$_2$*C*H$_3$), 21.2 (s, β-*C*H$_3$), 24.0 (s, N*C*H$_2$CH$_3$), 98.9 (s, γ-*C*H), 124.8, 125.6, 135.1, 154.0, 159.2 (s, β-*C*, Ph-*C*) ppm. **HR ESI-MS** (Ion spray voltage 5kV, flow rate 5µL/*min*, in THF): m/z: calculated for [M+H]$^+$($C_{50}H_{67}N_4Ni_2S_2$): 903.35086, found: 903.35069. **Elemental analysis** calcd for $C_{50}H_{66}N_4Ni_2S_2$: C 66.39, H 7.35, N 6.19; found: C 66.02, H 7.41, N 6.08; **IR** (cm^{-1}): 634(w), 718(w), 750 (s), 762(s), 802(m), 863(m), 897(w), 965(w), 1024(m), 1061(w), 1106(w), 1184(m), 1261(m), 1326(m), 1388(vs), 1442(s), 1525(s), 2872(w), 2930(w), 2960(w), 3063(w).

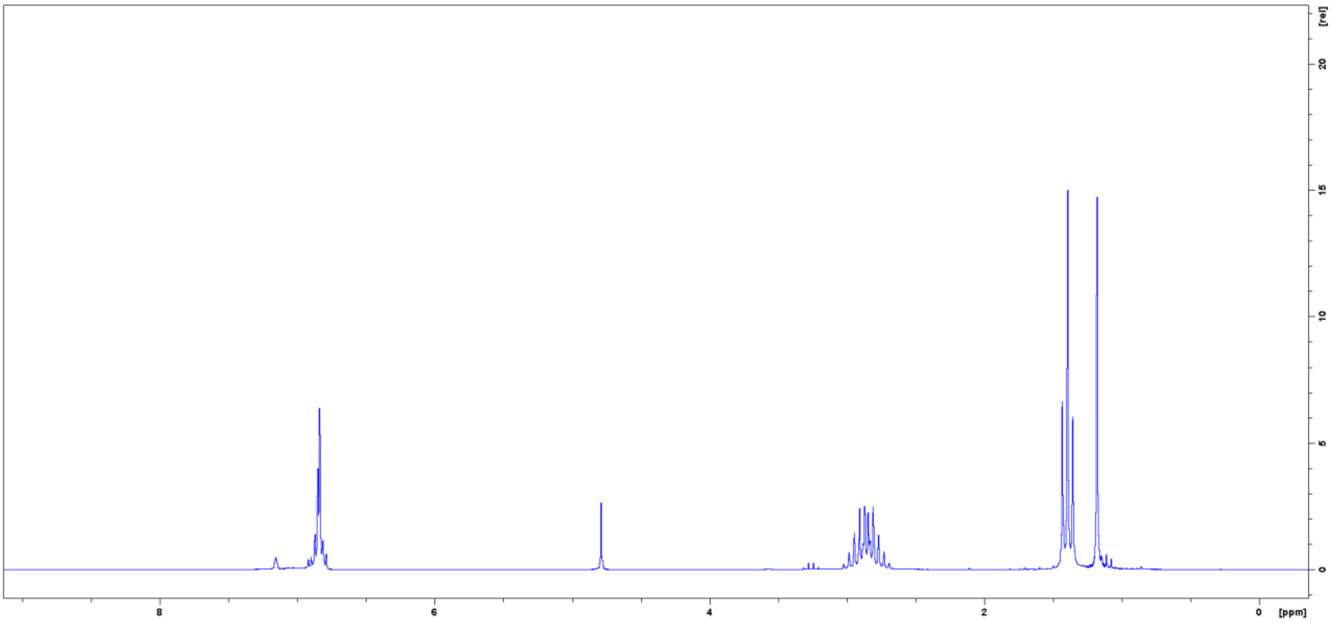

Figure S1. ^{1}H NMR spectrum of **[L^{e}NiS]$_2$** (200 MHz, D$_6$-benzene, 298K).

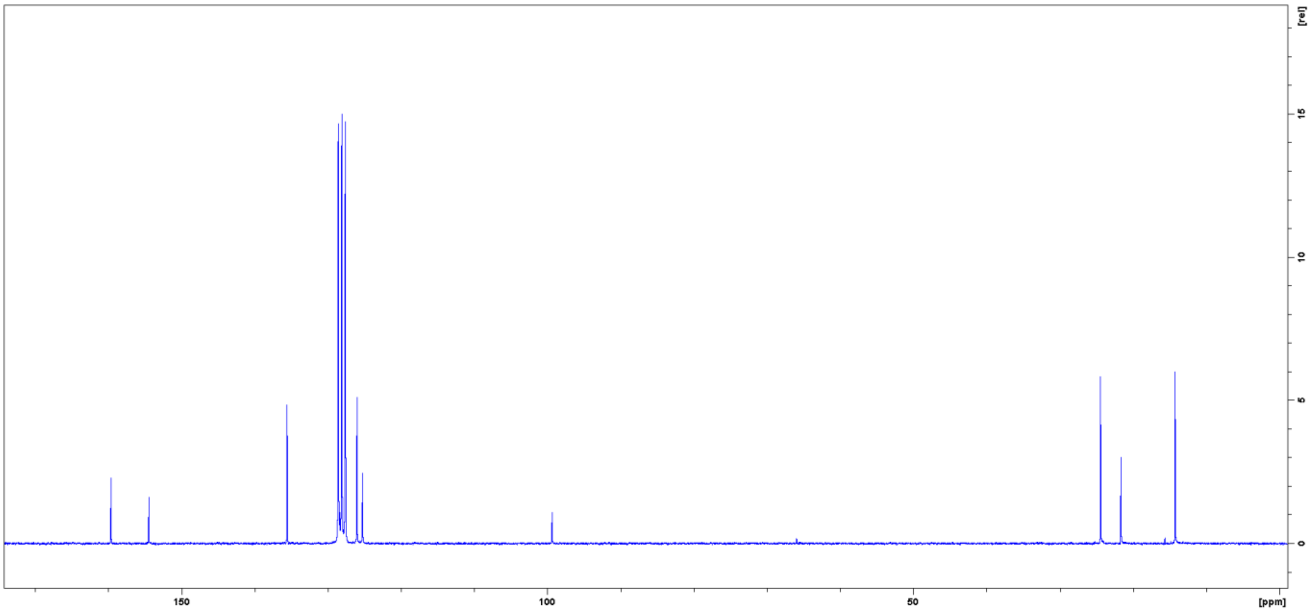

Figure S2. $^{13}C\{^{1}H\}$ NMR spectrum of **$[L^{e}NiS]_2$** (50 MHz, D_6-benzene, 298K).

Characterization of [L^{e}NiSe]$_2$. **^{1}H NMR** (200 MHz, D_6-benzene, 298K): δ = 1.19 (s, 12 H, β-C*H*$_3$), 1.42 (t, 24H, $^3J_{H,H}$ = 7.5 Hz, NCH$_2$C*H*$_3$), 2.84 (sept, 8H, $^3J_{H,H}$ = 7.5 Hz, NC*H*$_2$CH$_3$), 2.93 (sept, 8H, $^3J_{H,H}$ = 7.5 Hz, NC*H*$_2$CH$_3$), 4.80 (s, 2 H, γ-C*H*), 6.78-6.91 (m, 12 H, Ph-*H*) ppm. **^{13}C{^{1}H} NMR** (50 MHz, D_6-benzene, 298K): δ = 14.0 (s, NCH$_2$*C*H$_3$), 21.2 (s, β-*C*H$_3$), 24.3 (s, N*C*H$_2$CH$_3$), 99.8 (s, γ-*C*H), 124.9, 125.8, 135.0, 156.0, 159.2 (s, β-*C*, Ph-*C*) ppm. **HR ESI-MS** (Ion spray voltage 5kV, flow rate 5µL/*min*, in THF): m/z: calculated for $[M+H]^+$($C_{50}H_{67}N_4Ni_2Se_2$): 999.23976, found: 999.23824. **Elemental analysis** calcd for $C_{50}H_{66}N_4Ni_2Se_2$: C 60.15, H 6.66, N 5.61; found: C 59.89, H 6.52, N 5.46; **IR** (cm^{-1}): 638(w), 716(w), 750 (s), 761(s), 800(m), 861(m), 897(w), 964(w), 1022(m), 1060(w), 1106(w), 1183(m), 1260(m), 1326(m), 1392(vs), 1441(s), 1524(s), 2871(w), 2929(w), 2959(w), 3061(w).

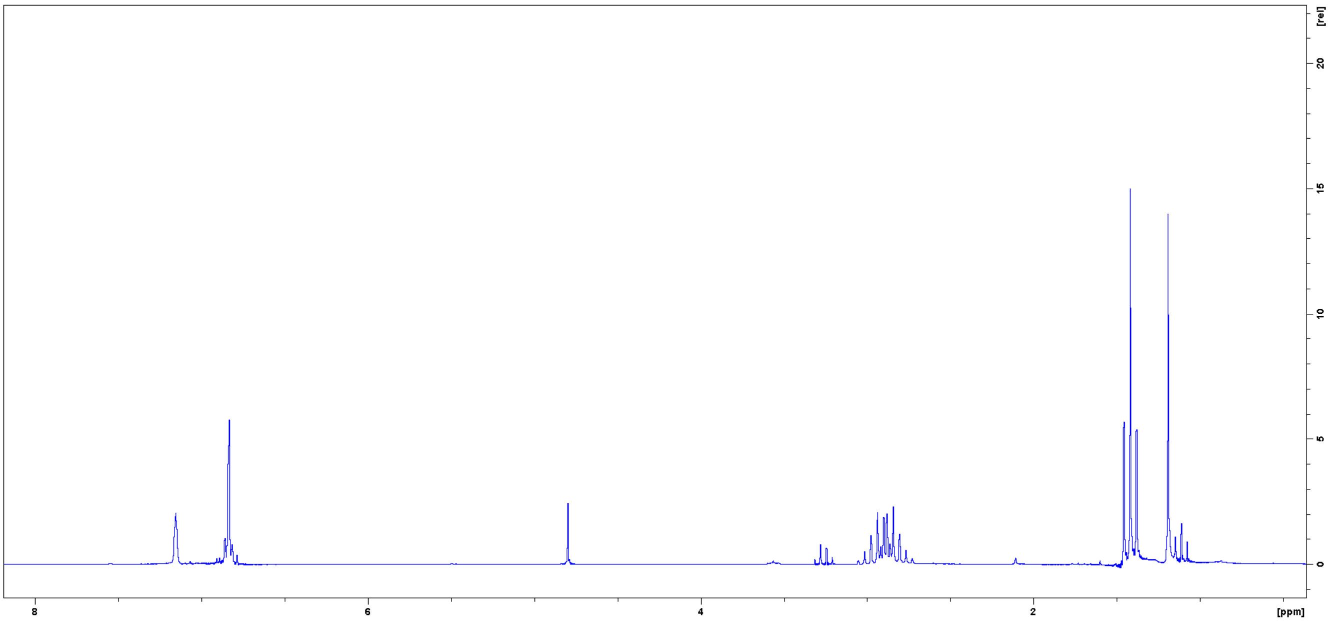

Figure S3. ^{1}H NMR spectrum of **[L^{e}NiSe]$_2$** (200 MHz, D_6-benzene, 298K).

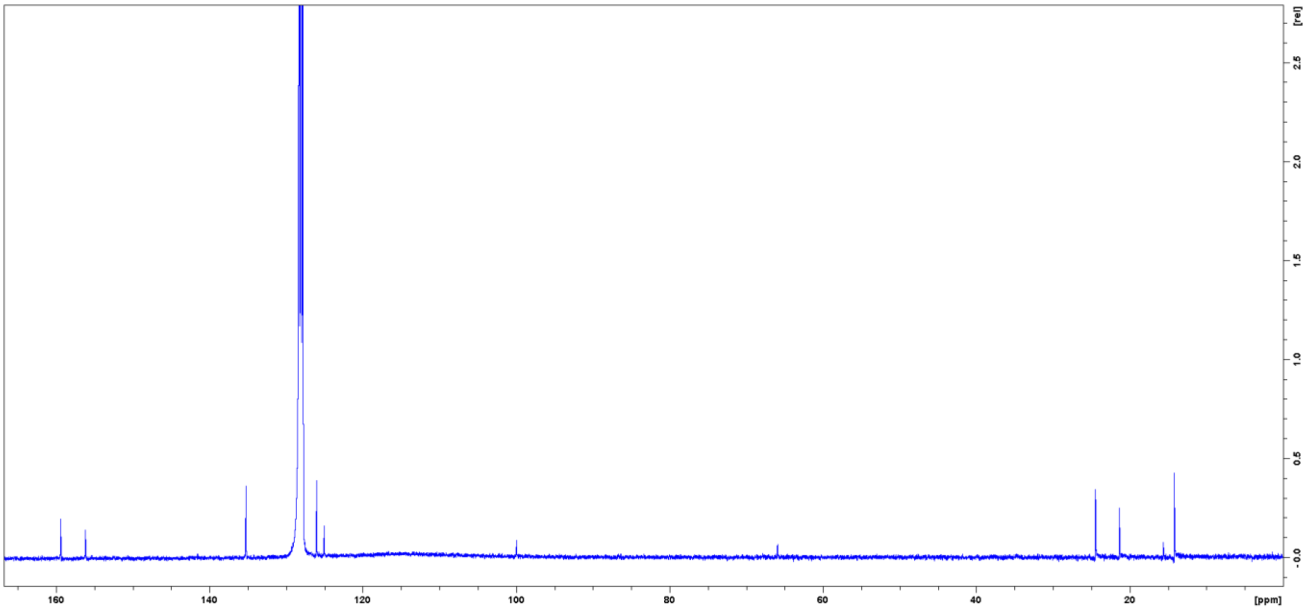

Figure S4. $^{13}C\{^{1}H\}$ NMR spectrum of **[L^{e}NiSe]$_2$** (50 MHz, D_6-benzene, 298K).

Characterization of [L^{e}NiTe]$_2$. ^{1}**H NMR** (200 MHz, D_6-benzene, 298K): δ = 1.20 (s, 12 H, β-C*H*$_3$), 1.39 (t, 24H, $^3J_{H,H}$ = 7.6 Hz, NCH$_2$C*H*$_3$), 2.85 (sept, 8H, $^3J_{H,H}$ = 7.6 Hz, NC*H*$_2$CH$_3$), 2.92 (sept, 8H, $^3J_{H,H}$ = 7.6 Hz, NC*H*$_2$CH$_3$), 4.74 (s, 2 H, γ-C*H*), 6.78-6.90 (m, 12 H, Ph-*H*) ppm. ^{13}**C{^{1}H} NMR** (50 MHz, D_6-benzene, 298K): δ = 13.8 (s, NCH$_2$*C*H$_3$), 21.3 (s, β-*C*H$_3$), 24.3 (s, N*C*H$_2$CH$_3$), 100.8 (s, γ-*C*H), 124.5, 125.8, 134.9, 158.6, 158.8 (s, β-*C*, Ph-*C*) ppm. **HR ESI-MS** (Ion spray voltage 5kV, flow rate 5µL/*min*, in THF): m/z: calculated for $[M+H]^+$($C_{50}H_{67}N_4Ni_2Te_2$): 1097.21740, found: 1097.21551. **Elemental analysis** calcd for $C_{50}H_{66}N_4Ni_2Te_2$: C 54.81, H 6.07, N 5.11; found: C 54.56, H 5.91, N 4.97; **IR** (cm^{-1}): 634(w), 722(w), 749 (s), 761(s), 897(m), 857(w), 867(w), 939(w), 1021(m), 1059(w), 1104(w), 1165(w), 1181(m), 1261(m), 1324(m), 1389(vs), 1439(s), 1521(s), 2871(w), 2927(w), 2961(w), 3056(w).

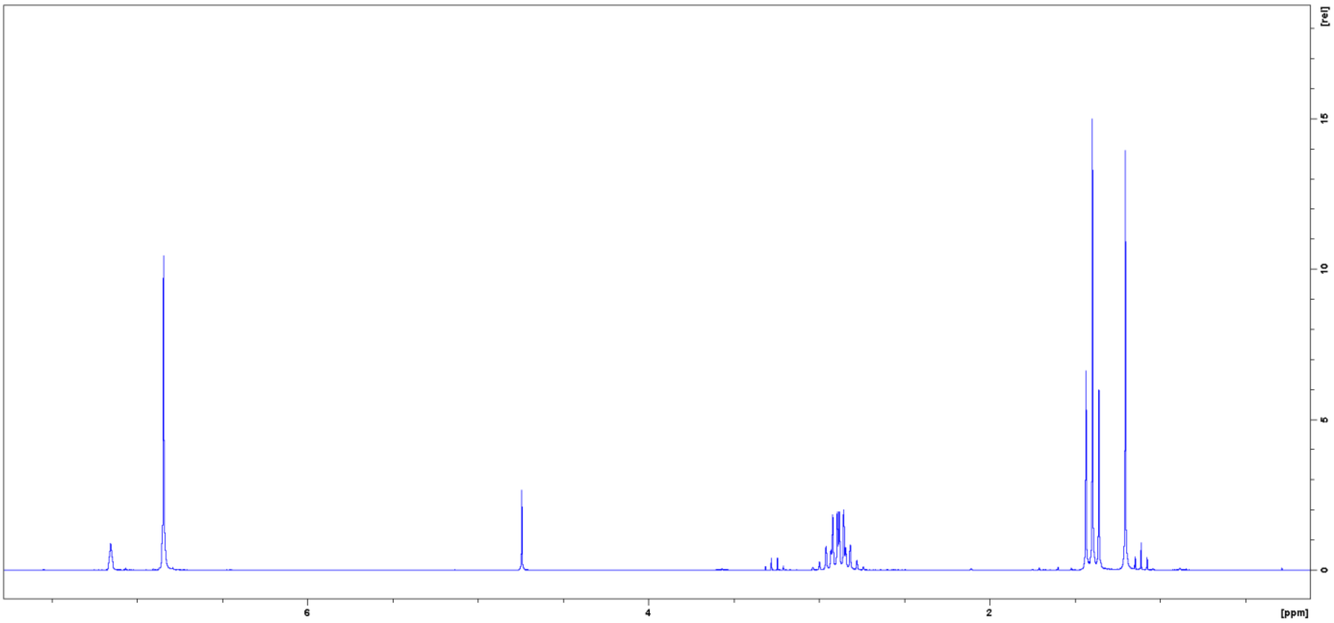

Figure S5. ^{1}H NMR spectrum of **[L^{e}NiTe]$_2$** (200 MHz, D_6-benzene, 298K).

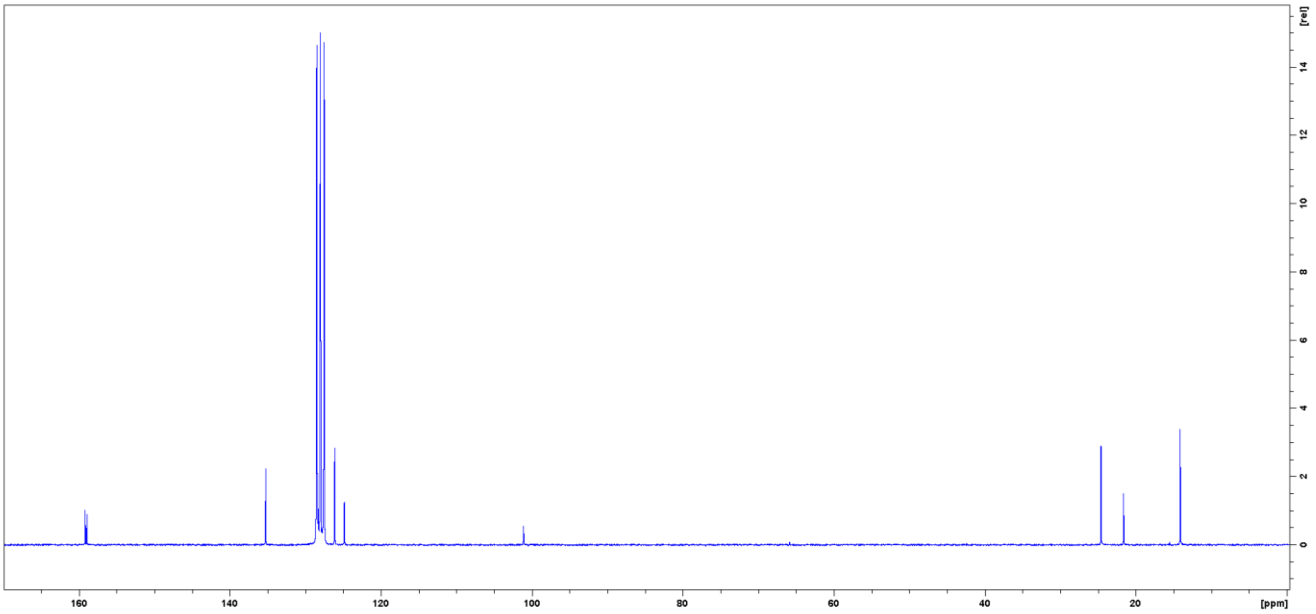

Figure S6. $^{13}C\{^{1}H\}$ NMR spectrum of **[L^eNiTe]₂** (50 MHz, D_6-benzene, 298K).

SC-XRD structure determination of compounds [L^{e}NiE]$_2$

In the molecular structure of compound **[L^{e}NiS]$_2$**, the Ni_2S_2 moiety is disordered over two orientations with an occupancy ratio of 0.50:0.50. Four of the ethyl groups are disordered over two orientations with different occupancy ratios. In the molecular structure of compound **[L^{e}NiSe]$_2$**, the Ni_2Se_2 moiety is disordered over two orientations with an occupancy ratio of 0.93:0.07. Two of the ethyl groups are disordered over two orientations with occupancy ratios of 0.58:0.42 and 0.60:0.40, respectively. In the molecular structure of compound **[L^{e}NiTe]$_2$**, two independent molecules are present in the asymmetric unit. While one of the molecules is without any disorder, in the other one, the Ni_2Te_2 moiety is disordered over two orientations with an occupancy ratio of 0.88:0.12. Two of the ethyl groups are disordered over two orientations with an occupancy ratio of 0.57:0.43.

The CCDC numbers for the three compounds are: **2334184** for **[L^{e}NiS]$_2$**, **2334185** for **[L^{e}NiSe]$_2$**, and **2334186** for **[L^{e}NiTe]$_2$**. This data contains the supplementary crystallographic data for this paper and is provided free of charge by The Cambridge Crystallographic Data Centre.

Table S1. Crystal data and structure refinement for **[L^e^NiS]₂** (CCDC: 2334184).

Empirical formula	$C_{50}H_{66}N_4Ni_2S_2$
Formula weight	904.60
Temperature	150.15 K
Wavelength	1.54184 Å
Crystal system	Monoclinic
Space group	*P* 1 *n* 1
Unit cell dimensions	a = 13.5475(3) Å, α = 90°
	b = 12.0627(2) Å, β = 109.960(2)°
	c = 15.1587(3) Å, γ = 90°
Volume	2328.42(8) $Å^3$
Z	2
Density (calculated)	1.290 Mg/m^3
Absorption coefficient	2.121 mm^{-1}
F(000)	964
Crystal size	0.32 x 0.25 x 0.09 mm^3
Theta range for data collection	3.664 to 72.636°.
Index ranges	$-16<=h<=15$, $-14<=k<=9$, $-18<=l<=18$
Reflections collected	16744
Independent reflections	7828 [R(int) = 0.0247]
Completeness to theta = 67.684°	100.0%
Absorption correction	Semi-empirical from equivalents
Max. and min. transmission	1.00000 and 0.35840
Refinement method	Full-matrix least-squares on F^2
Data / restraints / parameters	7828 / 94 / 592
Goodness-of-fit on F^2	1.056
Final R indices [I>2sigma(I)]	R_1 = 0.0517, wR_2 = 0.1356
R indices (all data)	R_1 = 0.0653, wR_2 = 0.1510
Absolute structure parameter	0.57(6)
Extinction coefficient	n/a
Largest diff. peak and hole	0.567 and -0.472 e.$Å^{-3}$

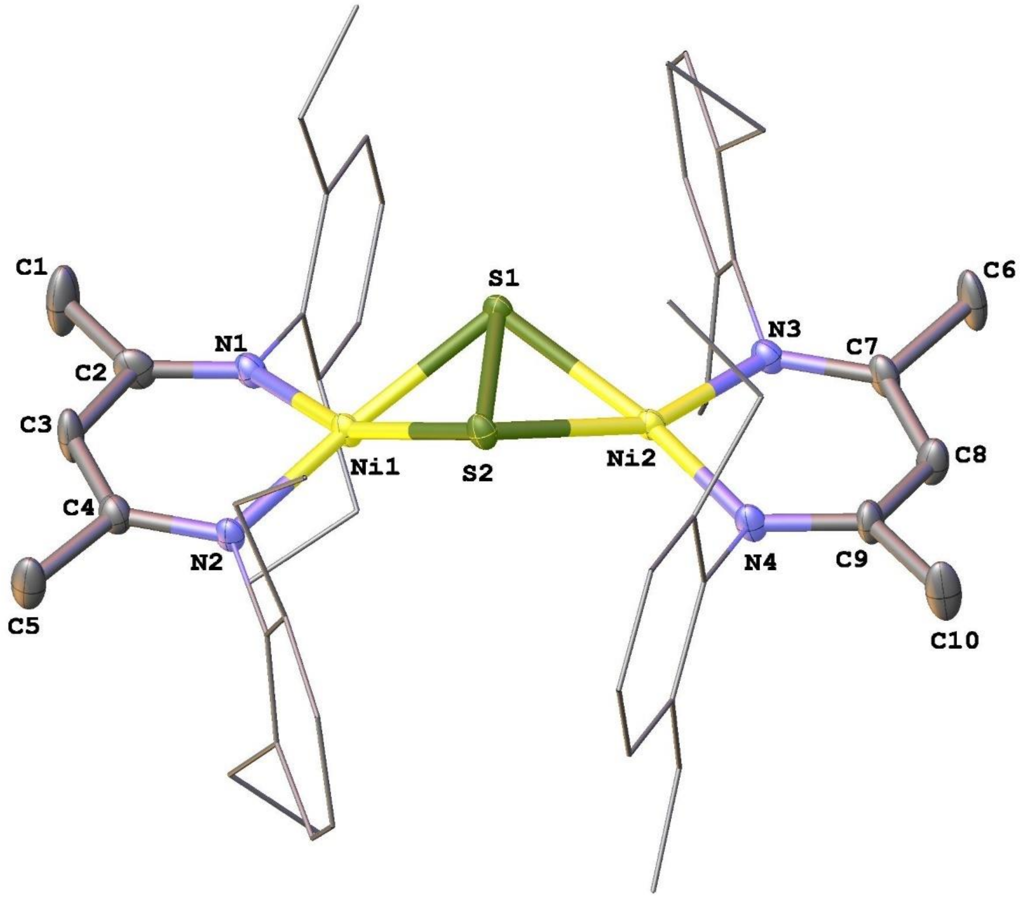

Figure S7. Molecular structure of compound **[L^eNiS]₂** (The Ni_2S_2 moiety is disordered over two orientations with an occupancy ratio of 0.50:0.50. Only one set is depicted). Thermal ellipsoids are drawn at a 30% probability level. H atoms are omitted for clarity. Selected bond lengths (Å) and angles (°): S1−S2 2.047(6), Ni1−S1 2.176(6), Ni1−S2 2.197(6), Ni2−S1 2.191(6), Ni2−S2 2.189(6), Ni1−S1−Ni2 104.8(2), Ni1−S2−Ni2 104.2(2), dihedral angle between the two NiS_2 planes: 126.93°.). As expected, the S−S distance in **$[L^{e}NiS]_2$** (2.047(6) Å) is very close to the corresponding distance observed in $[L^{D}NiS]_2$ (2.051(1) Å).[6]

Table S2. Selected interatomic distances [Å] and angles [°] for compound **[L^{e}NiS]$_2$**.

Bond distances (Å)		Bond angles [°]	
S(2)-S(1)	2.051(6)	Ni(1)-S(2)-Ni(2)	104.7(2)
S(2)-Ni(1)	2.180(6)	Ni(2)-S(1)-Ni(1)	104.2(2)
S(2)-Ni(2)	2.191(6)	C(2)-N(1)-Ni(2)	124.8(6)
S(1)-Ni(2)	2.186(6)	C(4)-N(2)-Ni(2)	124.5(7)
S(1)-Ni(1)	2.198(6)	C(7)-N(3)-Ni(1)	122.1(7)
N(1)-C(2)	1.324(11)	N(2)-C(4)-C(3)	123.5(9)
N(1)-Ni(2)	1.867(9)	N(1)-C(2)-C(3)	124.6(8)
N(2)-C(4)	1.337(13)	N(3)-Ni(1)-N(4)	95.1(4)
N(2)-Ni(2)	1.885(8)	N(3)-Ni(1)-S(2)	157.7(3)
N(3)-C(7)	1.314(12)	N(4)-Ni(1)-S(2)	104.0(3)
N(3)-Ni(1)	1.890(8)	N(3)-Ni(1)-S(1)	103.3(3)
C(4)-C(3)	1.409(15)	N(4)-Ni(1)-S(1)	158.4(3)
N(4)-Ni(1)	1.911(9)	S(2)-Ni(1)-S(1)	55.85(19)
C(2)-C(3)	1.394(13)	N(1)-Ni(2)-N(2)	96.7(4)
		N(1)-Ni(2)-S(1)	104.4(3)
		N(2)-Ni(2)-S(1)	156.1(4)
		N(1)-Ni(2)-S(2)	158.5(3)
		N(2)-Ni(2)-S(2)	101.4(3)
		S(1)-Ni(2)-S(2)	55.86(19)

Table S3. Crystal data and structure refinement for **[L^eNiSe]₂** (CCDC: 2334185).

Empirical formula	$C_{50}H_{66}N_4Ni_2Se_2$
Formula weight	998.40
Temperature	150.15 K
Wavelength	1.54184 Å
Crystal system	Monoclinic
Space group	*P* 1 21/*c* 1
Unit cell dimensions	a = 23.4504(3) Å, α = 90°.
	b = 12.02730(10) Å, β = 95.8410(10)°
	c = 16.7151(2) Å, γ = 90°.
Volume	4689.93(9) $Å^3$
Z	4
Density (calculated)	1.414 Mg/m^3
Absorption coefficient	3.049 mm^{-1}
F(000)	2072
Crystal size	0.47 x 0.39 x 0.34 mm^3
Theta range for data collection	4.135 to 72.617°.
Index ranges	-28<=*h*<=22, -14<=*k*<=14, -0<=*l*<=19
Reflections collected	18259
Independent reflections	9079 [R(int) = 0.0229]
Completeness to theta = 67.684°	99.8%
Absorption correction	Semi-empirical from equivalents
Max. and min. transmission	1.00000 and 0.06469
Refinement method	Full-matrix least-squares on F^2
Data / restraints / parameters	9079 / 40 / 610
Goodness-of-fit on F^2	1.021
Final R indices [I>2sigma(I)]	R_1 = 0.0335, wR_2 = 0.0872
R indices (all data)	R_1 = 0.0387, wR_2 = 0.0905
Extinction coefficient	0.00036(4)
Largest diff. peak and hole	0.556 and -0.596 e.$Å^{-3}$

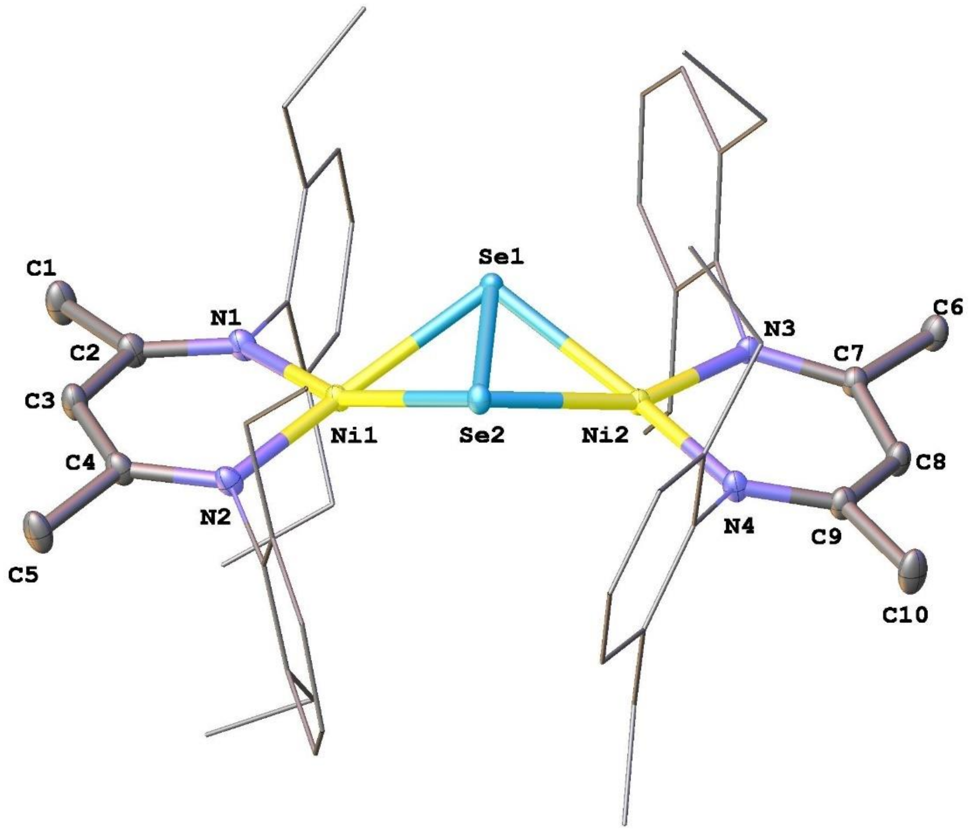

Figure S8. Molecular structure of compound **[L^{e}NiSe]$_2$** (The Ni_2Se_2 moiety is disordered over two orientations with an occupancy ratio of 0.93:0.07. Only one set is depicted). Thermal ellipsoids are drawn at a 30% probability level. H atoms are omitted for clarity. Selected bond lengths (Å) and angles (°): Se1−Se2 2.3192(3), Ni1−Se1 2.304(1), Ni1−Se2 2.321(1), Ni2−Se1 2.3215(9), Ni2−Se2 2.3159(9), Ni1−Se1−Ni2 103.15(4), Ni1−Se2−Ni2 102.81(3), dihedral angle between the two $NiSe_2$ planes: 129.38°. As expected, the Se−Se distance in **[L^{e}NiSe]$_2$** (2.3192(3) Å) is very close to the corresponding distance observed in [[L^{D}NiSe]$_2$ (2.330(4) Å).[7]

Table S4. Selected interatomic distances [Å] and angles [°] for compound **[L^{e}NiSe]$_2$**.

Bond distances (Å)		Bond angles [°]	
Se(2)-Ni(2)	2.3153(11)	Ni(2)-Se(2)-Ni(1)	102.79(3)
Se(2)-Se(1)	2.3195(3)	Ni(1)-Se(1)-Ni(2)	103.13(4)
Se(2)-Ni(1)	2.3207(11)	Se(2)-Se(1)-Ni(2)	59.86(3)
Se(1)-Ni(1)	2.3041(11)	N(4)-Ni(2)-N(3)	96.19(9)
Se(1)-Ni(2)	2.3211(10)	N(4)-Ni(2)-Se(2)	101.48(7)
Ni(2)-N(4)	1.879(2)	N(3)-Ni(2)-Se(2)	158.39(8)
Ni(2)-N(3)	1.882(2)	N(4)-Ni(2)-Se(1)	159.31(8)
Ni(1)-N(2)	1.886(2)	N(3)-Ni(2)-Se(1)	100.20(7)
Ni(1)-N(1)	1.898(2)	Se(2)-Ni(2)-Se(1)	60.04(3)
N(3)-C(7)	1.332(3)	N(2)-Ni(1)-N(1)	96.55(9)
N(4)-C(9)	1.333(3)	N(2)-Ni(1)-Se(1)	157.88(8)
N(2)-C(4)	1.326(3)	N(1)-Ni(1)-Se(1)	101.26(8)
N(1)-C(2)	1.327(3)	N(2)-Ni(1)-Se(2)	99.80(8)
C(4)-C(3)	1.396(3)	N(1)-Ni(1)-Se(2)	159.40(9)
C(2)-C(3)	1.396(3)	Se(1)-Ni(1)-Se(2)	60.20(3)
		C(7)-N(3)-Ni(2)	125.46(15)
		C(9)-N(4)-Ni(2)	124.69(15)
		C(4)-N(2)-Ni(1)	123.87(16)
		C(2)-N(1)-Ni(1)	123.05(16)

Table S5. Crystal data and structure refinement for **[L^e^NiTe]$_2$** (CCDC: 2334186).

Empirical formula	$C_{50}H_{66}N_4Ni_2Te_2$
Formula weight	1095.68
Temperature	150.15 K
Wavelength	1.54184 Å
Crystal system	Monoclinic
Space group	*P* 1 2/*n* 1
Unit cell dimensions	a = 24.6058(5) Å, α = 90°
	b = 8.22660(10) Å, β = 114.245(2)°
	c = 26.0317(6) Å, γ = 90°
Volume	4804.62(17) Å^3
Z	4
Density (calculated)	1.515 Mg/m^3
Absorption coefficient	10.636 mm^{-1}
F(000)	2216
Crystal size	0.15 x 0.06 x 0.05 mm^3
Theta range for data collection	3.219 to 72.682°.
Index ranges	$-28<=h<=30$, $-10<=k<=10$, $-31<=l<=32$
Reflections collected	35223
Independent reflections	9431 [R(int) = 0.0460]
Completeness to theta = 67.684°	99.9%
Absorption correction	Semi-empirical from equivalents
Max. and min. transmission	1.00000 and 0.06113
Refinement method	Full-matrix least-squares on F^2
Data / restraints / parameters	9431 / 37 / 572
Goodness-of-fit on F^2	1.059
Final R indices [I>2sigma(I)]	R_1 = 0.0426, wR_2 = 0.1043
R indices (all data)	R_1 = 0.0544, wR_2 = 0.1120
Extinction coefficient	n/a
Largest diff. peak and hole	1.166 and -0.738 e.Å^{-3}

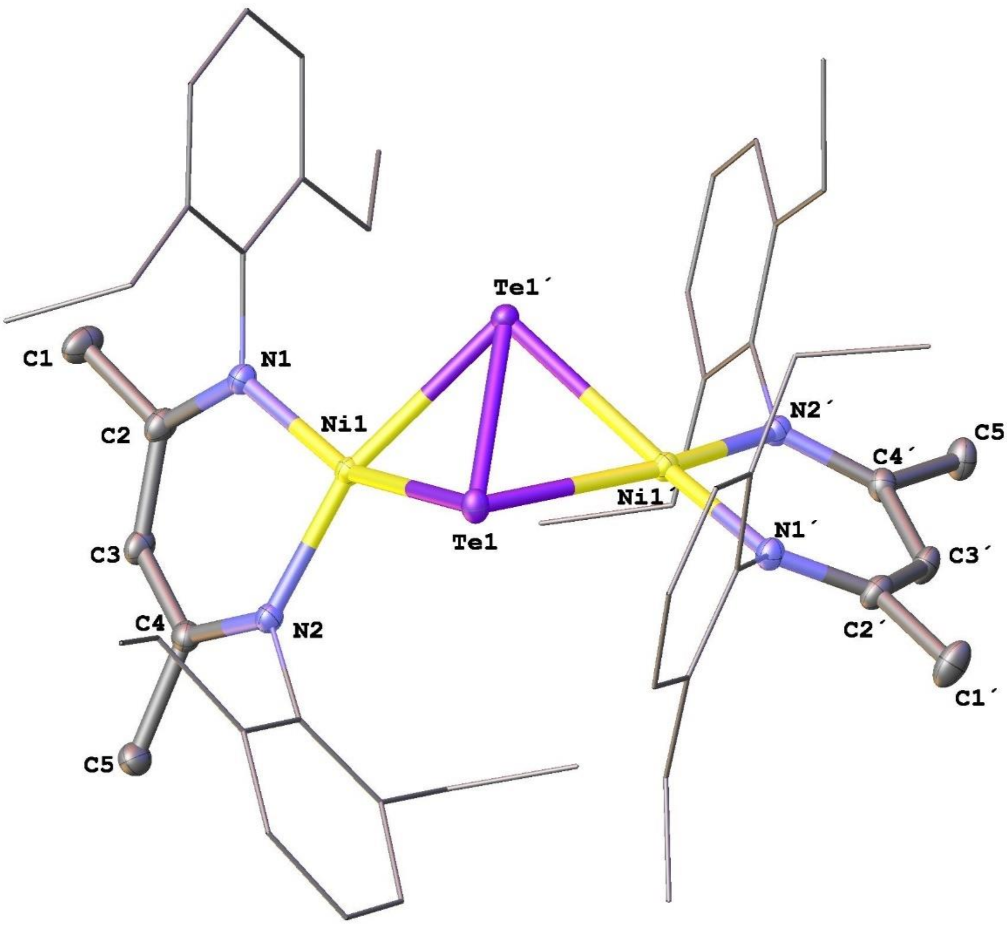

Figure S9. Molecular structure of compound **[L^{e}NiTe]$_2$** (Two independent molecules of **[L^{e}NiTe]$_2$** present in the asymmetric unit. Only one is depicted). Thermal ellipsoids are drawn at a 30% probability level. H atoms are omitted for clarity. Symmetry transformations are used to generate equivalent atoms with "´": -x+1/2,y,-z+1/2. Selected bond lengths (Å) and angles (°): Te1−Te1´ 2.6739(5), Ni1−Te1 2.4953(7), Ni1−Te1´ 2.5042(8), Ni1−Te1−Ni1´ 94.17(3), dihedral angle between the two $NiTe_2$ planes: 122.68°. Although two independent molecules of **[L^{e}NiTe]$_2$** are present in the asymmetric unit, the Te−Te distances of 2.6739(5) and 2.6685(6) Å are almost identical and very close to the corresponding value in [L^{D}NiTe]$_2$ (2.6994(3) Å).[7]

Table S6. Selected interatomic distances [Å] and angles [°] for the dianion of compound **[L[e]NiTe]₂**.

**Bond distances (Å)**			**Bond angles [°]**		
	**Mol. 1**	**Mol. 2**		**Mol. 1**	**Mol. 2**
Te(1)-Te(1´)	2.6739(5)	2.6694(6)	Ni(1)-Te(1)-Ni(1´)	94.16(3)	97.29(3)
Ni(1)-Te(1)	2.4951(7)	2.5029(9)	N(1)-Ni(1)-Te(1)	99.06(12)	101.88(12)
Ni(1)-Te(1´)	2.5043(8)	2.4972(9)	N(2)-Ni(1)-Te(1´)	162.84(11)	100.27(14)
Ni(1)-N(1)	1.901(3)	1.900(4)	Te(1)-Ni(1)-Te(1´)	64.67(2)	64.54(2)
Ni(1)-N(2)	1.891(4)	1.890(4)	N(1)-Ni(1)-N(2)	96.57(15)	95.20(19)
N(1)-C(2)	1.329(6)	1.323(7)	C(2)-N(1)-Ni(1)	123.6(3)	124.3(4)
C(2)-C(3)	1.390(7)	1.405(7)	N(1)-C(2)-C(3)	123.5(4)	122.6(4)
C(3)-C(4)	1.394(6)	1.423(8)	C(2)-C(3)-C(4)	127.1(4)	128.3(5)
C(4)-N(2)	1.338(6)	1.300(7)	C(3)-C(4)-N(2)	123.6(4)	119.9(5)
			C(4)-N(2)-Ni(1)	123.3(3)	127.7(4)

Characterization of the as-synthesized NiE materials

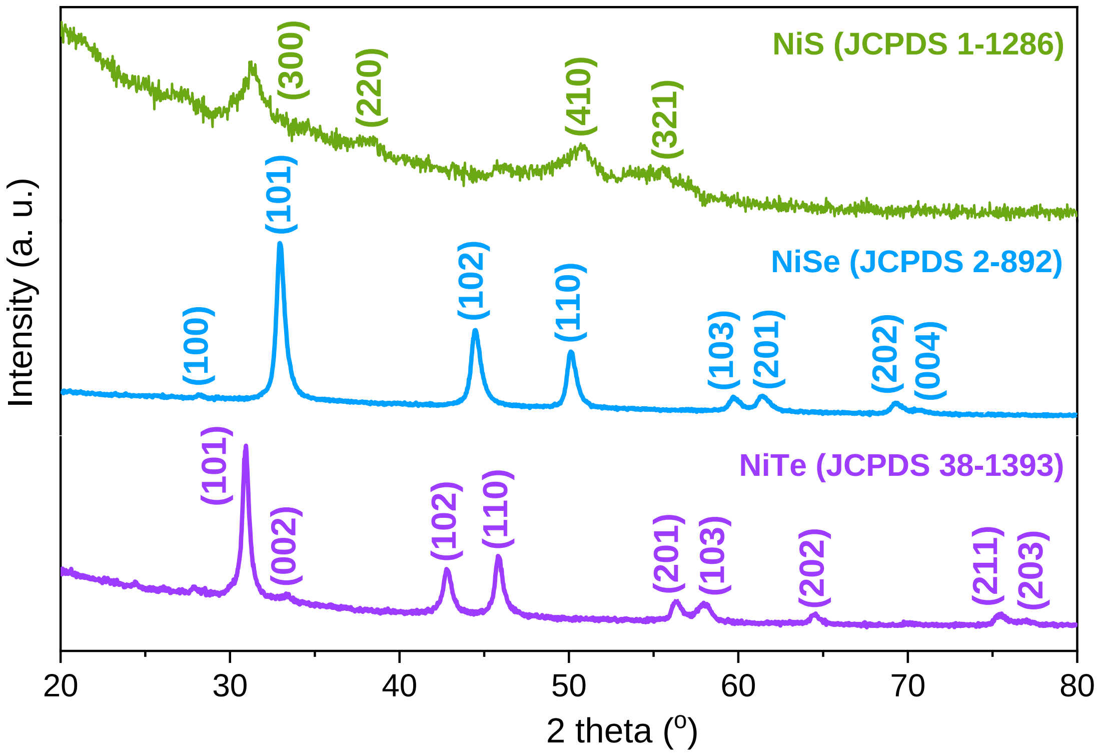

Figure S10. PXRD pattern of as-synthesized NiS, NiSe and NiTe.

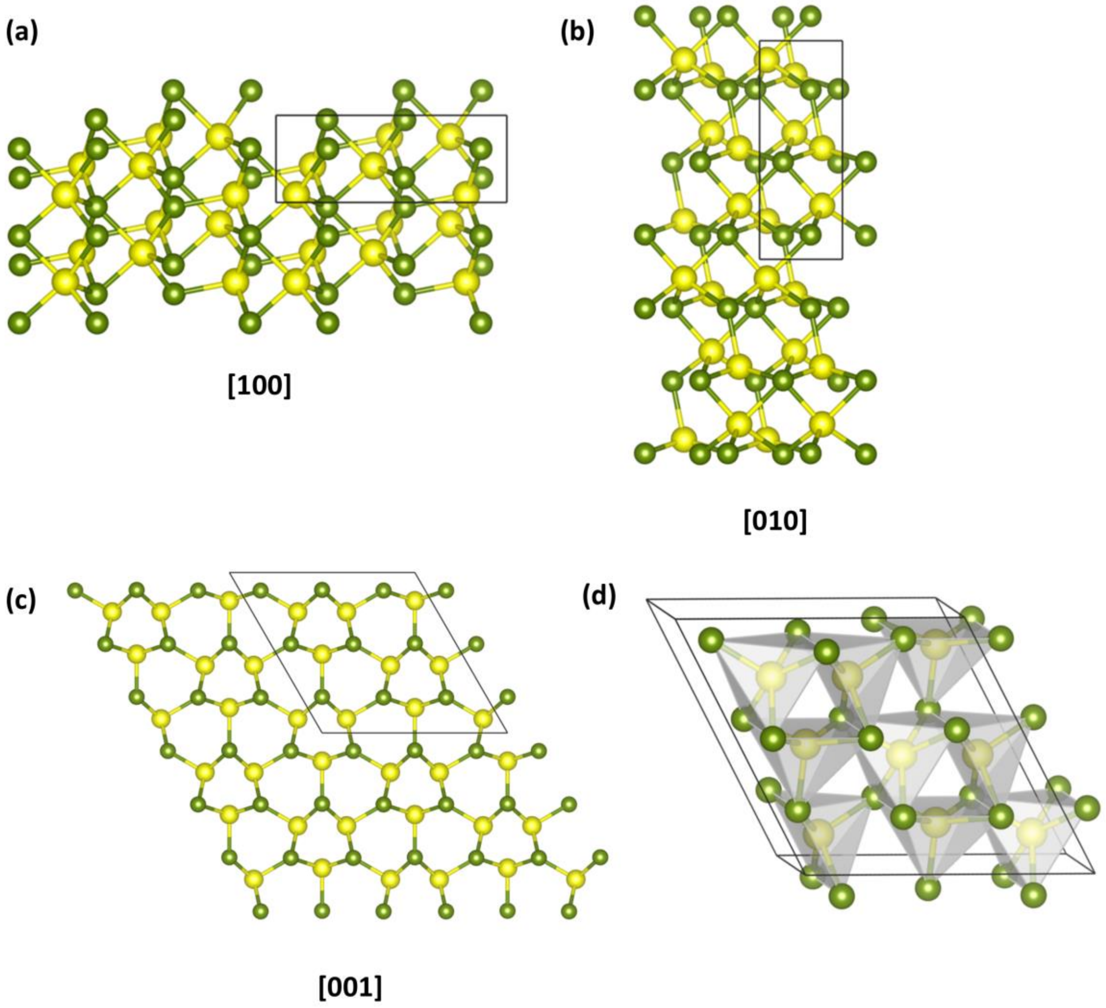

Figure S11. (a) The crystal structure of NiS (yellow: Ni, green: S) on crystallographic (a) [100], (b) [010], and (c) [001] direction. (d) A unit cell (black lines) of the NiS crystal structure. The NiS crystallizes in the rhombohedral space group *R*3*m* (Nr. 160), with unit parameters $a = b = 9.61900$ Å, $c = 3.14990$ Å; $\alpha = \beta = 90°$, $\gamma = 120°$; $V = 252.39$ Å^3; $Z = 9$. NiS forms the pure mineral millerite, accessible at low temperatures, and it consists of Ni_3S_9 clusters made of three edge-sharing NiS_5 distorted tetragonal pyramids.[8] Here, Ni is coordinated to five S atoms, forming a distorted tetragonal pyramid. These S polyhedra are edge-sharing across all four equatorial edges. The Ni atoms of three edge-sharing form the corners of an equilateral triangle. Therefore, the NiS framework consists of Ni_3S_9 clusters interconnected through S atoms.[8]

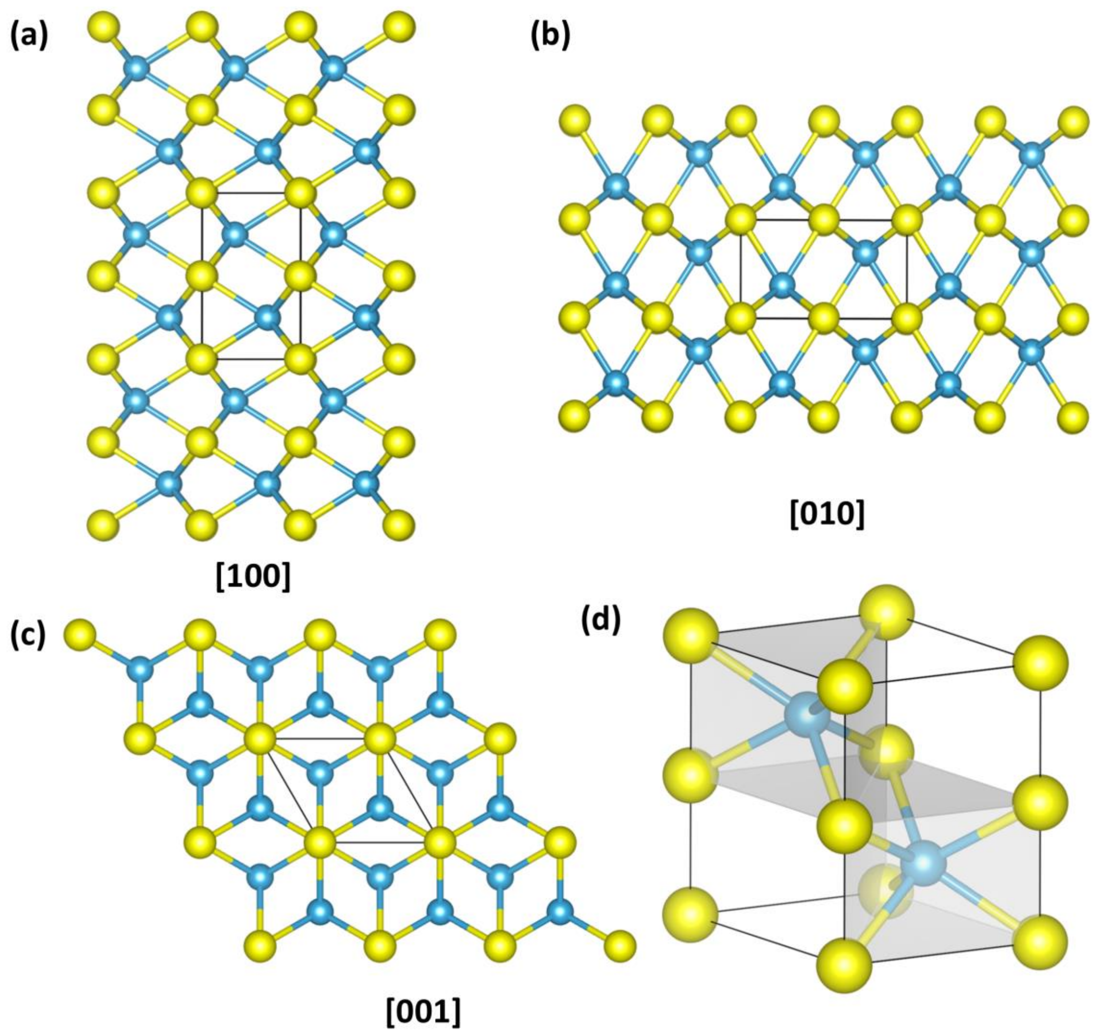

Figure S12. The crystal structure of NiSe (yellow: Ni, blue: Se) on crystallographic (a) [100], (b) [010], and (c) [001] direction. (d) A unit cell (black lines) of the NiSe crystal structure. The NiSe crystallizes in the hexagonal space group $P6_3/mmc$ (Nr. 194), with unit parameters $a = b = 3.660$ Å, $c = 5.330$ Å; $\alpha = \beta = 90°$, $\gamma = 120°$; $V = 61.83$ Å^3; $Z = 2$. It features edge-sharing Ni_6Se trigonal prisms arranged in a hexagonal close-packed structure.[9] Here, Se forms a hexagonal close-packed structure, wherein Ni occupies the octahedral sites. The unit cell (grey lines) consists of two edge-sharing Ni_6Se trigonal prims, where Se occupies the center. Moreover, half of the lattice volume consists of face-sharing tetrahedral voids.[9]

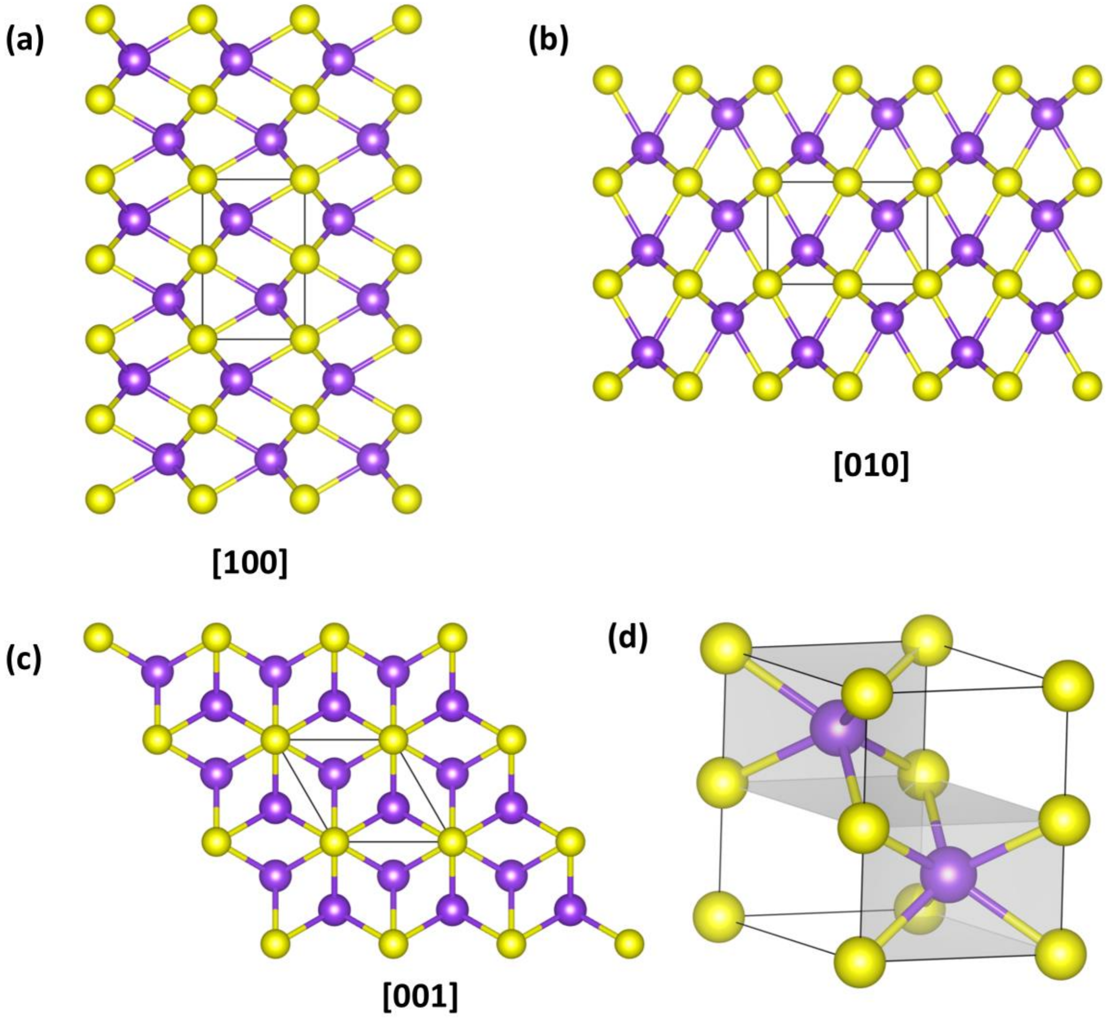

Figure S13. The crystal structure of as-synthesized NiTe (yellow: Ni, purple: Te) on crystallographic (a) [100], (b) [010], and (c) [001] direction. (d) A unit cell (black lines) of the NiTe crystal structure. The NiTe crystallizes in the hexagonal space group $P6_3/mmc$ (Nr. 194), with unit parameters $a = b =$ 3.955 Å, c = 5.359 Å; $\alpha = \beta = 90°$, $\gamma = 120°$; V = 72.59 Å^3; Z = 2. NiTe forms the pure mineral imgreite and has the same structure as NiSe.

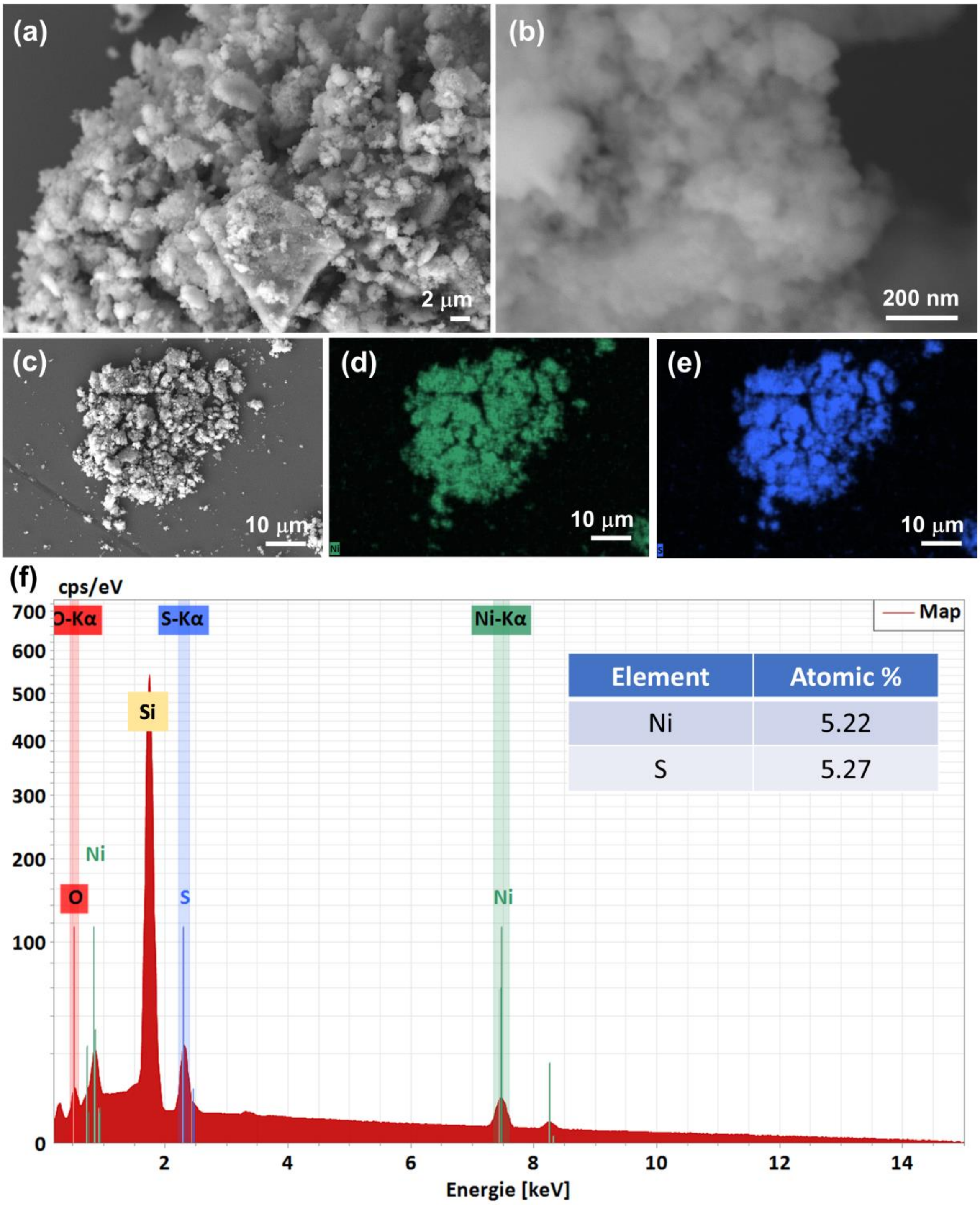

Figure S14. (a, b) SEM images of NiS nanostructure at different magnifications showing the presence of agglomeration. (c, d, e) Elemental mapping displays a homogeneous distribution of Ni (green) and S (blue) in NiS. (f) Atomic percentages from the SEM-EDX spectrum confirmed the Ni:S ratio to be 1:1.01 in the sample. The Si peak arises due to the Si wafer substrate. The estimated oxygen content is <2%.

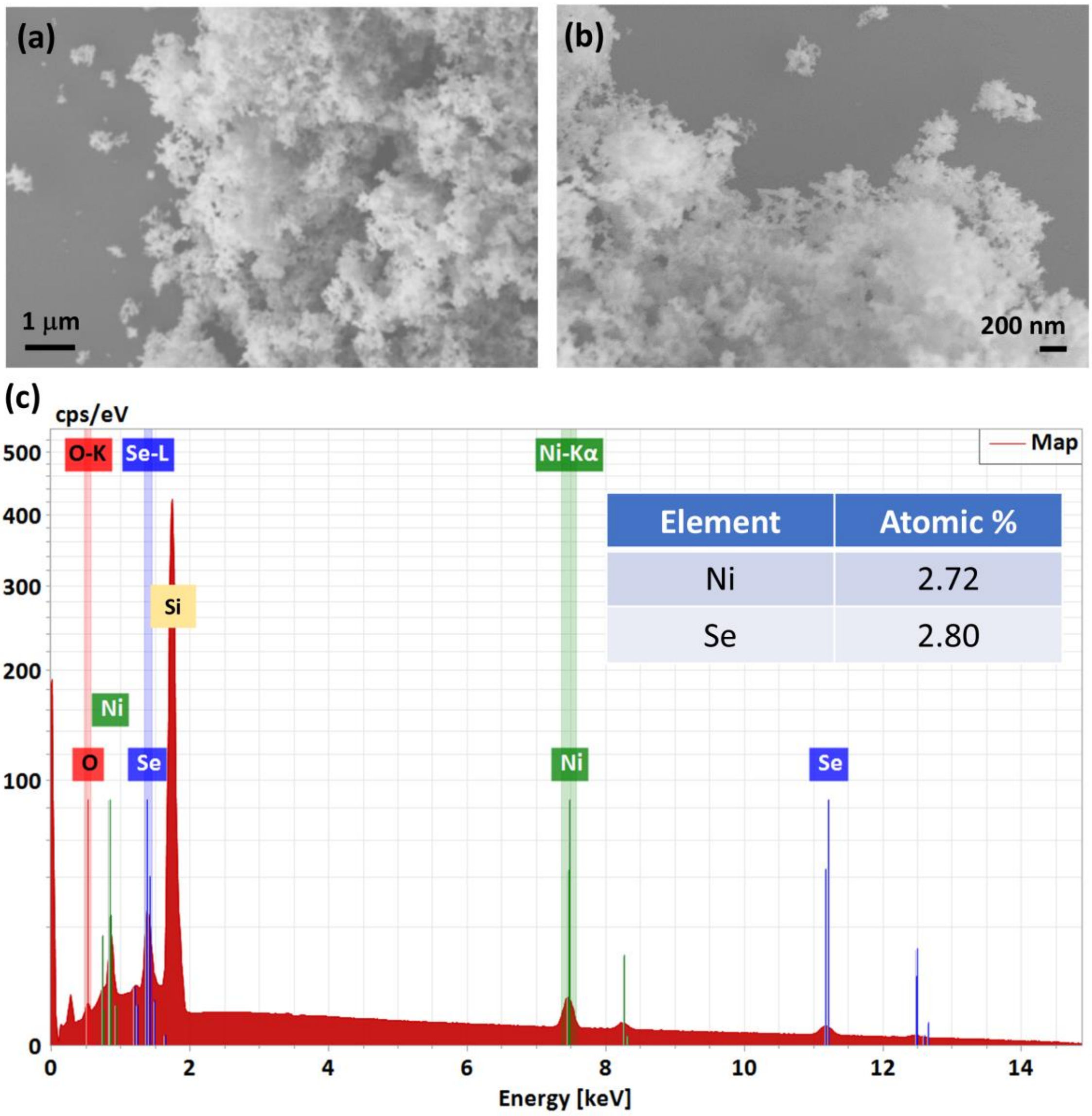

Figure S15. (a, b) SEM images of NiSe at different magnifications showing agglomerated particles. (c) Atomic percentages from the SEM-EDX spectrum confirms the Ni:Se ratio to be 1:1.03 in the sample. The Si peak arises due to the Si wafer substrate. The estimated oxygen content is <2%.

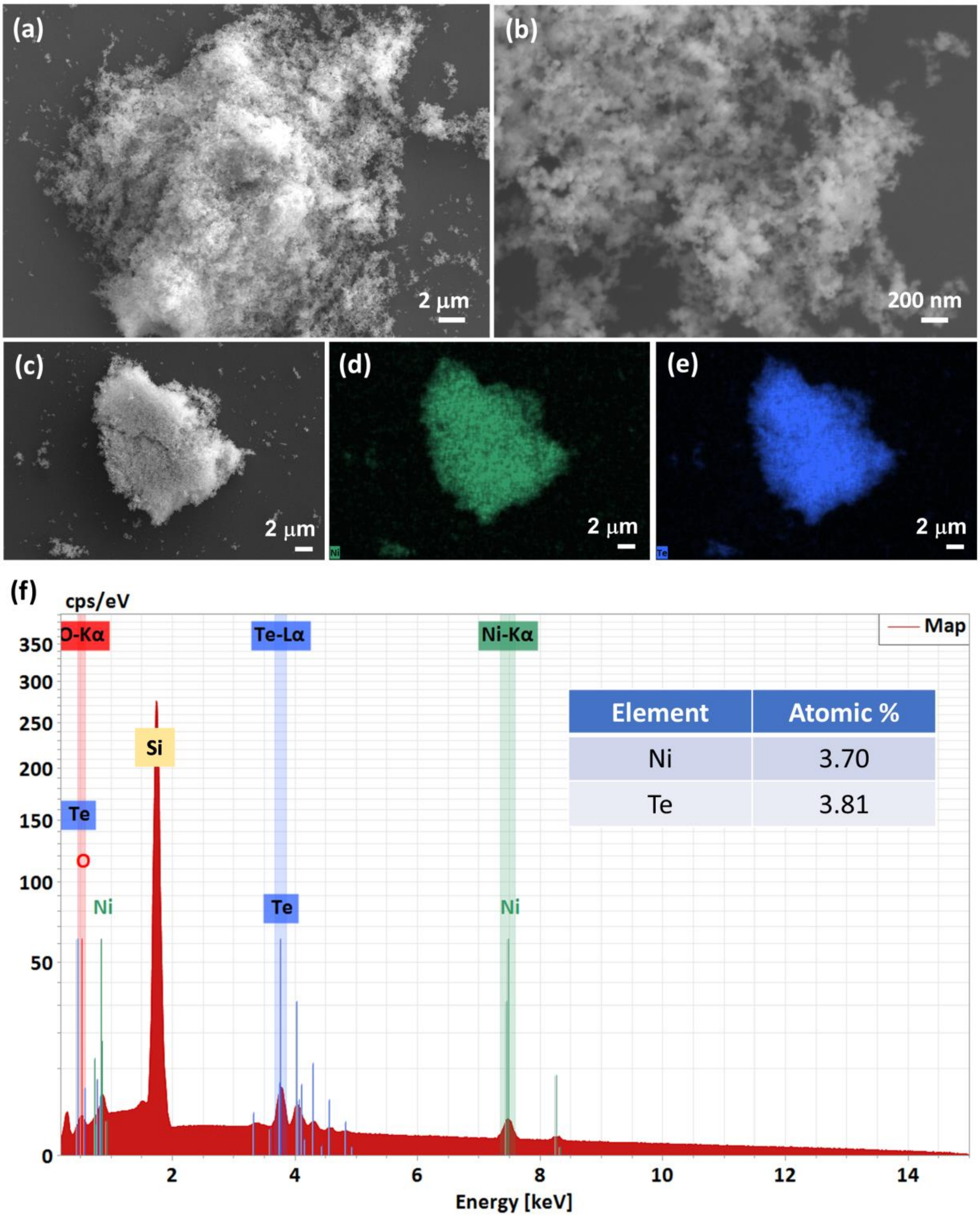

Figure S16. (a, b) SEM images of NiTe nanostructure at different magnifications showing the presence of agglomeration. (c, d, e) Elemental mapping displays a homogeneous distribution of Ni (green) and Te (blue) in NiTe. (f) Atomic percentages from the SEM-EDX spectrum confirms the Ni:Te ratio to be 1:1.03 in the sample. The Si peak arises due to the Si wafer substrate. The estimated oxygen content is <2%.

Table S7. Ni:E ratio in as-synthesized NiS, NiSe, and NiTe powders, as-deposited on FTO and after 24 h CP at 10 mA/cm^2 determined by ICP-OES. The average value of three independent measurements is shown for each material and method.

NiE (E = S, Se, Te)	Ni:E ratio
NiS	1:0.99
NiSe	1:1.02
NiTe	1:1.02
NiS/FTO	1:0.98
NiSe/FTO	1:1.01
NiTe/FTO	1:1.02
NiS-OER	1:0.02
NiSe-OER	1:0.01
NiTe-OER	1:0.65

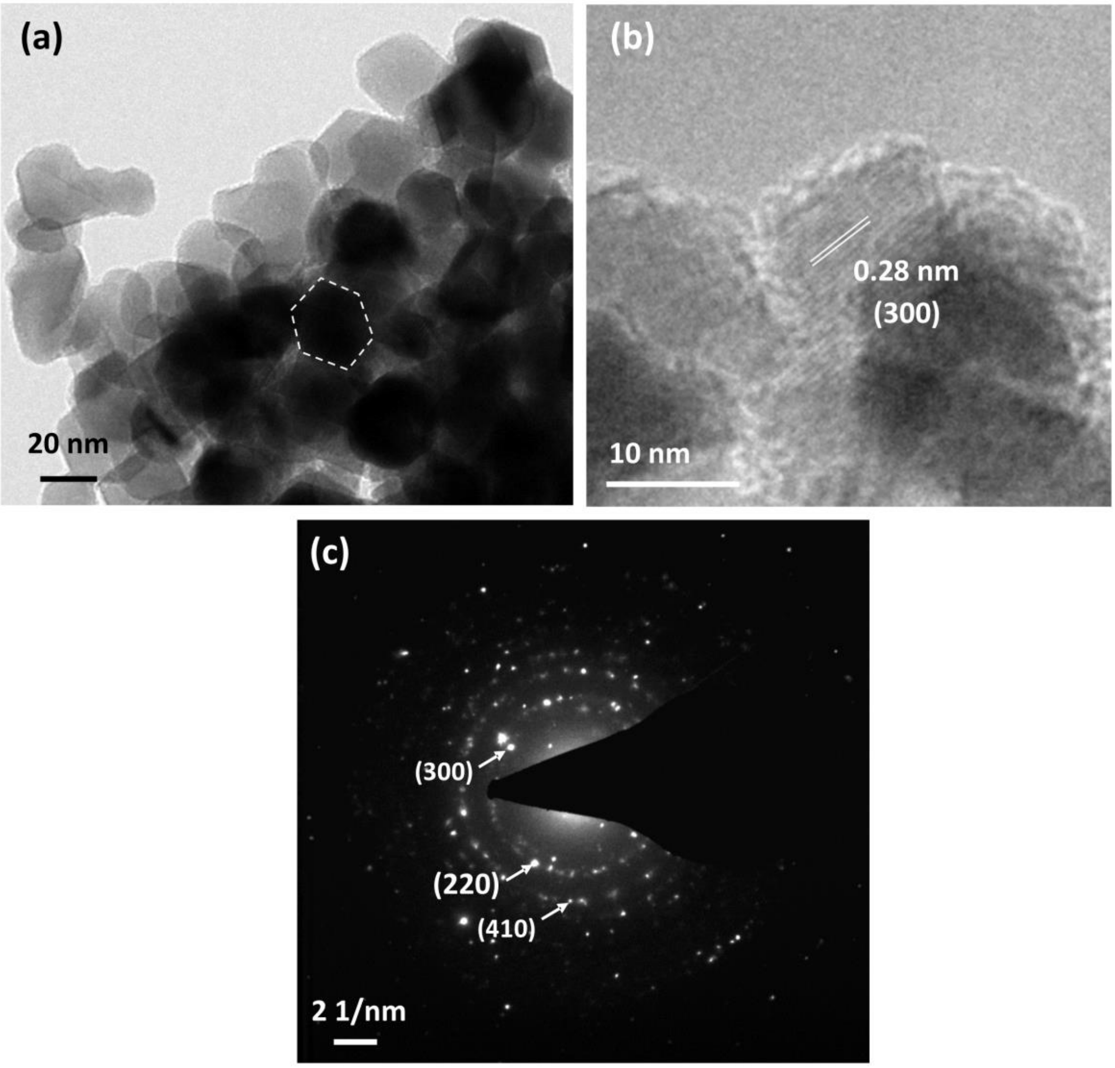

Figure S17. (a) TEM image of NiS revealing distorted hexagonal-shaped particles, and the corresponding (b) HR-TEM showing a lattice fringe spacing of 0.28 nm for the (300) plane. (c) SAED showing a ring pattern typical for polycrystalline materials, where (300), (220) and (410) planes of NiS were identified (JCPDS 1-1286).

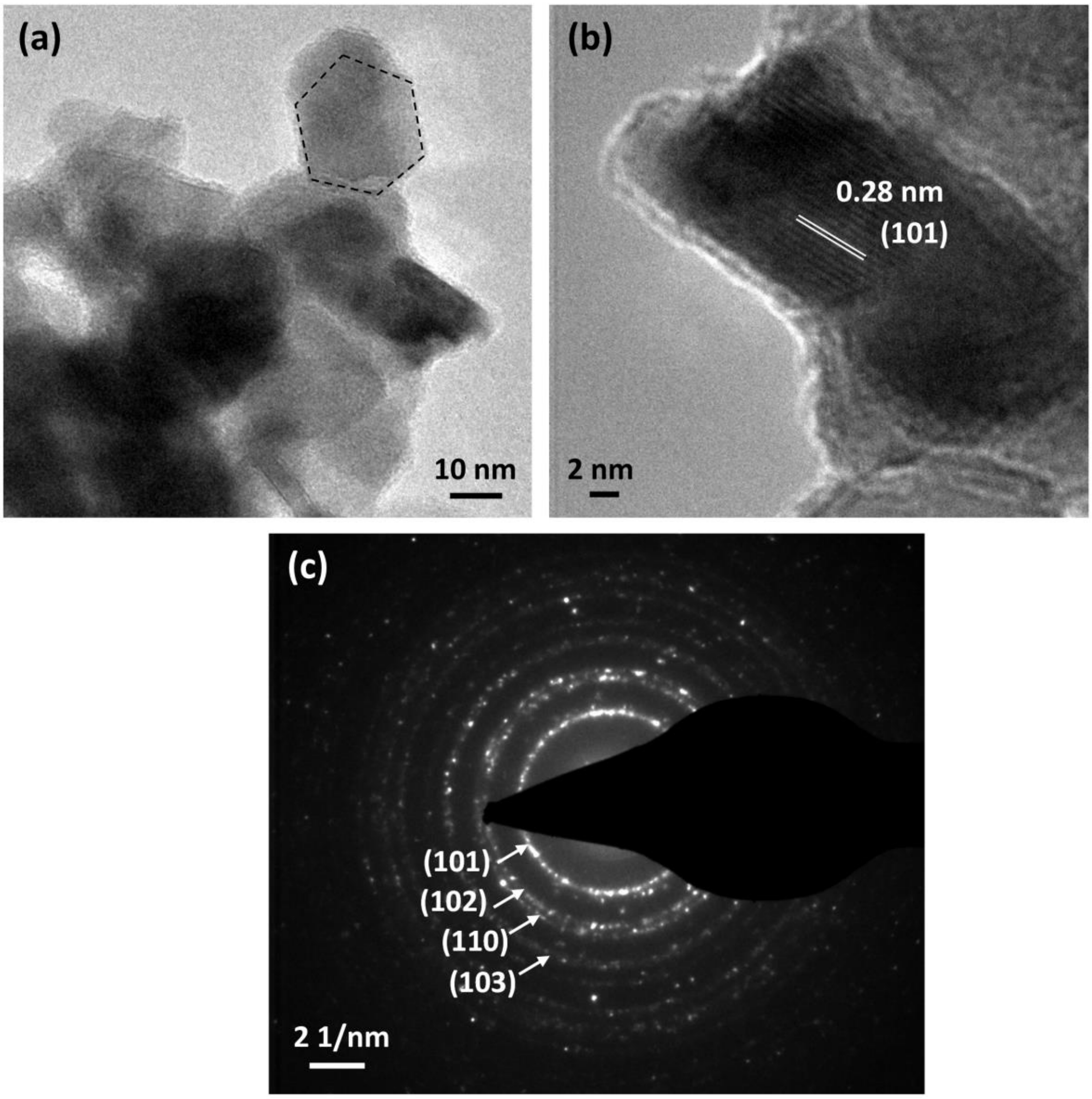

Figure S18. (a) TEM image of NiTe revealing distorted shaped-shaped particles, and the corresponding (b) HR-TEM showing a lattice fringe spacing of 0.28 nm for the (101) plane. (c) SAED shows a ring pattern typical for polycrystalline materials, where (101), (102), (110) and (103) planes of NiTe were identified (JCPDS 38-1393).

Characterization of as-deposited NiSe on FTO and NF

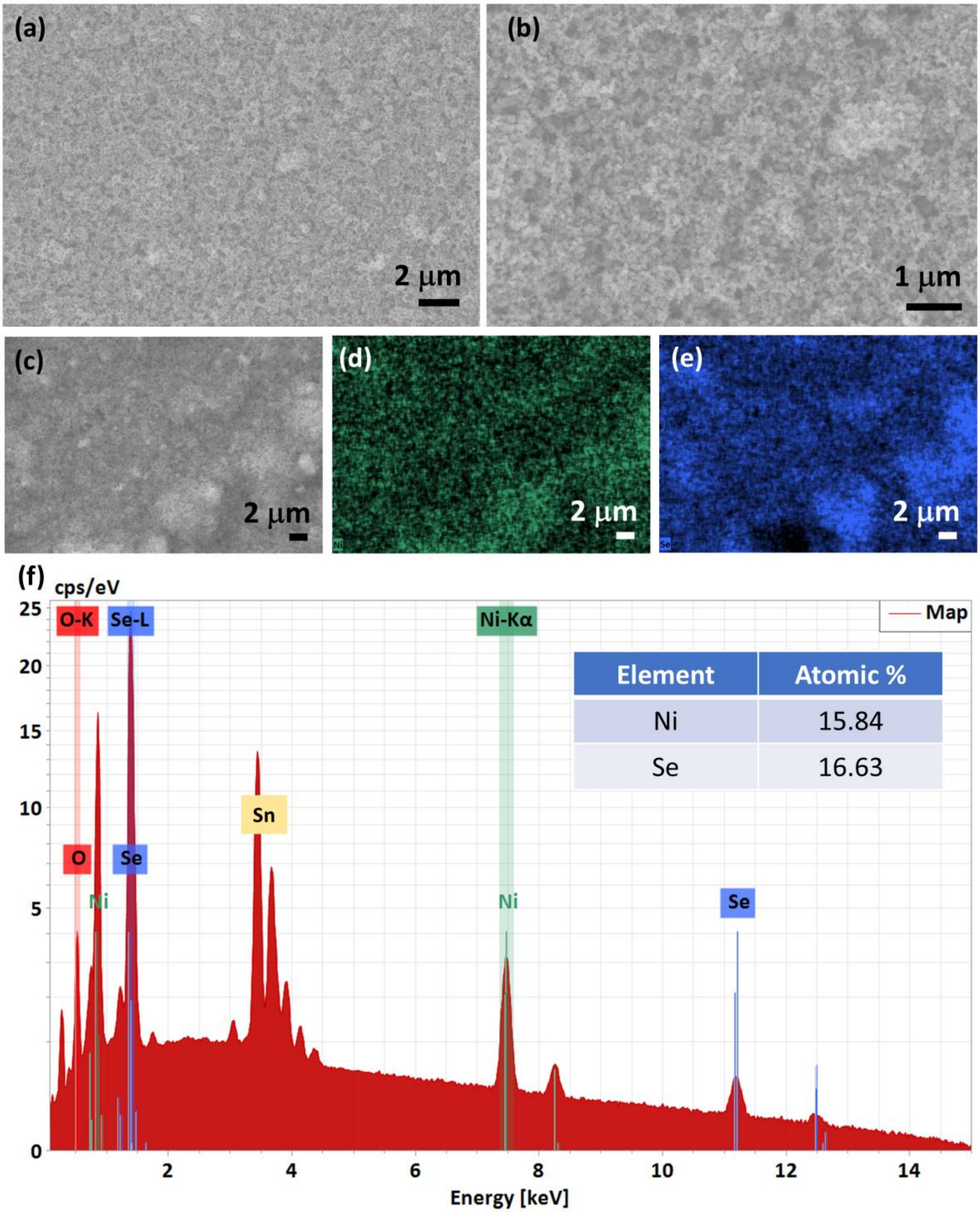

Figure S19. (a, b) SEM images of as-deposited NiSe film on FTO at different magnifications showing a homogeneous deposition of the sample throughout the FTO substrate. (c, d, e) Elemental mapping shows a homogeneous distribution of Ni (green) and Se (blue) in the as-deposited sample. (f) Atomic percentages from the SEM-EDX spectrum confirms the Ni:Se ratio to be 1:1.04, which is similar to the as-synthesized NiSe. The large O signal, as well as the Sn signal arise from the FTO glass substrate electrode.

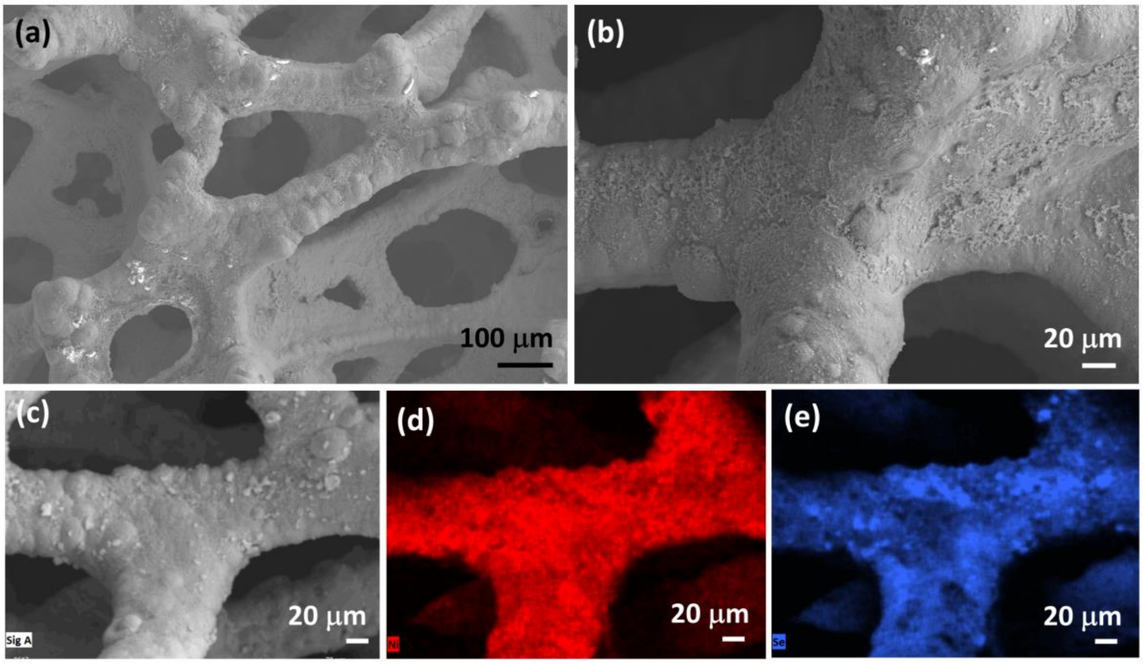

Figure S20. (a, b) SEM images of as-deposited NiSe film on NF at different magnifications. (c, d, e) Elemental mapping shows the homogeneous distribution of Ni (red) and Se (blue) in the as-deposited sample. These findings closely resemble the characteristics of the as-synthesized NiSe.

Electrochemical measurements for OER

Table S8. OER activity comparison of the Ni Chalcogenides in this work with other established Ni chalcogenide-based materials at 10 mA/cm^2 in 1 M KOH.

Material	Substrate	η_{10} (mV)	Reference
NiSe	*NF*	*247 ± 2*	*This work*
	FTO	*340 ± 6*	
NiS	*NF*	*310 ± 3*	
	FTO	*378 ± 8*	
NiTe	*NF*	*339 ± 5*	
	FTO	*464 ± 11*	
NiSe	GC RDE	389	10
$NiSe_2$/CoSe	NF	220	11
$CoNi_{0.5}Se$	GC	250	12
$NiSe_2$	GC	299	13
Mo-Ni-Se	NF	397	14
NiSe	Graphene/NM	481	15
$Ni_xFe_{1-x}Se_2$	NF	195	16
NiSe-Polyaniline	NF	300	17
$NiSe_2$	CC	210	18
$Ni_{0.85}Se$–NHCS	GC RDE	353	19
$Ni_{0.5}Fe_{0.5}Se_2$	GC	235	20
$NiSe_2$	NF	235	21
S-substituted Ni_3Se_4	CP	275	22
NiSe	NF	252	23
$NiSe_2$	GC	250	24
$Ni-Ni_3S_2$	NF	310	25
NiS	GC	320	26
CuNiS	NF	337	27
NiFeS	GC	286	28
Ni/NiS/NC	GC	337	29
Meso C-NiFeS	GC	350	30
NiS	FTO	350	31
NiS	NF	255	31
NiFe-OH/Ni_3S_2	NF	268	32
NiTe	GR	679	33
Ni-doped $CoTe_2$	CF	280	34
$NiTe_2$	TM	315	35

Here, GC = Glassy Carbon, NM = Ni Mesh, CC = Carbon Cloth, RDE = Rotating Disk Electrode, CP = Carbon Paper, GR = Graphite Rod, CF = Copper Foam, TM = Ti Mesh

Electrochemical measurements for OER on NF

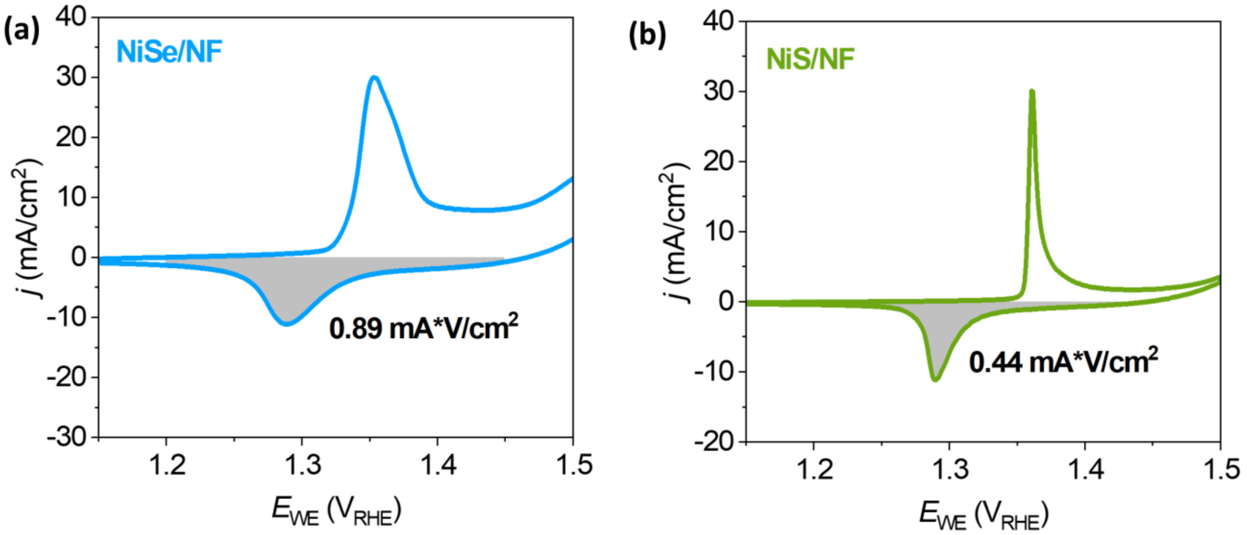

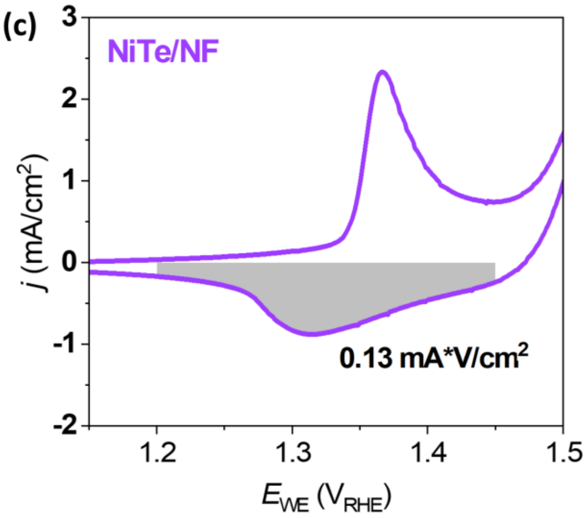

(e)

$$[e^-] = \frac{1}{\text{scan rate} * F} * \int_{V_1}^{V_2} I(E)dE$$

Scan rate = 1 mV/s, F = 96485 sA/mol

Material	$\int_{V_1}^{V_2} I(E)dE$	$[e^-]$ (μmol)
NiSe	0.89 mA*V	9.2
NiS	0.44 mA*V	4.6
NiTe	0.13 mA*V	1.4

Figure S21. CV (scan rate 1 mV/s) of (a) NiSe/NF, (b) NiS/NF, and (c) NiTe/NF measured in 1 M KOH. The materials show a pair of peaks between 1.20 V_{RHE} and 1.45 V_{RHE}, corresponding to $Ni^{II} \rightarrow Ni^{III/IV}$ oxidation in alkaline media.[36–39] (e) Integration of the reduction peak and calculation of the number of moles of electrons transferred during the reduction of the respective catalyst.

Table S9. The fitting parameter of the impedance spectra for NiE/NF. The equivalent circuit is provided in Figure 3b of the manuscript.

Material	R_{ct} (Ω)	R_s (Ω)	Q (F × $s^{(a-1)}$)	a
NiSe/NF	1.9 ± 0.2	0.8 ± 0.1	0.188 ± 0.003	0.909 ± 0.006
NiS/NF	2.8 ± 0.9	0.8 ± 0.1	0.243 ± 0.005	0.850 ± 0.006
NiTe/NF	6.5 ± 1	1.6 ± 0.5	0.114 ± 0.008	0.893 ± 0.004

Here, Ω = Ohms, F = Farad, s = seconds, a = dimensional parameter between 0 and 1.

Table S10. Calculation of TOF of NiE/NF.

Material	j at 1.63 V_{RHE} (mA/cm^2)	TOF (s^{-1})
NiSe/NF	210.6	0.095
NiS/NF	101.5	0.091
NiTe/NF	29.2	0.086

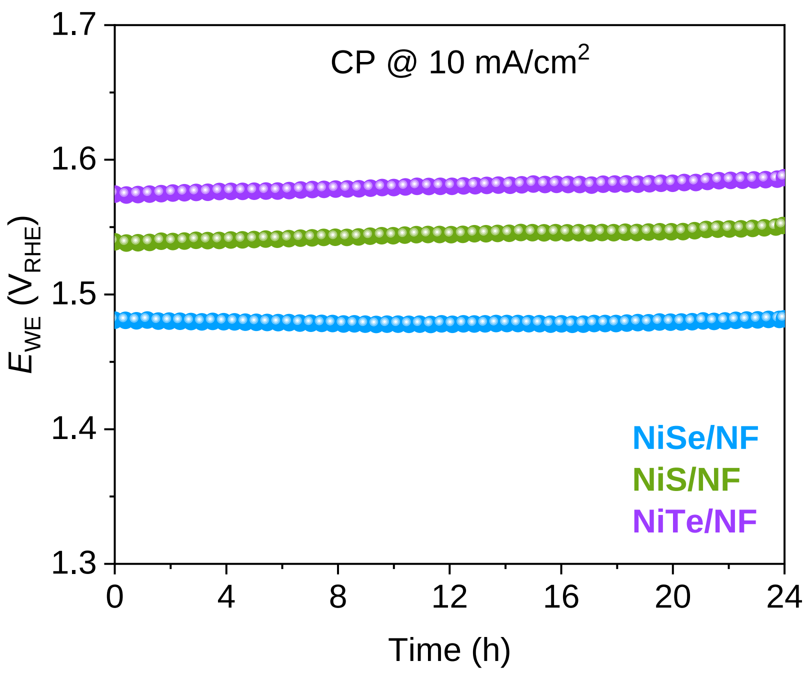

Figure S22. CP at 10 mA/cm^2 for NiE/NF showing stable performances for 24 hours.

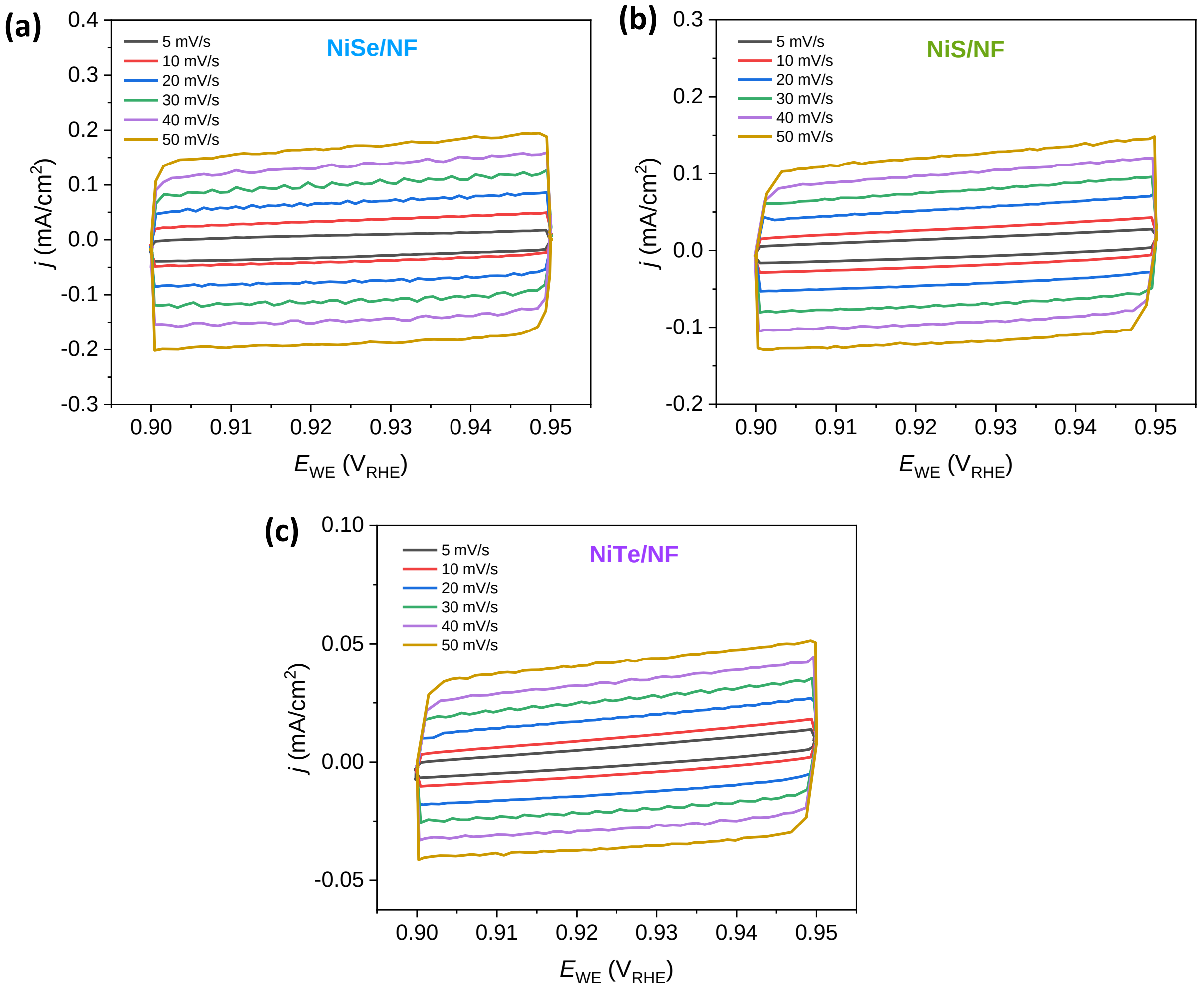

Figure S23. C_{dl} analysis of (a) NiSe/NF, (b) NiS/NF, and (c) NiTe/NF after 24 h CP at 10 mA/cm^2. The CV scans were conducted in a non-Faradaic potential range at different scan rates. Half of the differences in current density variation ($\Delta j = (j_{cathodic}-j_{anodic})/2$) at a potential of 0.925 V_{RHE} were plotted against the scan rate and fitted by linear regression allowing the determination of the C_{dl} (Figure 3d of the manuscript).

Table S11. FE for OER of NiSe/NF material.

Material	j (mA/cm^2)	t (s)	V_{O_2} (mL)	FE (O_2,%)
NiSe/NF	49.3	3600	10.95	97 ± 2

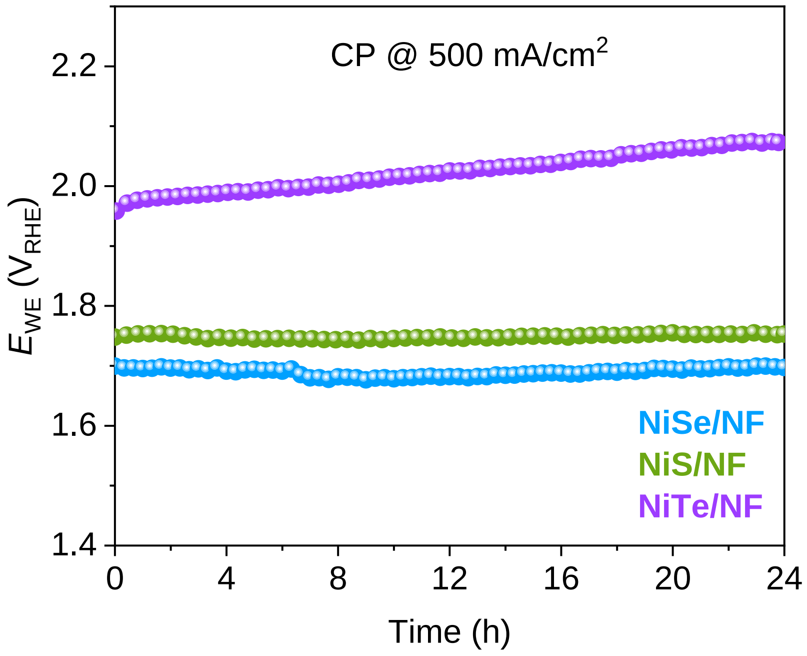

Figure S24. CP at 500 mA/cm^2 for NiE/NF for 24 hours.

Electrochemical measurements for OER on FTO

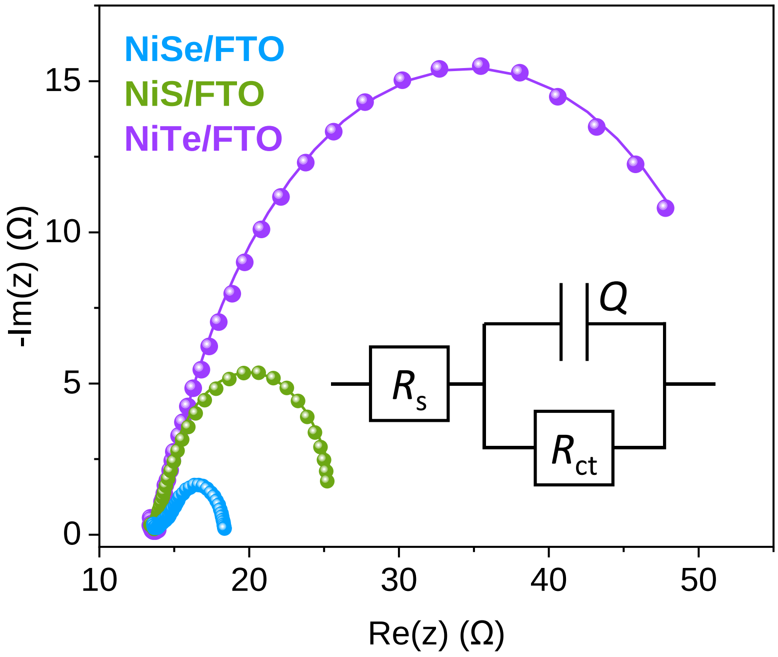

Figure S25. Nyquist plot for NiE/FTO. An equivalent Randles circuit was used during the EIS fitting as shown in the inset. The fitted curve is designated with a linear plot. EIS was recorded at a constant potential of 1.56 V_{RHE}. The fitting parameters are given in Table S12.

Table S12. Fitting parameters of the impedance spectra for NiE/FTO.

Material	R_{ct} (Ω)	R_s (Ω)	Q (F × $s^{(a-1)}$)	a
NiSe/FTO	4 ± 1	14.4 ± 0.2	0.125 ± 0.006	0.844 ± 0.006
NiS/FTO	12 ± 1	13.8 ± 0.4	0.204 ± 0.009	0.903 ± 0.007
NiTe/FTO	41 ± 3	10.2 ± 0.7	0.167 ± 0.004	0.800 ± 0.006

Here, Ω = Ohms, F = Farad, s = seconds, a = dimensional parameter between 0 and 1.

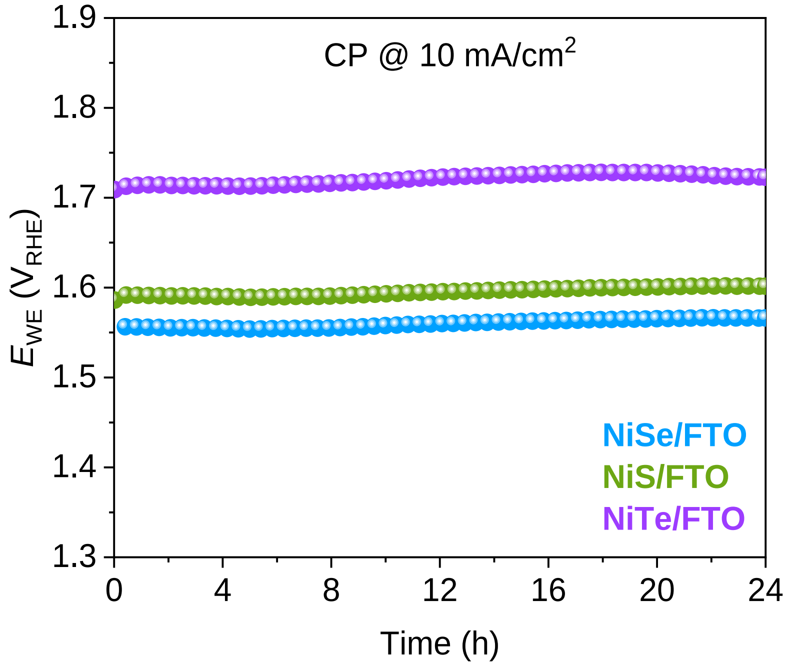

Figure S26. CP at 10 mA/cm^2 for NiE/FTO showing stable performances for 24 hours.

Post-OER Characterization

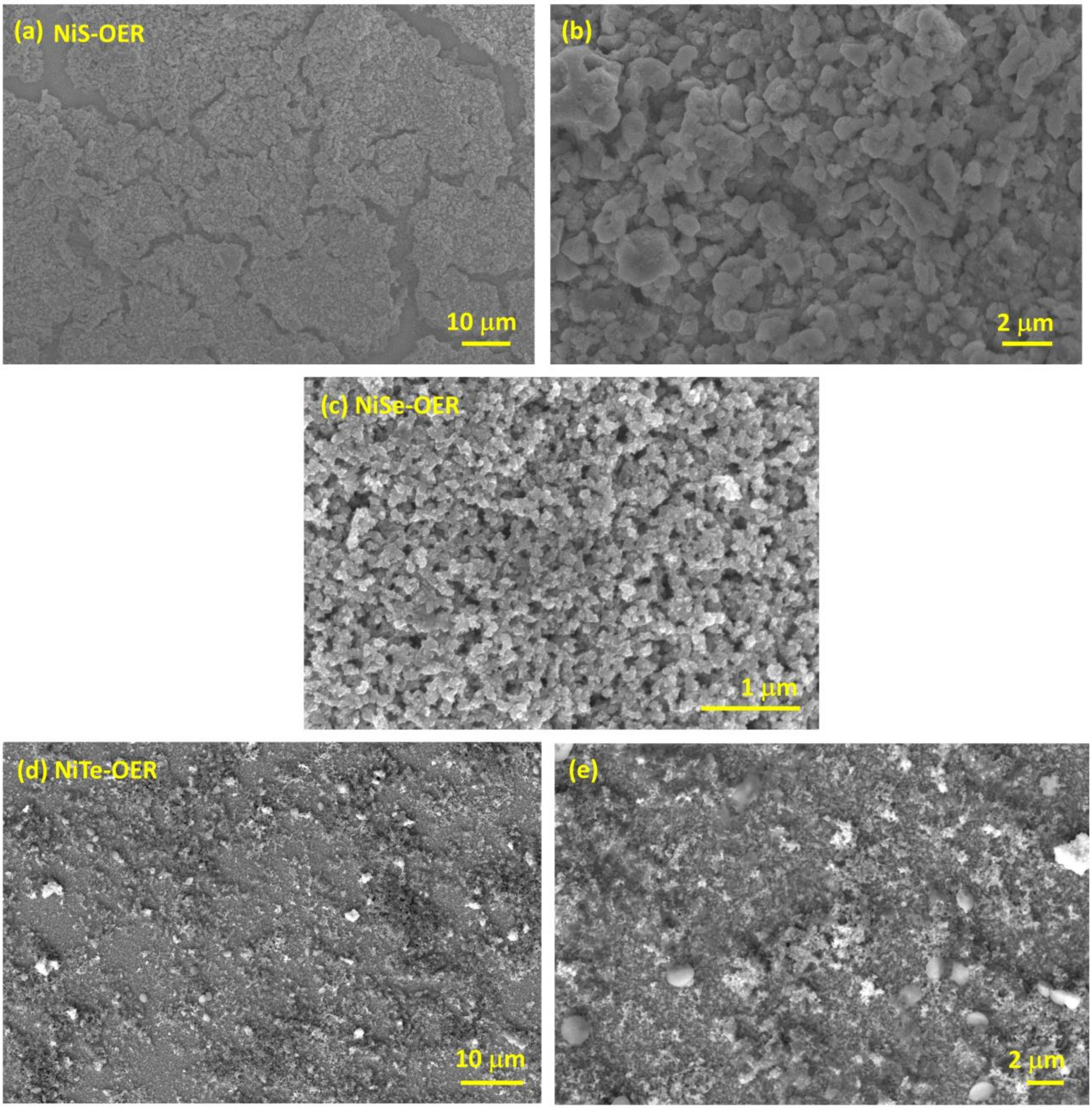

Figure S27. SEM images of NiE/FTO films after 24 h CP at 10 mA/cm^2 at different magnifications. The images of (a, b) NiS/FTO, and (c) NiSe/FTO (also see Figure 4a of the manuscript) reveal that the surface of the films were substantially altered after OER treatment. However, the images of (d, e) NiTe/FTO depict no abrupt change in the films after OER treatment.

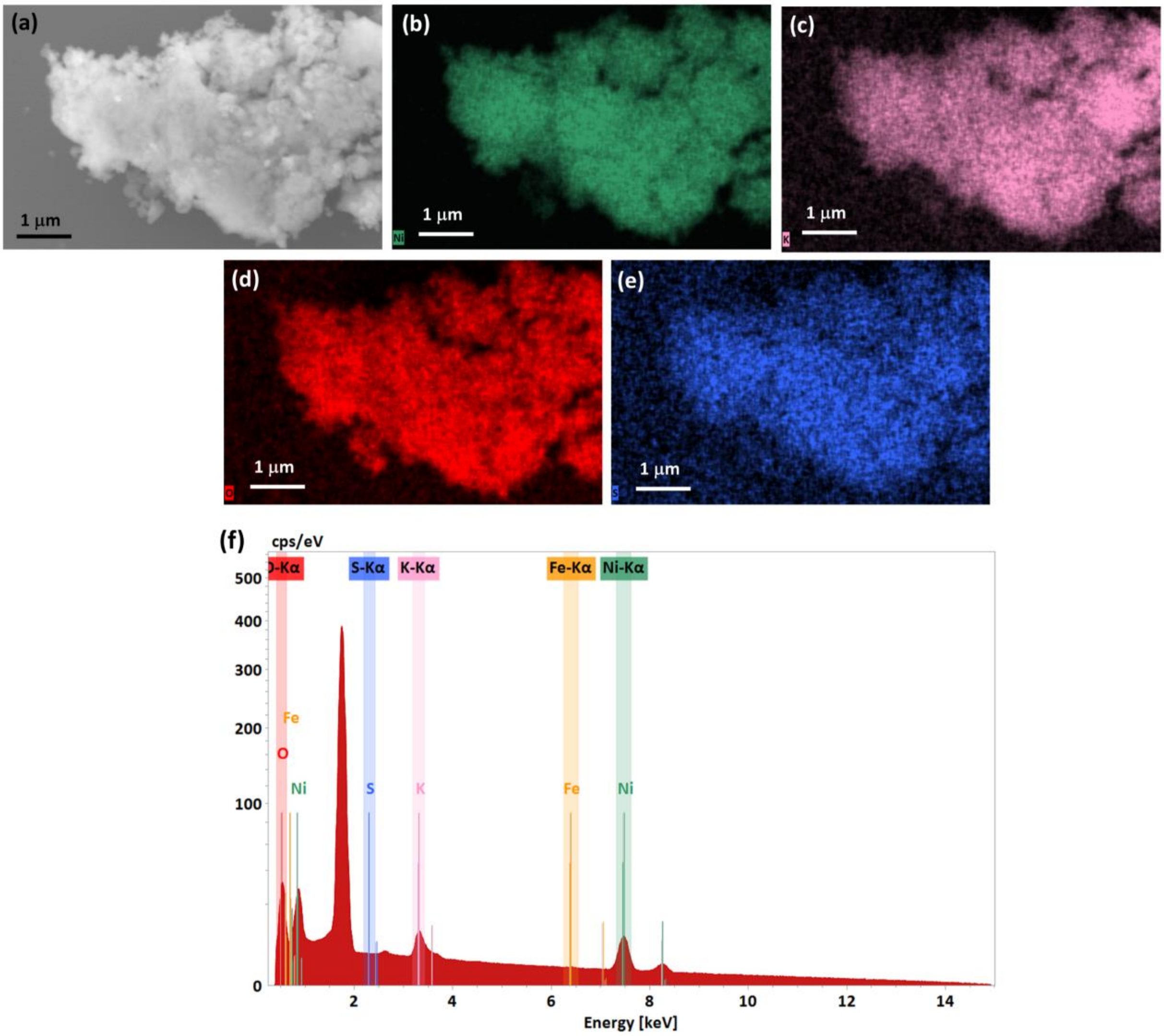

Figure S28. (a-e) SEM image and elemental mapping of NiS after 24 h CP at 10 mA/cm^2 (recorded by scratching off the film from FTO) shows a homogeneous distribution of Ni (green), K (purple) and O (red) in the sample. S mapping (blue) shows only noise and does not resolve the structure indicating the complete leaching of S from the precatalyst, as supported by the (f) absence S peak in the EDX spectrum. No significant Fe was detected in the sample.

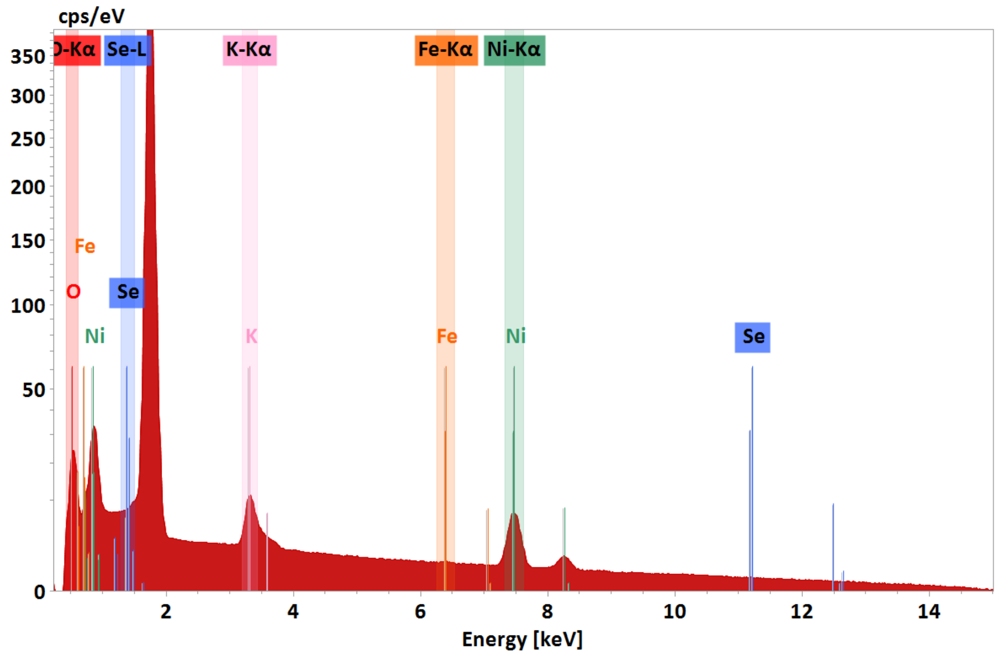

Figure S29. The EDX spectrum of NiSe after 24 CP at 10 mA/cm^2 shows the complete leaching of Se from the precatalyst. No significant Fe was detected in the sample.

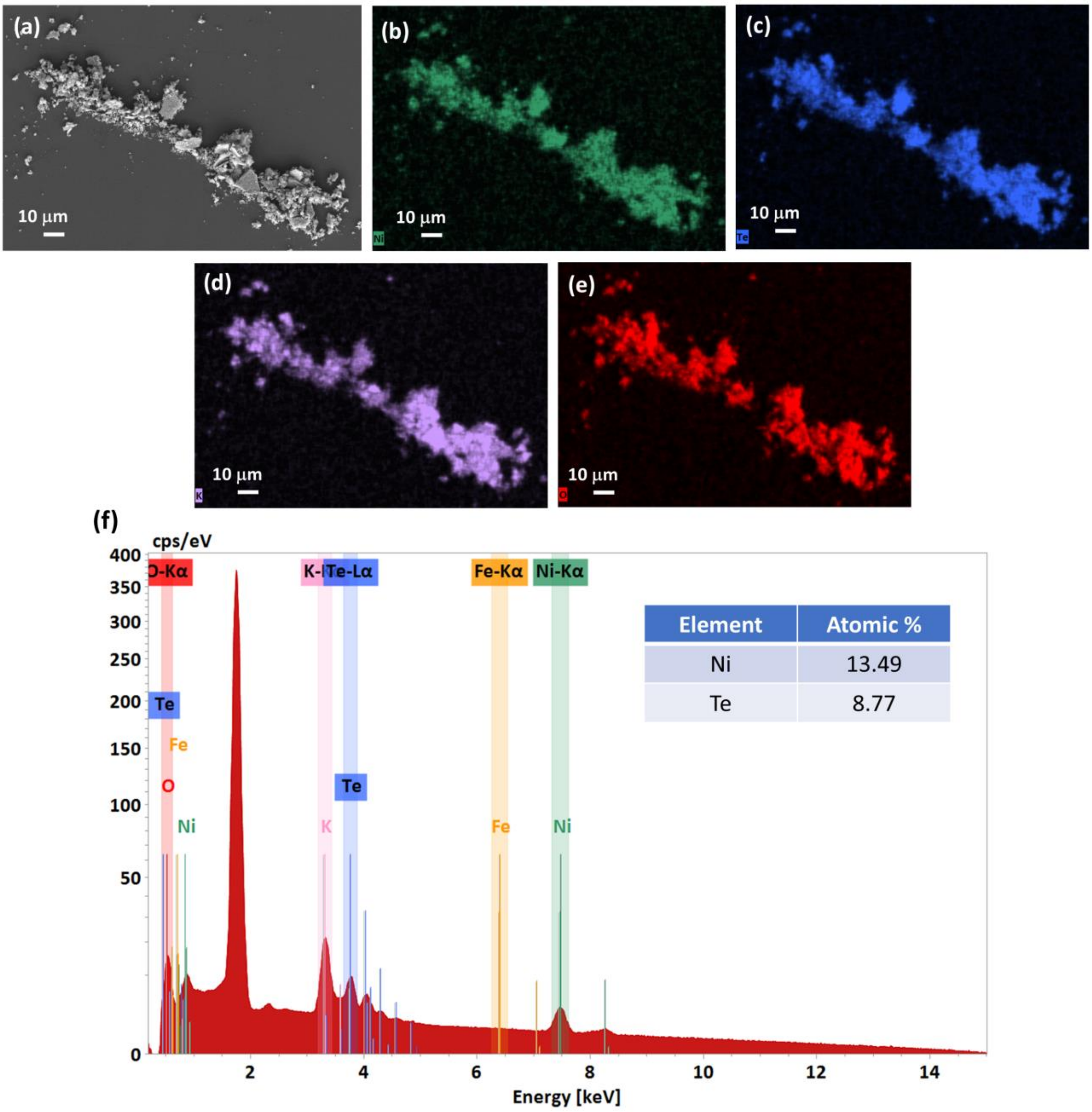

Figure S30. (a-e) SEM image and elemental mapping of NiTe after 24 h CP at 10 mA/cm^2 (recorded by scratching off the film from FTO) shows a homogeneous distribution of Ni (green), Te (blue), K (purple) and O (red) in the sample. (f) The EDX spectrum reveals a Ni:Te ratio of 1:0.65, indicating a ≈35% leaching of Te from the precatalyst. No significant Fe was detected in the sample.

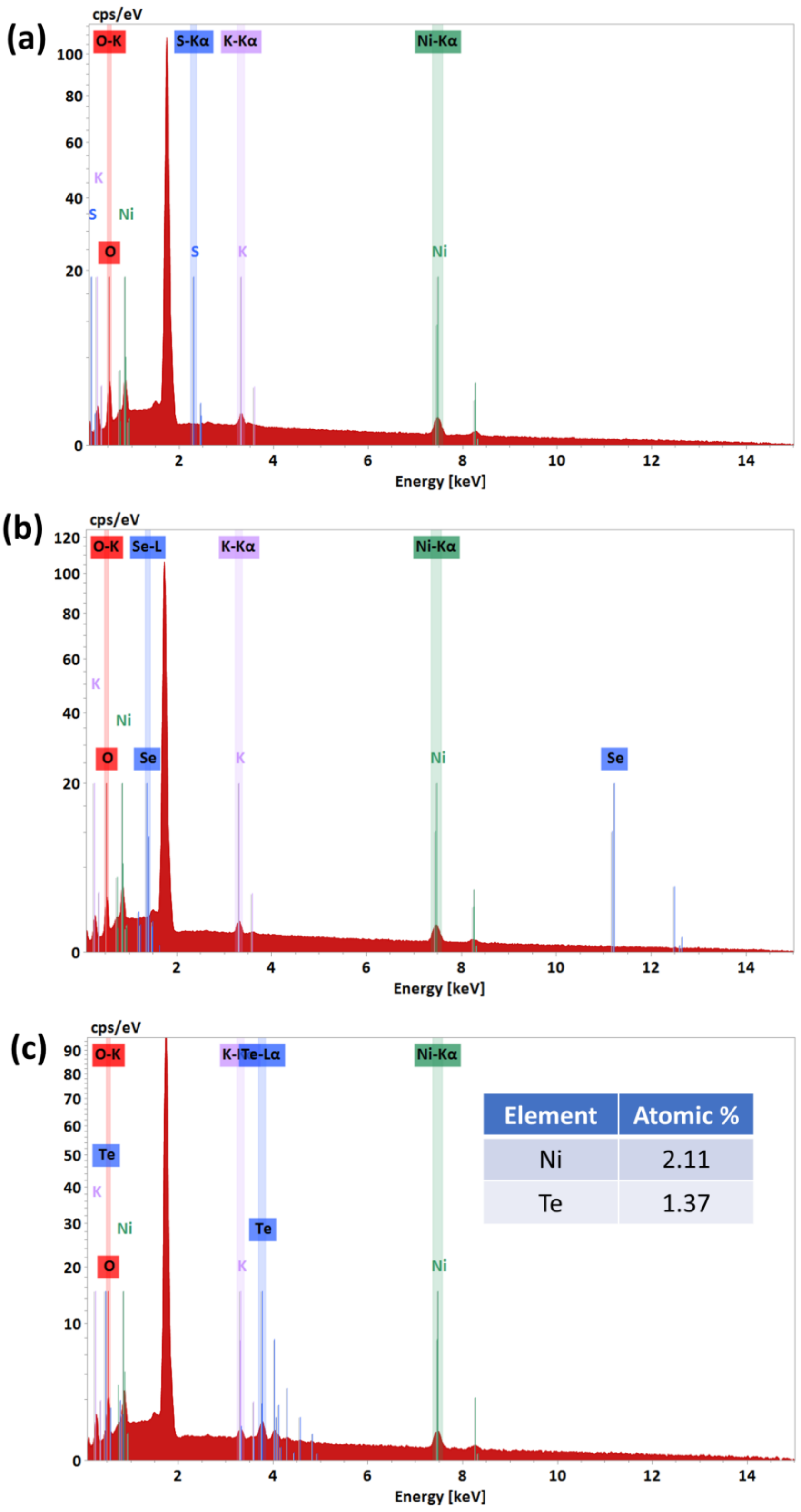

Figure S31. The EDX spectrum of (a) NiS, (b) NiSe and (c) NiTe after CV activation. S and Se are completely leached out while only ≈35% of Te (Ni:Te = 1:0.65) is leached out after CV activation, which is similar to the observation after 24 h CP at 10 mA/cm^2, indicating the formation of a stable active phase for the NiE precatalysts after CV activation itself.

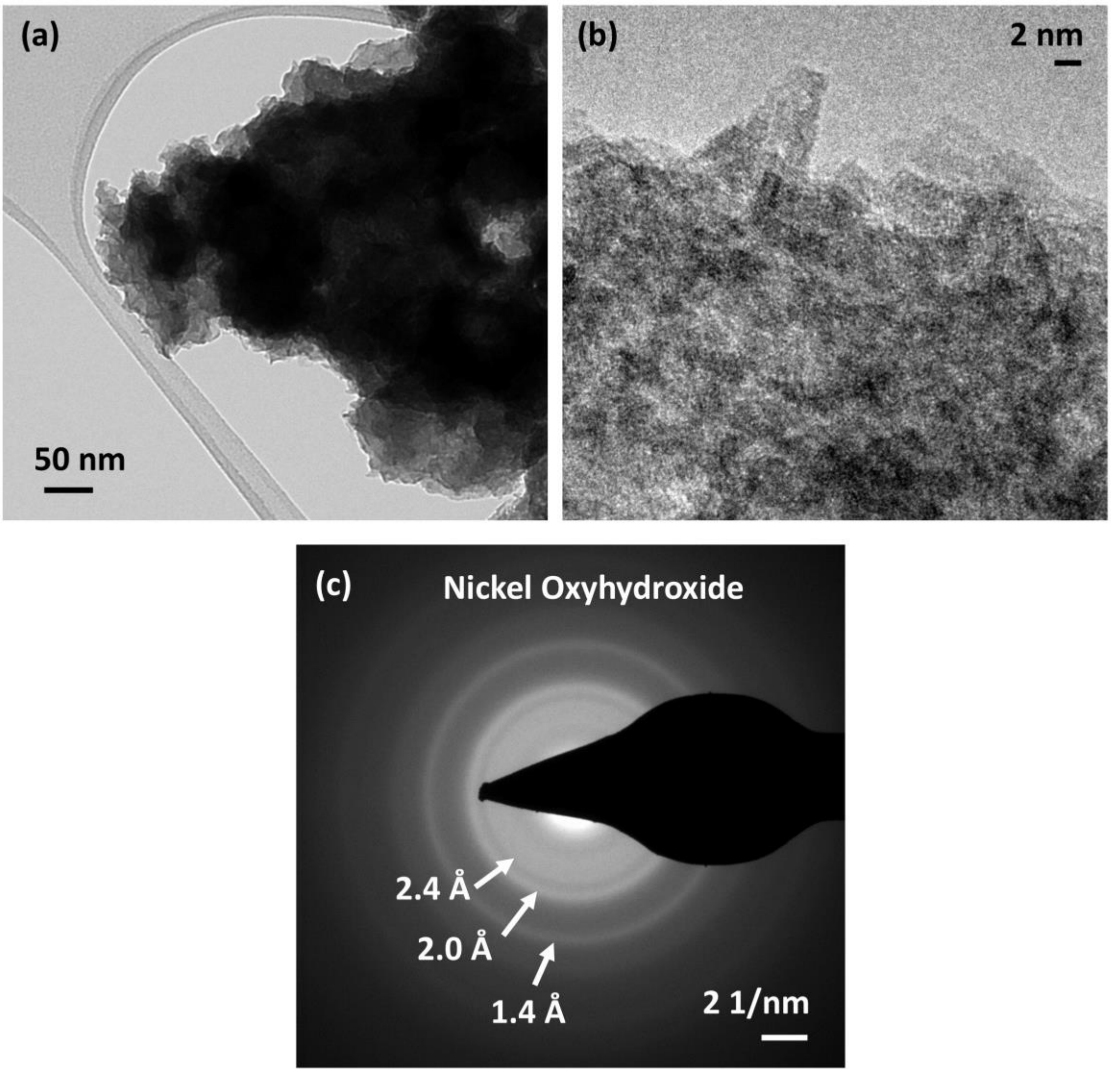

Figure S32. (a) TEM image of NiS after 24 h CP at 10 mA/cm^2. The (b) HR-TEM image and (c) SAED diffraction rings corresponding to lattice spacings of 2.4 Å, 2.0 Å and 1.4 Å confirm the complete transformation of NiS to a nickel oxyhydroxide (NiOOH) active phase (JCPDS 6-75).

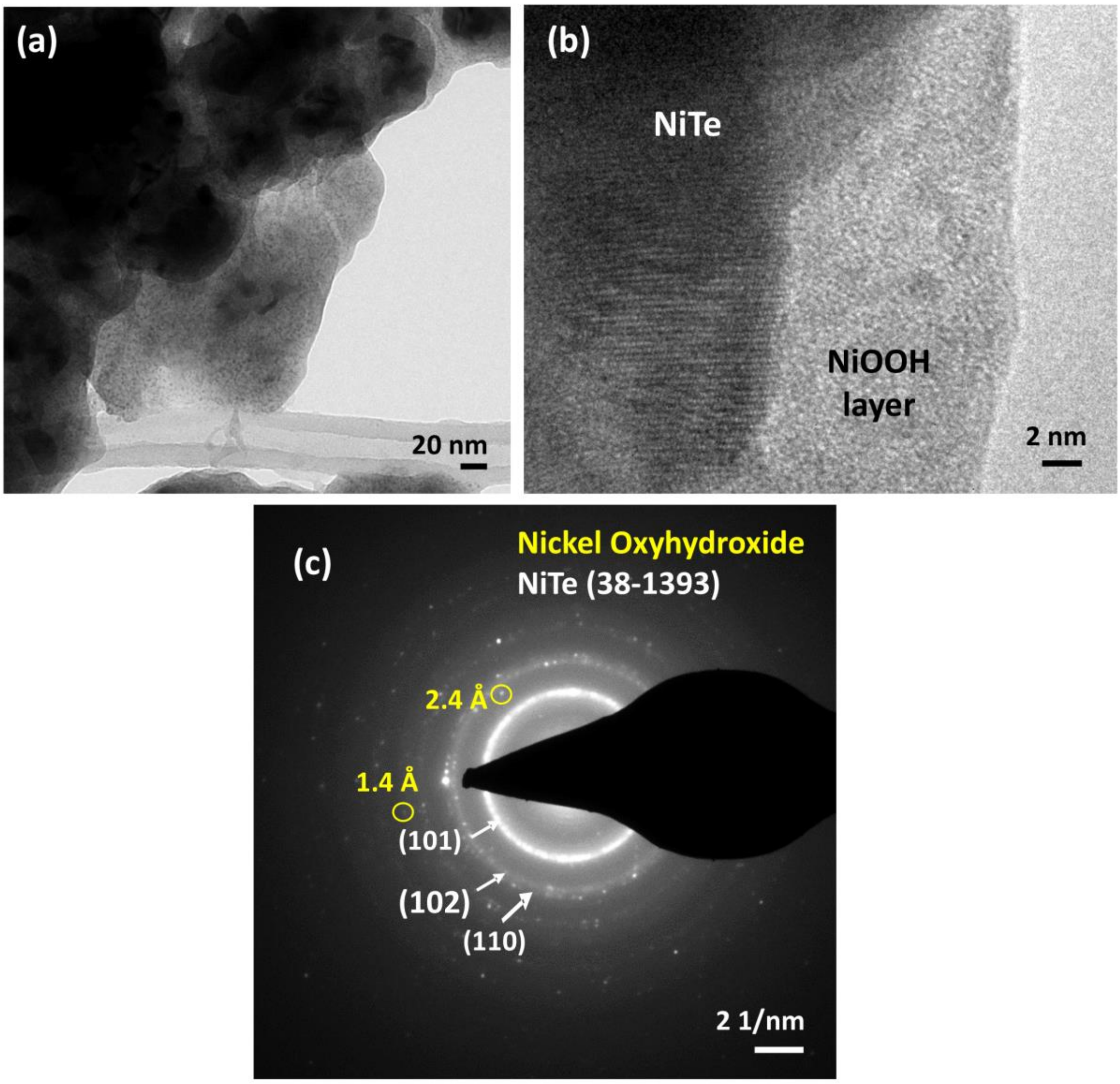

Figure S33. (a) TEM image of NiTe after 24 h CP at 10 mA/cm^2. The (b) HR-TEM image confirms partial transformation of the material to a NiOOH phase, with very low crystallinity. (c) The SAED pattern produced a majority of the diffraction spots and rings corresponding to the NiTe phase (JCPDS 38-1393) and a few spots corresponding to the NiOOH active phase (JCPDS 6-75).

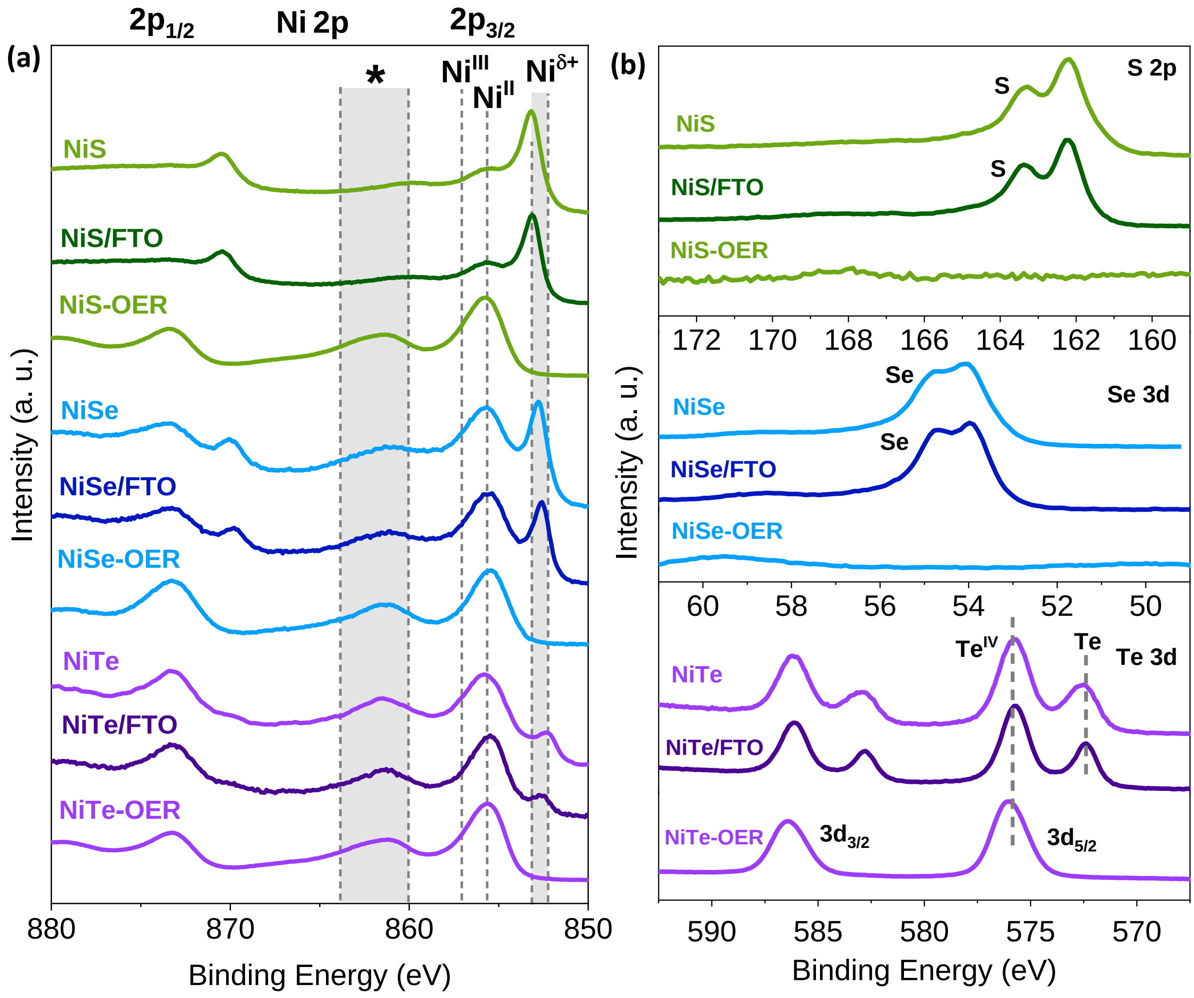

Figure S34. (a) The Ni 2p spectrum of the as-synthesized NiE powders indicates the presence of $Ni^{\delta+}$ and Ni^{II} species of the Ni chalcogenide phases.[16,24,40–42] Similar results were obtained for the as-deposited samples on FTO, confirming that the surface electronic structure did not alter after EPD. The post-OER NiE samples confirm the presence of Ni^{II} and Ni^{III} of the γ-$NiOOH_x$ phases (the * represents the satellite peaks region).[43,44] The XPS spectra of the chalcogens for the as-synthesized as well as as-deposited samples after EPD indicate the presence of S, Se and Te species characteristic of the respective Ni chalcogenide phases[16,24,40–42,45] The Te 3d spectrum shows additional Te^{IV} peaks due to the slight surface oxidation of NiTe.[46] The post-OER S 2p, and Se 3d spectra show the absence of any signal, while Te 3d spectrum indicates the presence of surface Te^{IV} species.[46]

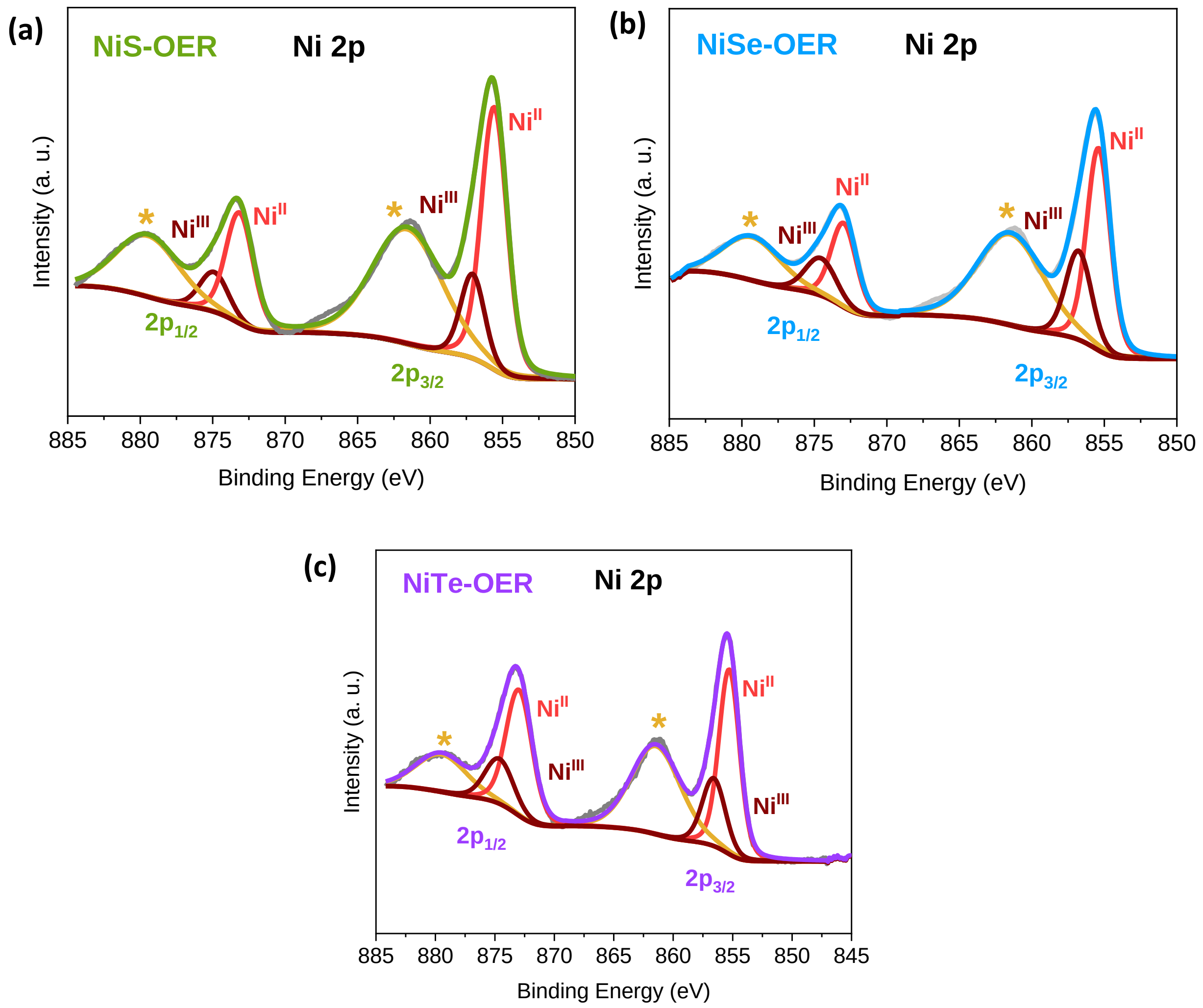

Figure S35. The deconvoluted Ni 2p spectrum of (a) NiS, (b) NiSe and (c) NiTe after 24 h CP at 10 mA/cm^2 reveal the presence of Ni^{II} (2p$_{3/2}$ 855.3 eV and 2p$_{1/2}$ 873.0 eV) and Ni^{III} (2p$_{3/2}$ 856.8 eV and 2p$_{1/2}$ 874.7 eV) predominant γ-NiOOH active phase.[43,44] The satellite peaks are represented by *.

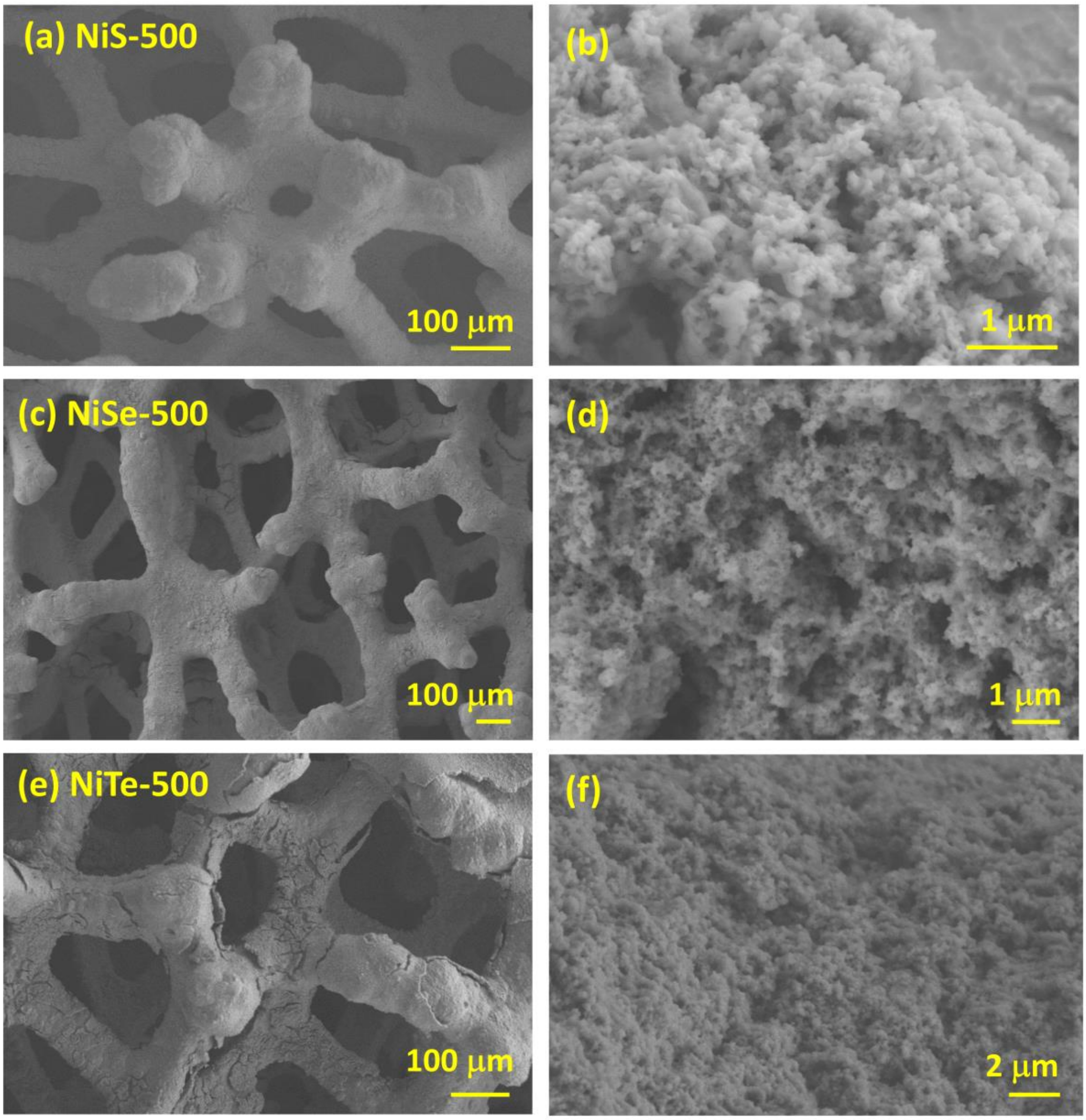

Figure S36. SEM images of (a, b) NiS/NF, (c, d) NiSe/NF and (e, f) NiTe/NF films after 24 h CP at 500 mA/cm^2 at different magnifications. The images reveal that the surfaces of the films were substantially altered after OER treatment at the high current density.

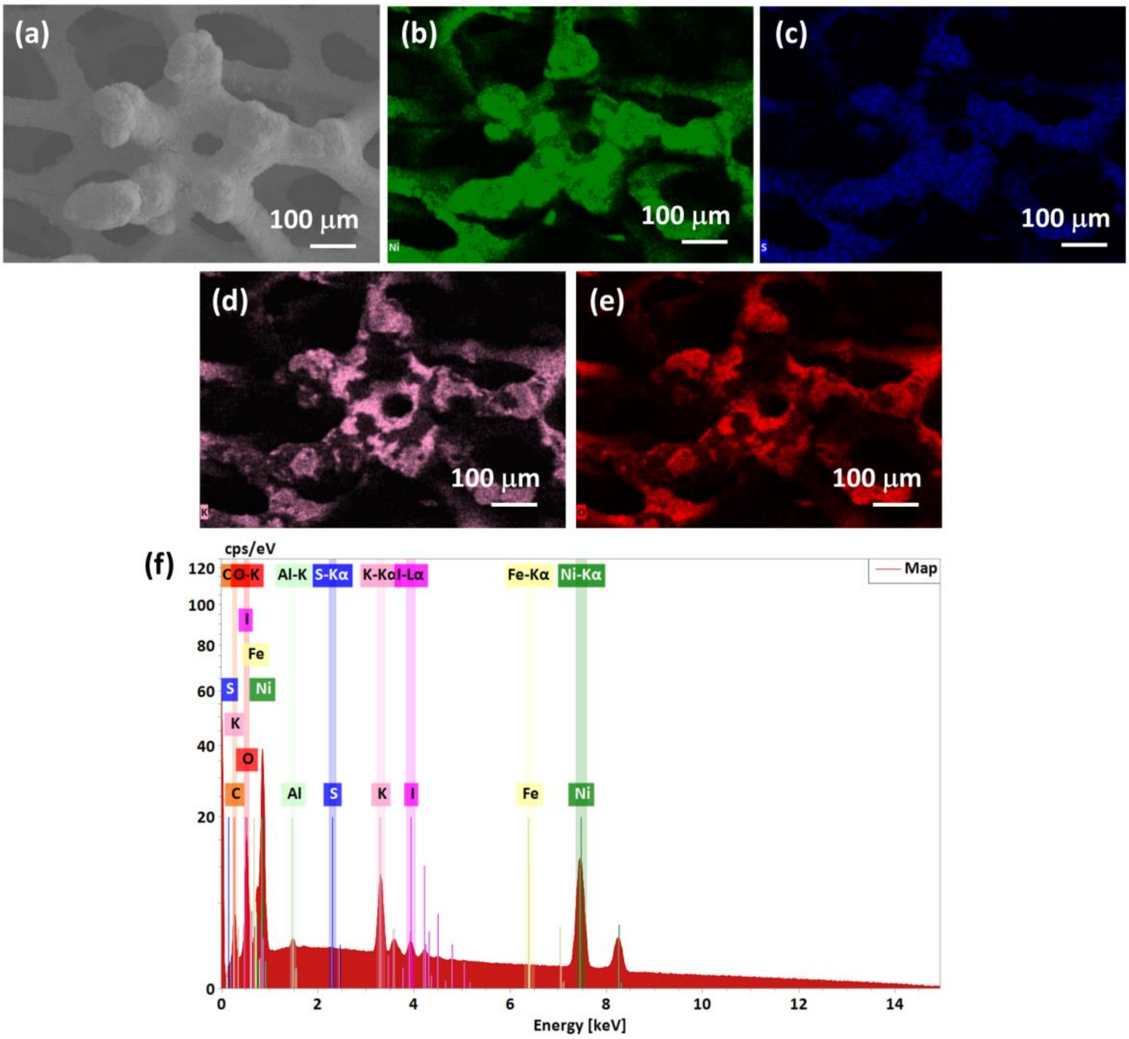

Figure S37. (a-e) SEM image and elemental mapping of NiS/NF after 24 h CP at 500 mA/cm^2 shows a homogeneous distribution of Ni (green), K (purple) and O (red) in the sample. S mapping (blue) shows only noise and does not resolve the structure indicating the complete leaching of S from the precatalyst, as supported by the (f) absence S peak in the EDX spectrum. No significant Fe was detected in the sample. Additional peaks of iodine and Al originate from the EPD process for film preparation and from the SEM holder, respectively.

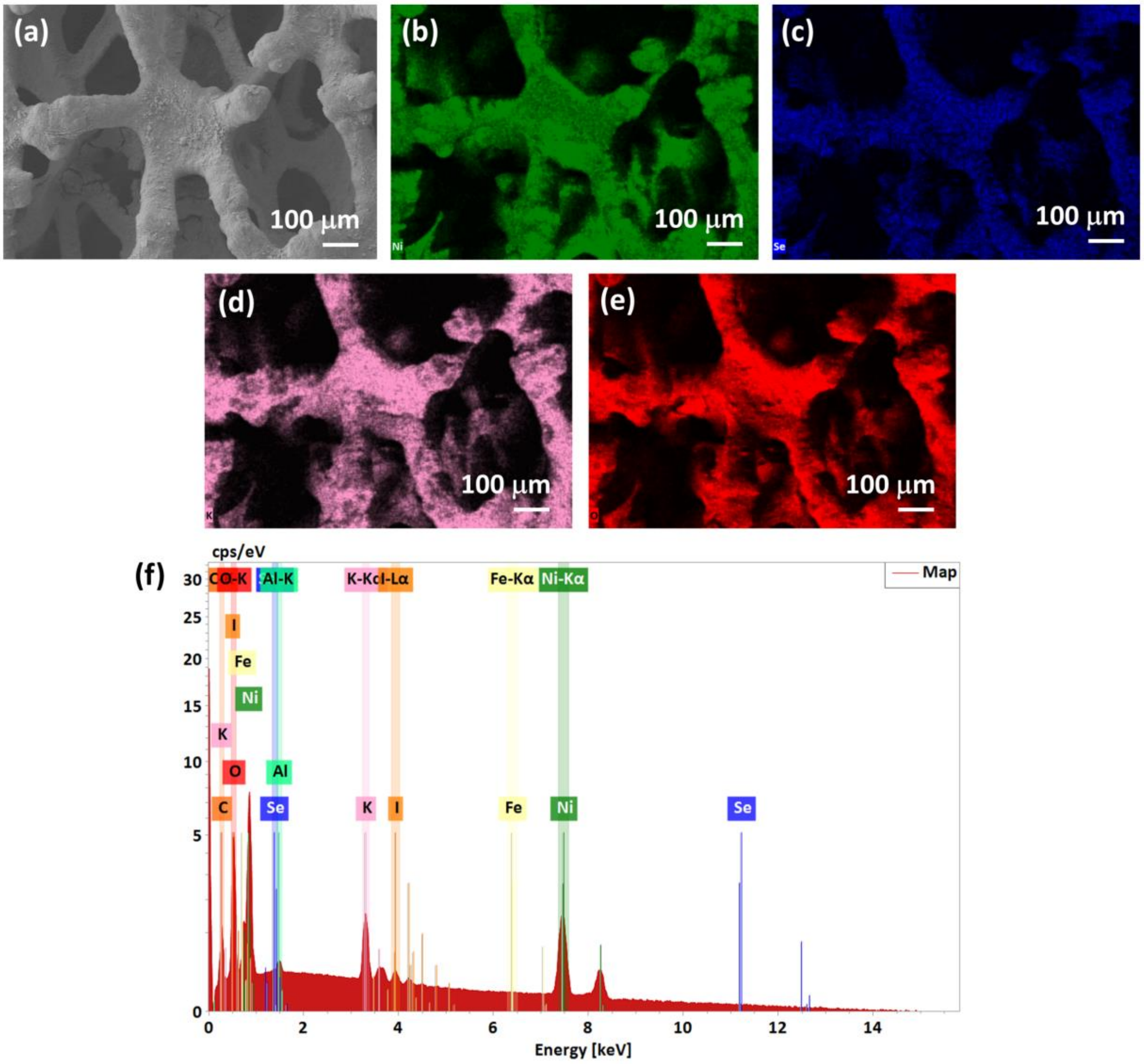

Figure S38. (a-e) SEM image and elemental mapping of NiSe/NF after 24 h CP at 500 mA/cm^2 shows a homogeneous distribution of Ni (green), K (purple) and O (red) in the sample. Se mapping (blue) shows only noise and does not resolve the structure indicating the complete leaching of Se from the precatalyst, as supported by the (f) absence Se peak in the EDX spectrum. No significant Fe was detected in the sample. Additional peaks of iodine and Al originate from the EPD process for film preparation and from the SEM holder, respectively.

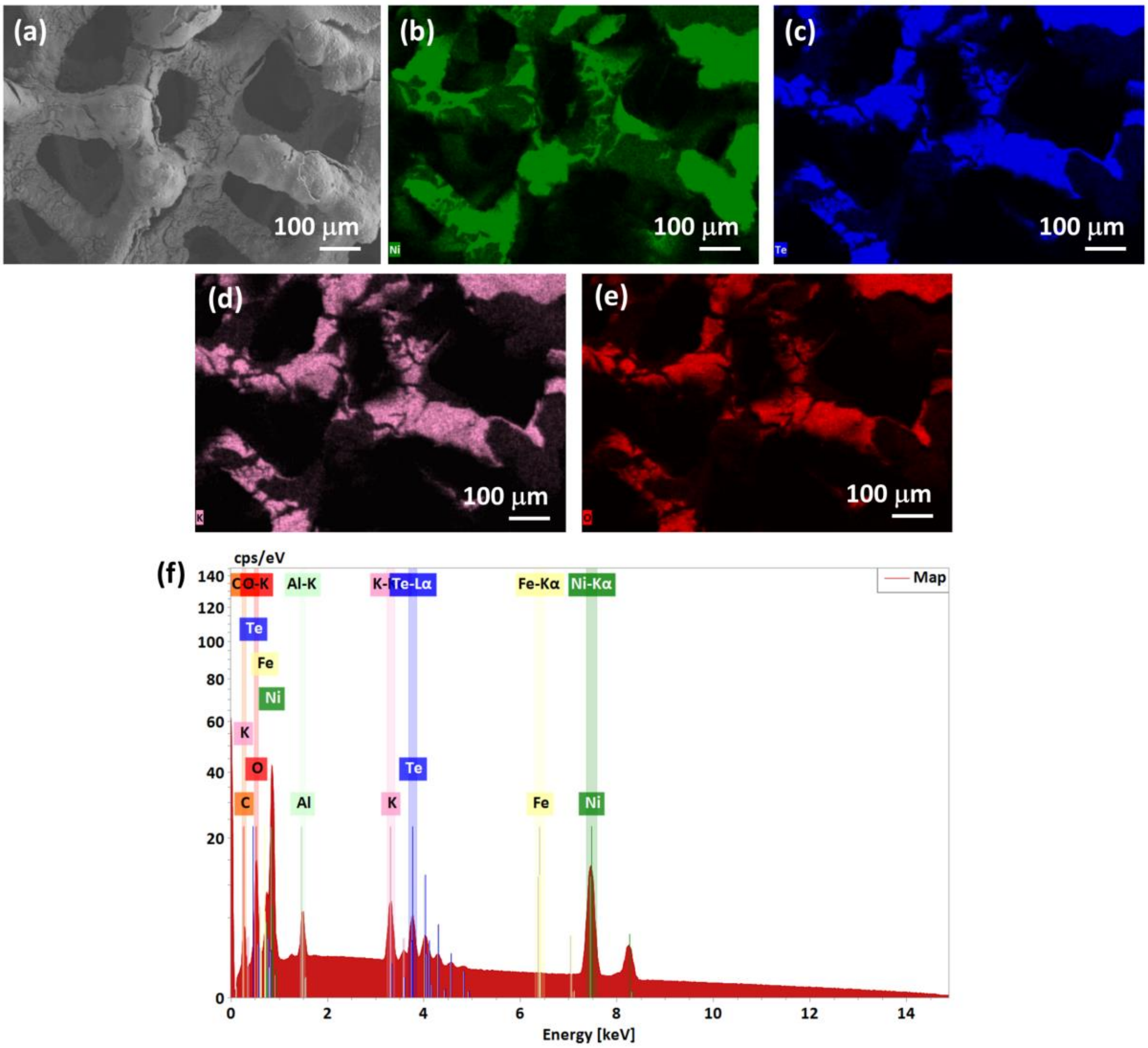

Figure S39. (a-e) SEM image and elemental mapping of NiTe/NF after 24 h CP at 500 mA/cm^2 shows a homogeneous distribution of Ni (green), Te (blue), K (purple) and O (red) in the sample. (f) The EDX spectrum reveals the presence of Te. No significant Fe was detected in the sample. The additional peak of Al originates from the SEM holder.

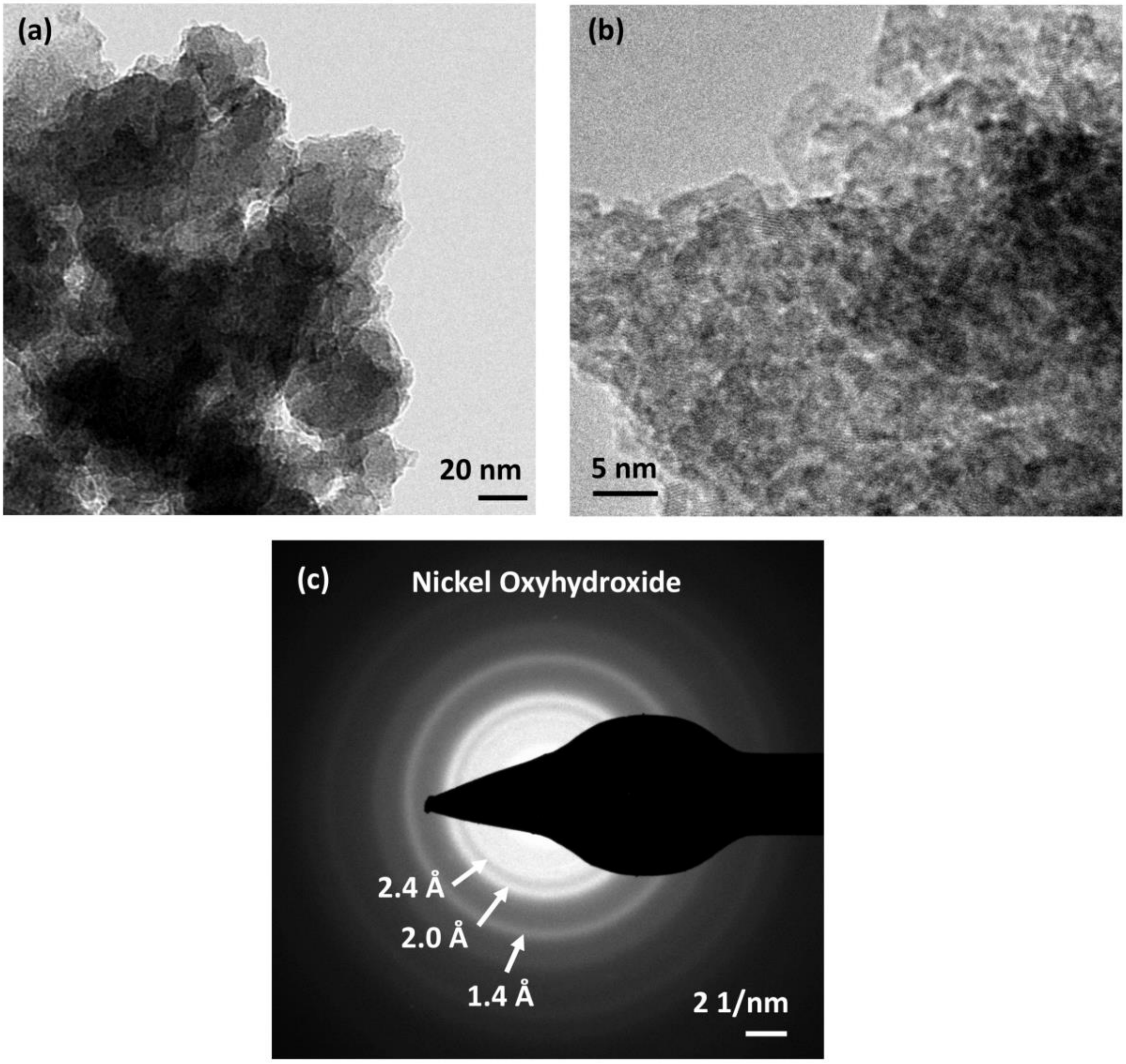

Figure S40. (a) TEM image of NiSe after 24 h CP at 500 mA/cm^2. The (b) HR-TEM image and (c) SAED diffraction rings corresponding to lattice spacings of 2.4 Å, 2.0 Å and 1.4 Å confirm the complete transformation of NiSe to a nickel oxyhydroxide (NiOOH) active phase (JCPDS 6-75).

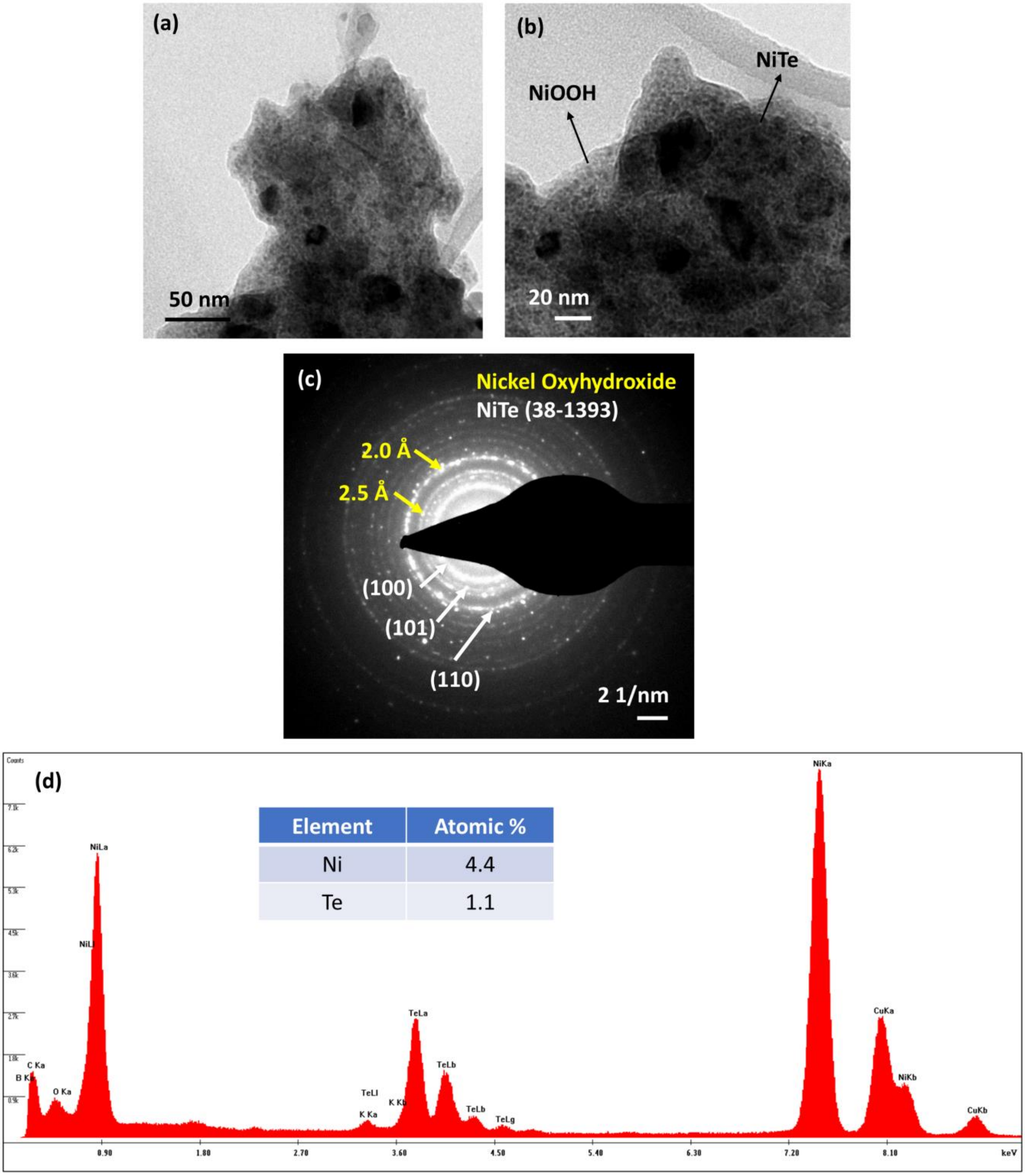

Figure S41. (a) TEM of NiTe after 24 h CP at 500 mA/cm^2. The (b) HR-TEM image confirms the partial transformation of the material to a NiOOH phase. (c) The SAED pattern shows diffraction rings corresponding to both the NiOOH active phase (JCPDS 6-75) and the NiTe phase (JCPDS 38-1393). (d) The EDX spectrum reveals a Ni:Te ratio of 1:0.25, indicating a ≈75% leaching of Te from the precatalyst.

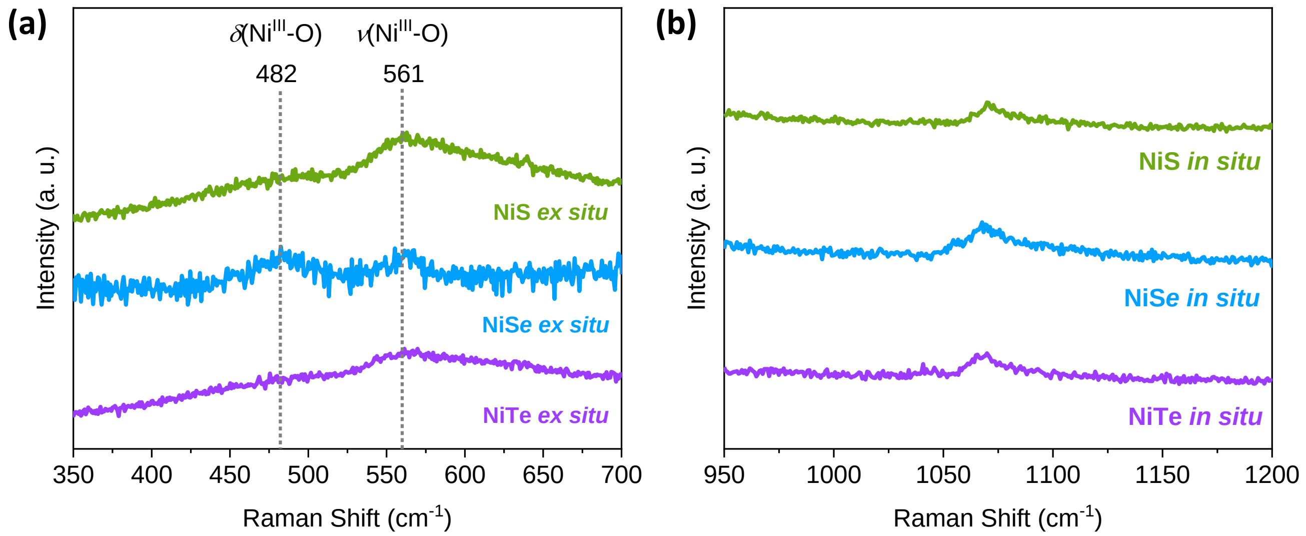

Figure S42. (a) The *ex situ* spectra of NiE after 24 h CP at 10 mA/cm^2 showing bands at 482 and 561 cm^{-1} corresponding to the Ni^{III}-O bending and stretching vibrations of the *γ*-NiOOH structure, similar to their *in situ* spectra (Figure 5a of the manuscript)[47,48] (b) Quasi *in situ* Raman of NiE after OER also showed a band at around 1070 cm^{-1}, a band that in ref.[47,48] was assigned to an 'active oxygen species'.

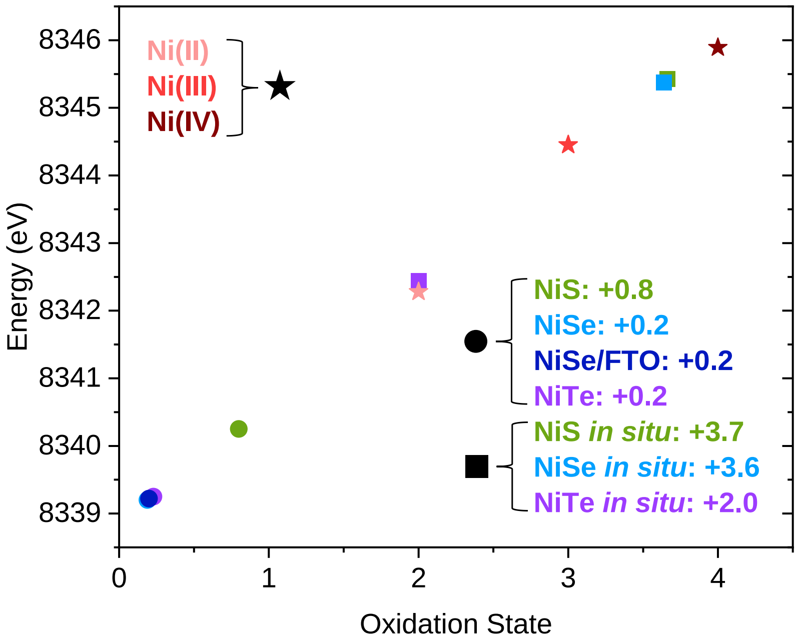

Figure S43. Valence states for Ni K edge in as-synthesized NiE powders; as-deposited NiSe on FTO; and NiE samples freeze-quenched at 1.56 V_{RHE} after 24 h CP at 10 mA/cm^2, extracted from their XANES data in Figure 5b of the manuscript. The Ni(II), Ni(III) and Ni(IV) spectra were collected for NiO, NiO_2Li, and $K_2(Ni(H_2IO_6)_2$, respectively.

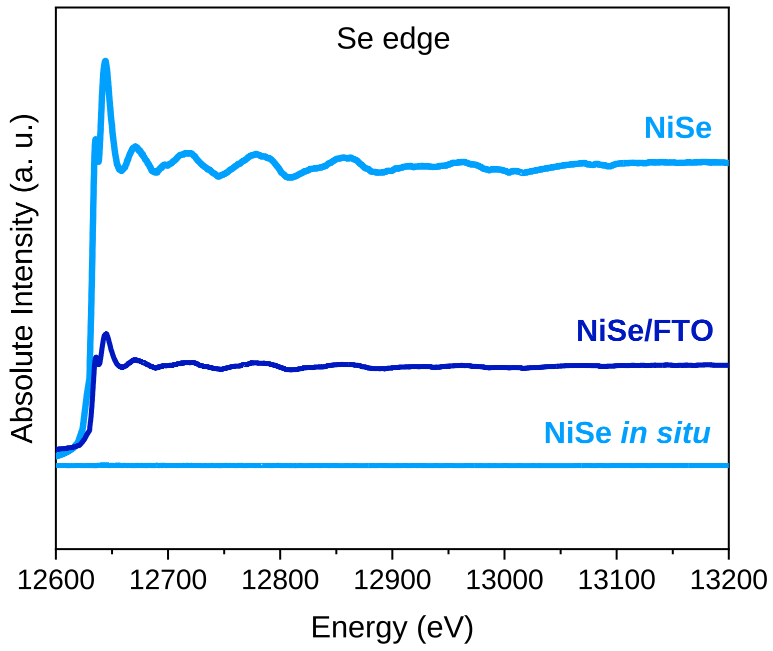

Figure S44. Absolute intensity of Se XANES fluorescence absorption of as-synthesized NiSe, as-deposited NiSe on FTO and NiSe freeze-quenched at 1.56 V_{RHE} after 24 h CP at 10 mA/cm^2. A case of “missing edge” is shown, in which the intensity of the signals of NiSe *in situ* OER is reduced to a minimum due to the loss of Se into the electrolyte.

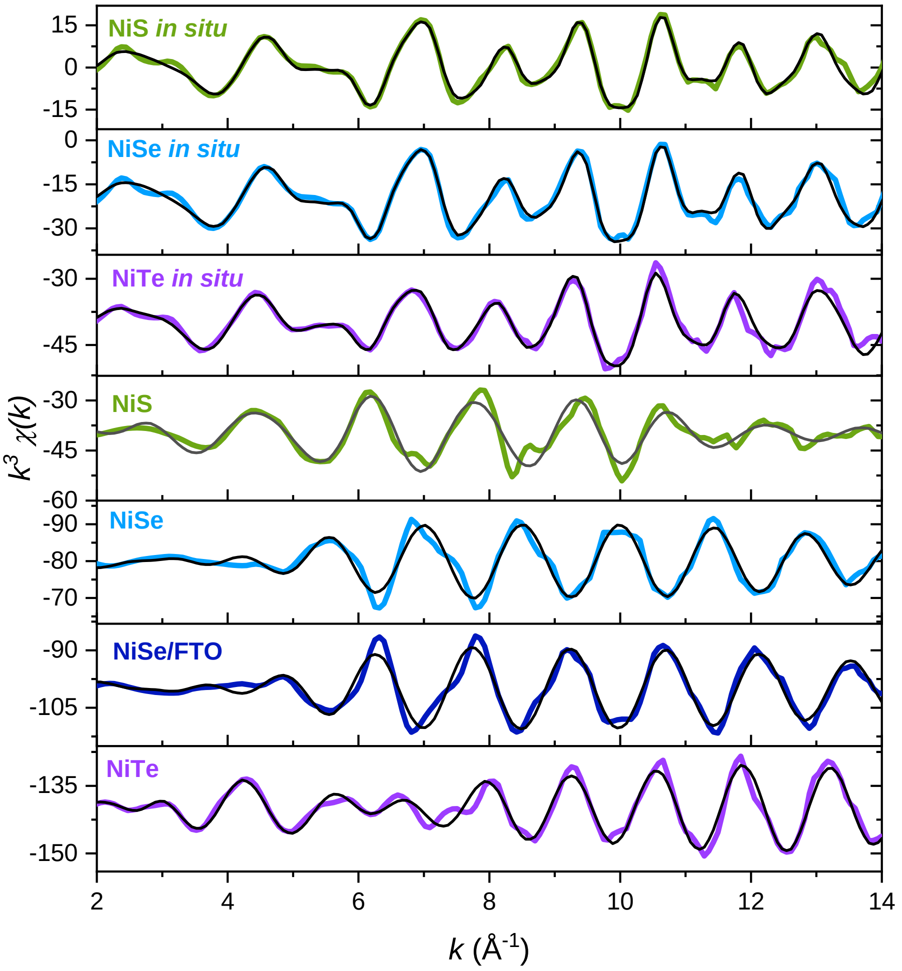

Figure S45. k^3-weighted experimental Ni EXAFS spectra of as-synthesized NiE powders; as-deposited NiSe on FTO; and NiE samples freeze-quenched at 1.56 V_{RHE} after 24 h CP at 10 mA/cm^2. The thin black lines represent the simulation of the experimental data. The simulation parameters are given in Table S13-S19.

Table S13. Simulation parameters for as-synthesized NiS. *N* is the EXAFS coordination number, *R* is the absorber-backscatter distance, and σ the Debye-Waller parameter. In all refinements, the amplitude reduction factor ($s_o^2(k)$) was 0.9. The Debye-Waller factors were set to reasonable values (0.050 Å) used in previous publications[49,50] on nickel compounds from our group if no error is given in order to grant stable fits and provide comparable shell populations. The errors represent the 68% confidence interval of the respective fit parameter. The obtained R_f value of the fit is 10.4%, which corresponds to a mean deviation between data and simulation of 10.4% in the distance range of interest (1–3.2 Å of reduced distance). The values marked with * were fixed to crystallographic values and not fitted (NiS phase JCPDS 1-1286; Ni-S: 2.261 Å, Ni-S: 2.383 Å, Ni-Ni: 2.534 Å, Ni-Ni: 3.150 Å).

shell	*N*	error	*R* [Å]	error [Å]	σ [Å]	error [Å]
Ni-S	3*	-	2.27	0.02	0.068	0.030
Ni-S	2*	-	2.35	0.04	0.052	0.029
Ni-Ni	2*	-	2.51	0.01	0.075	0.010
Ni-Ni	2*	-	3.14	0.02	0.087	0.011

Table S14. Simulation parameters for as-synthesized NiSe. Here, R_f = 10.9%. The values marked with * were fixed to crystallographic values and not fitted (NiSe phase JCPDS 2-892; Ni-Se: 2.498 Å, Ni-Ni: 2.665 Å).

shell	*N*	error	*R* [Å]	error [Å]	σ [Å]	error [Å]
Ni-Se	6*	-	2.46	0.01	0.075	0.002
Ni-Ni	2*	-	2.70	0.01	0.071	0.007

Table S15. Simulation parameters for as-deposited NiSe on FTO. Here, R_f = 10.2%. The values marked with * were fixed to crystallographic values and not fitted (NiSe phase JCPDS 2-892; Ni-Se: 2.498 Å, Ni-Ni: 2.665 Å).

**Shell**	***N***	**error**	***R* [Å]**	**error [Å]**	**σ [Å]**	**error [Å]**
Ni-Se	6*	-	2.46	0.01	0.073	0.002
Ni-Ni	2*	-	2.70	0.01	0.068	0.006

Table S16. Simulation parameters for as-synthesized NiTe. Here, R_f = 14.4%. The values were fitted by including a minor Ni-O shell, indicating slight pre-oxidation of the material (NiTe phase JCPDS 38-1393; Ni-Te: 2.647 Å, Ni-Ni: 2.680 Å).

**Shell**	***N***	**error**	***R* [Å]**	**error [Å]**	**σ [Å]**
Ni-O	1.3	0.4	2.06	0.02	0.050
Ni-Te	3.4	0.3	2.62	0.01	0.050
Ni-Ni	1.0	0.4	2.67	0.03	0.050

Table S17. Simulation parameters for NiS freeze-quenched at 1.56 V_{RHE} after 24 h CP at 10 mA/cm^2. The obtained R_f value of the fit is 11.2%, which corresponds to a mean deviation between data and simulation of 11.2% in the distance range of interest (1–6.0 Å of reduced distance).

Shell	*N*	error	*R* [Å]	error [Å]	σ [Å]
Ni-O	4.7	0.3	1.87	0.01	0.050
Ni-Ni	4.7	0.2	2.83	0.01	0.050
Ni-O	4.1	1.1	3.42	0.02	0.050
Ni-Ni	1.9	0.7	4.93	0.02	0.050
Ni-Ni	6.1	1.0	5.51	0.01	0.050

Table S18. Simulation parameters for NiSe freeze-quenched at 1.56 V_{RHE} after 24 h CP at 10 mA/cm^2. Here, R_f = 12.2%.

Shell	*N*	error	*R* [Å]	error [Å]	σ [Å]
Ni-O	4.8	0.3	1.87	0.01	0.050
Ni-Ni	4.8	0.2	2.84	0.01	0.050
Ni-O	2.9	1.1	3.41	0.03	0.050
Ni-Ni	2.3	0.7	4.93	0.02	0.050
Ni-Ni	5.7	1.0	5.51	0.01	0.050

Table S19. Simulation parameters for NiTe freeze-quenched at 1.56 V_{RHE} after 24 h CP at 10 mA/cm^2. Here, R_f = 14.3%.

Shell	*N*	error	*R* [Å]	error [Å]	σ [Å]
Ni-O	2.6	0.3	1.88	0.01	0.050
Ni-O	0.8	0.4	2.08	0.03	0.050
Ni-Te	1.0	0.2	2.62	0.01	0.050
Ni-Ni	1.9	0.3	2.86	0.01	0.050
Ni-O	1.4	1.2	3.46	0.05	0.050
Ni-Ni	1.1	0.8	4.96	0.03	0.050
Ni-Ni	2.5	1.0	5.56	0.02	0.050

Characterization for BA and HMF Oxidation

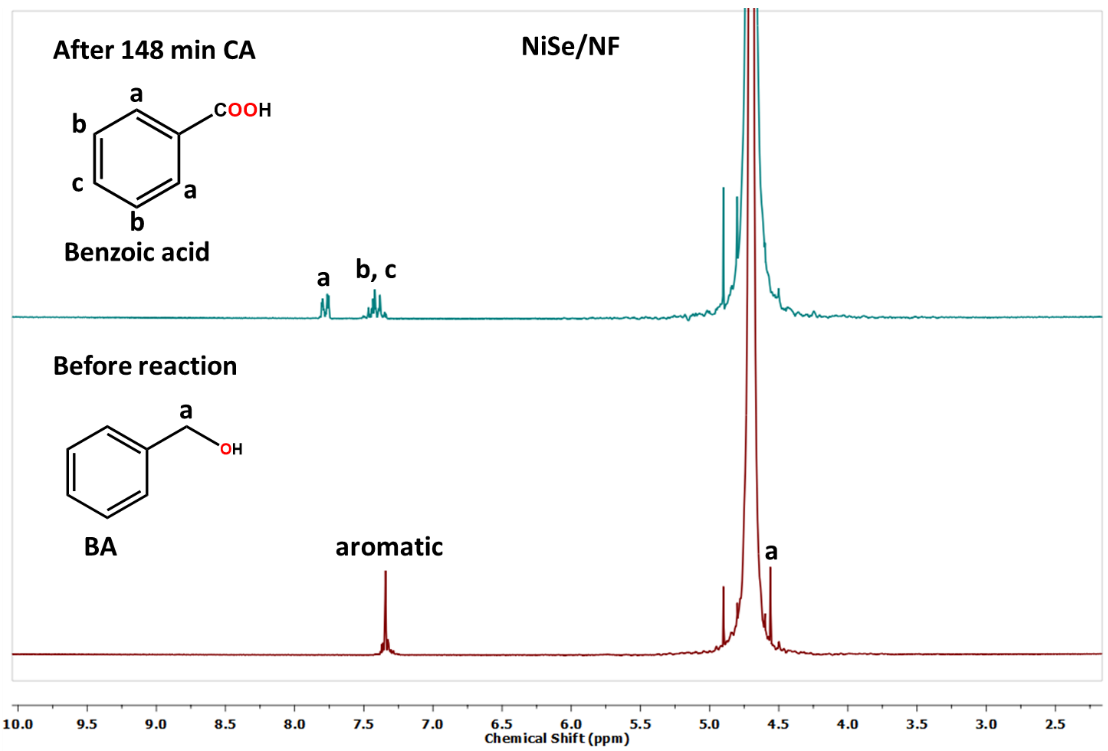

Figure S46. ^{1}HNMR spectrum of reaction mixture after 148 min CA of 0.1 M BA in 1 M KOH, with NiSe/NF. It shows a complete conversion of BA to benzoic acid, therefore giving a FE of ≈100%.

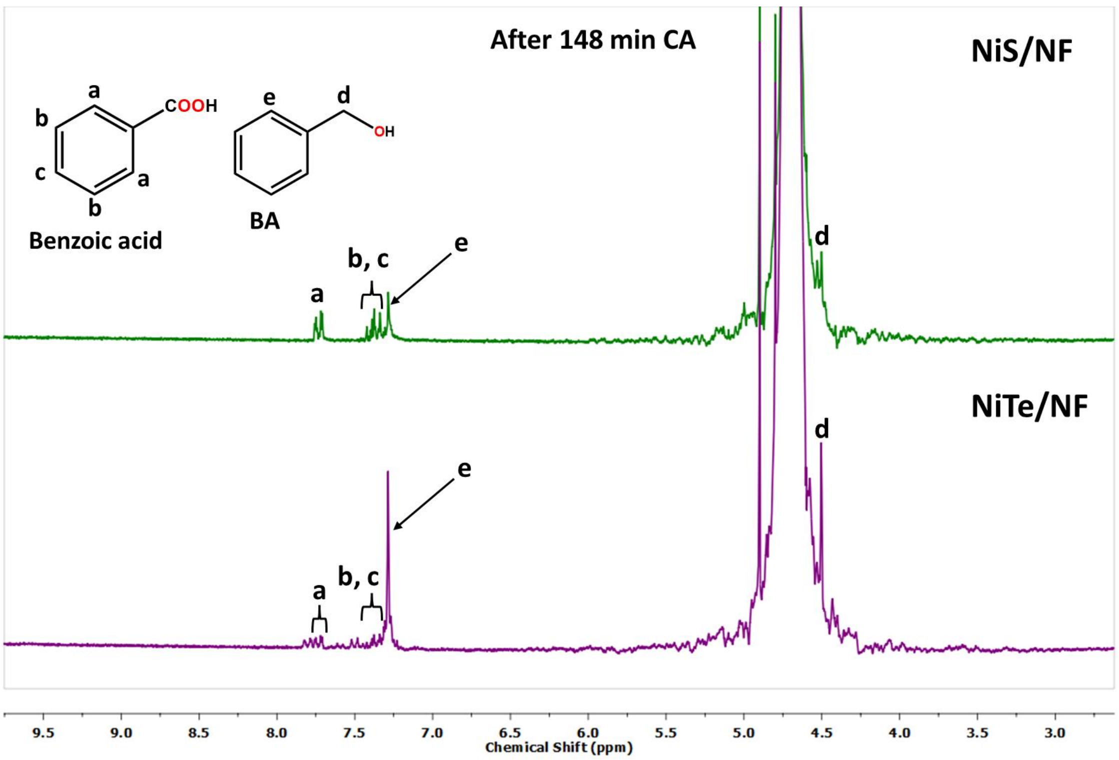

Figure S47. ^{1}HNMR spectrum of reaction mixture after 148 min CA of 0.1 M BA in 1 M KOH, with NiS/NF and NiTe/NF. NiS/NF shows ≈64% and NiTe/NF shows a ≈20% yield of benzoic acid.

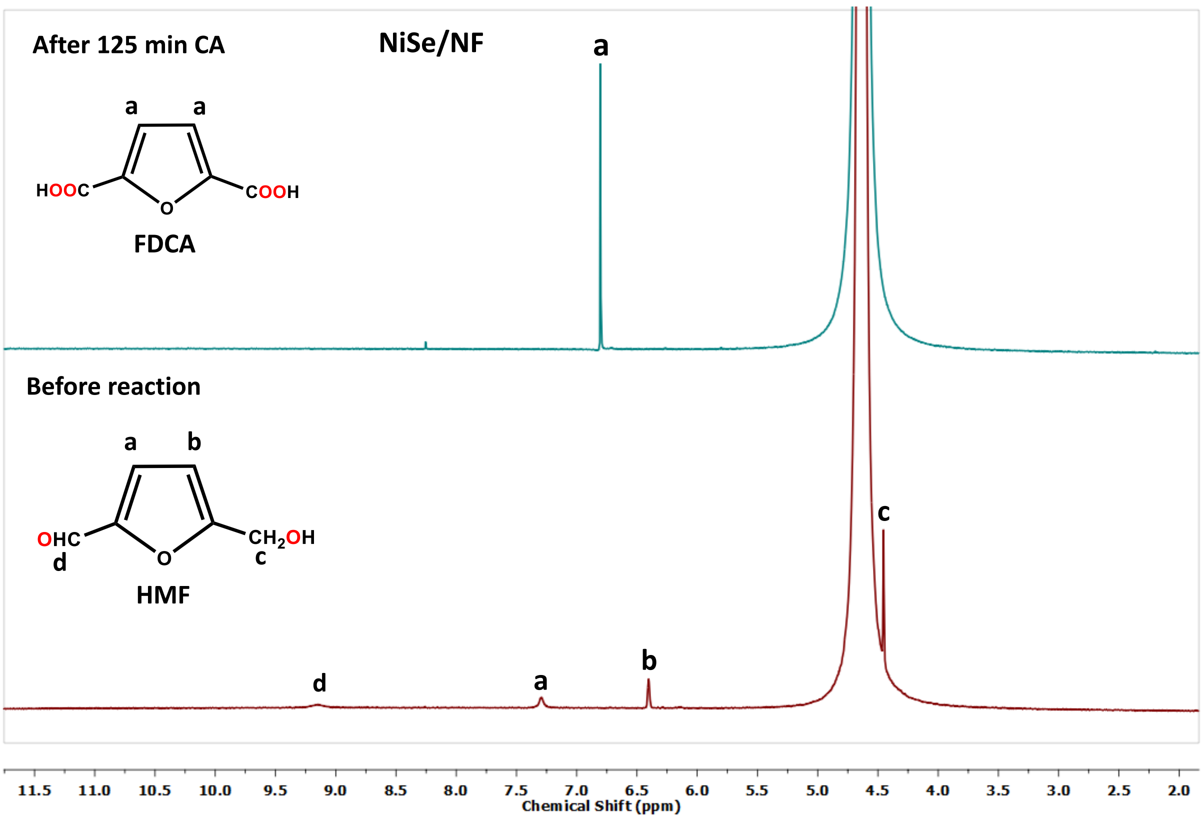

Figure S48. ^{1}HNMR spectrum of reaction mixture after 125 min CA of 0.1 M HMF in 1 M KOH, with NiSe/NF. It shows a ≈99% conversion of HMF to FDCA, therefore giving a FE of ≈99%. A minute amount of formate (8.4 ppm) is formed as a degradation product.[51]

Table S20. Comparison of FE of the Ni Chalcogenides in this work with other reported Ni-based materials for the oxidation of BA to benzoic acid.

**Material**	**Substrate**	**FE (%)**	**Reference**
NiSe	*NF*	*≈100*	*This work*
NiS	NF	87	[31]
NiO/Ni_3S_2	CC	94	[52]
$Co_{0.83}Ni_{0.17}/AC$	CP	96	[53]
NF-Plasma	NF	>95	[5]
$NiCo_2O_4$	NF	≈100	[54]
$Ni(OH)_2$ nanosheet	NF	99	[55]
hp-Ni	NF	98	[56]
NiMoO-Ar	NF	93	[57]
Ni_2P	NF	92.4	[58]
h-$Ni(OH)_2$	Carbon Fibre	98.62	[59]
Ni_3N	NM	97	[60]
N–Mo–Ni	NF	98.7	[61]
Ni@NC-280	CC	≈100	[62]
MXene/Ni_3N	CP	96	[63]
CuO@$Ni(OH)_2$	CF	92	[64]
amorphous Ni-Co-H	NF	≈100	[65]
C,N-co-doped ZnO/Co_3O_4@$Ni(OH)_2$	NF	97	[66]

Here, AC = Activatied Carbon, hp = hierarchically porous, NM = Nickel Mesh

Table S21. Comparison of FE of the Ni Chalcogenides in this work with other reported Ni-based materials for the oxidation of HMF to FDCA.

Material	Substrate	FE (%)	Reference
NiSe	*NF*	*≈99*	*This work*
NiS	NF	79	[31]
Ni_3N@C	NF	99	[67]
$Ni_{0.9}Cu_{0.1}(OH)_2$	CP	91.2	[68]
NF-Plasma	NF	>95	[5]
NiS_x/Ni_2P	CC	95.1	[69]
Ni_xB	NF	≈100	[70]
Co-Ni_xP@C	Ni-Co foam	98.9	[71]
S–Ni@C	CC	96	[72]
NiP	NF	96	[73]
NiAs	NF	96	[73]
Cu/Ni_3S_2-R	NF	≈100	[74]
NiCo PBA	CP	≈100	[75]
NiCu NTs	NF	96.4	[76]
$NiNO_3$	CC	98	[77]
$NiFeNO_3$	CC	98	[77]
Mo-$Ni_{0.85}$Se	NF	95	[78]
Ni−Cu	NF	99.7	[79]
$Ni(OH)_2$	NF	>99	[80]
Ni_2S_3	NF	94	[81]
NiRu@PCNS	NF	99.7	[82]
$Mn_{0.2}$NiS	GF	94.2	[83]
NiB_x–$P_{0.07}$	CP	92.5	[84]

Here, PBA = Purssian Blue Analogue, NT = Nanotube, NS = Nanosheets, PCNS = carbonaceous nanospheres, GF = Graphite Felt

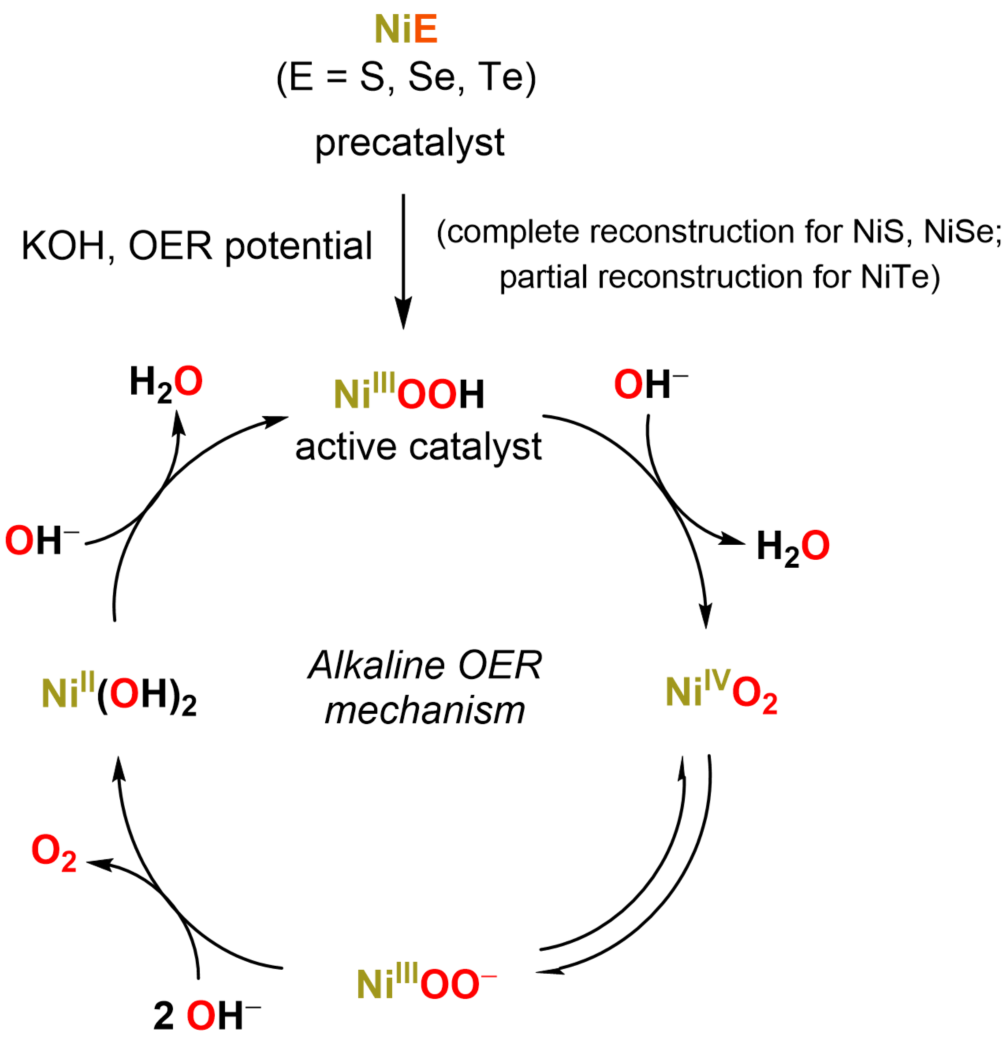

Figure S49. Proposed mechanism of alkaline OER for the NiE materials.[38,39,85,86]

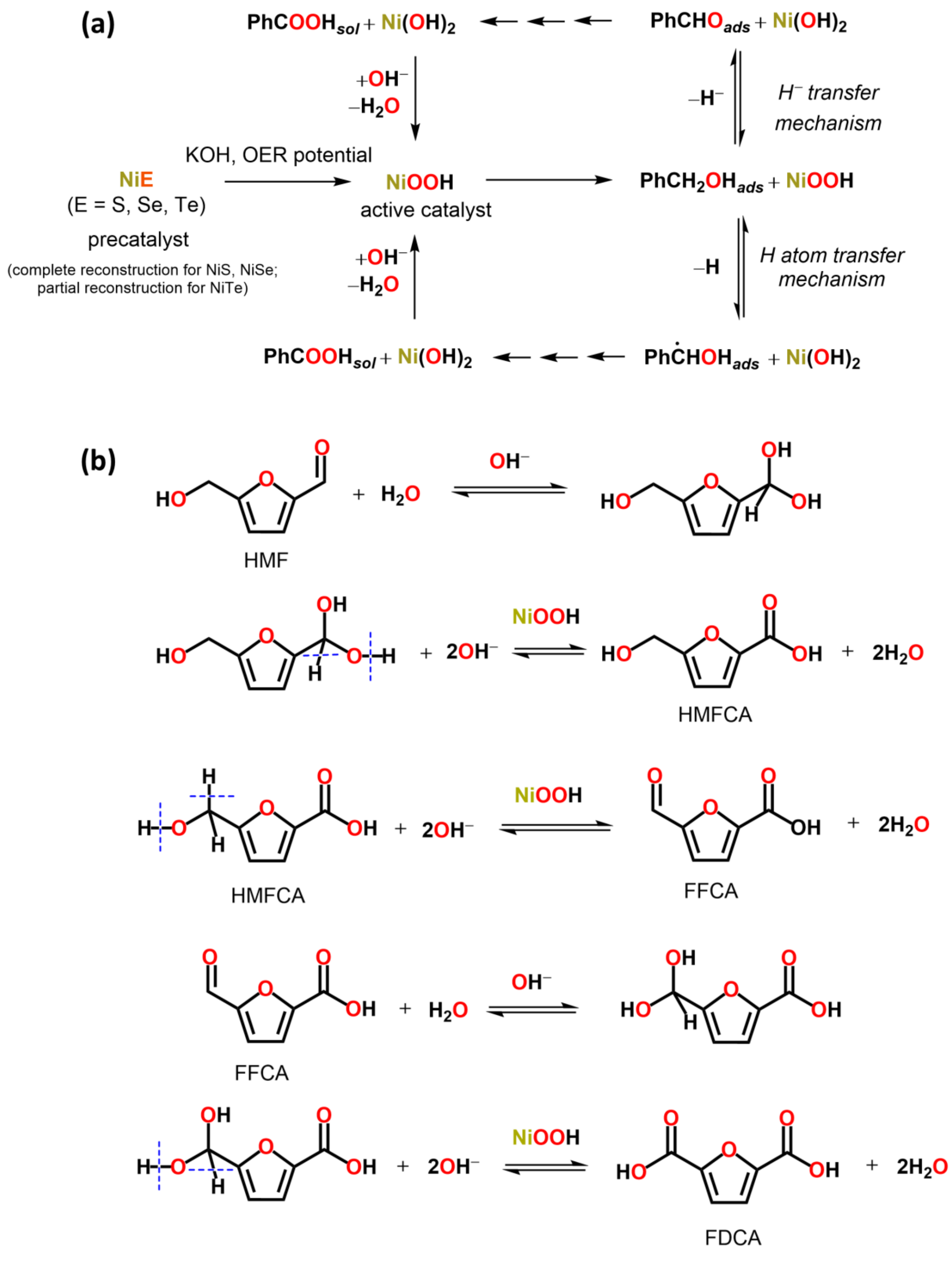

Figure S50. Proposed mechanism of (a) BA oxidation and (b) HMF oxidation (here HMFCA = 5-hydroxymethyl-2-furancarboxylic acid, FFCA = 5-formyl-2-furancarboxylic acid) for the NiE materials under alkaline conditions (here, pH = 13.89).[87]

Characterization and OER measurements for reference materials

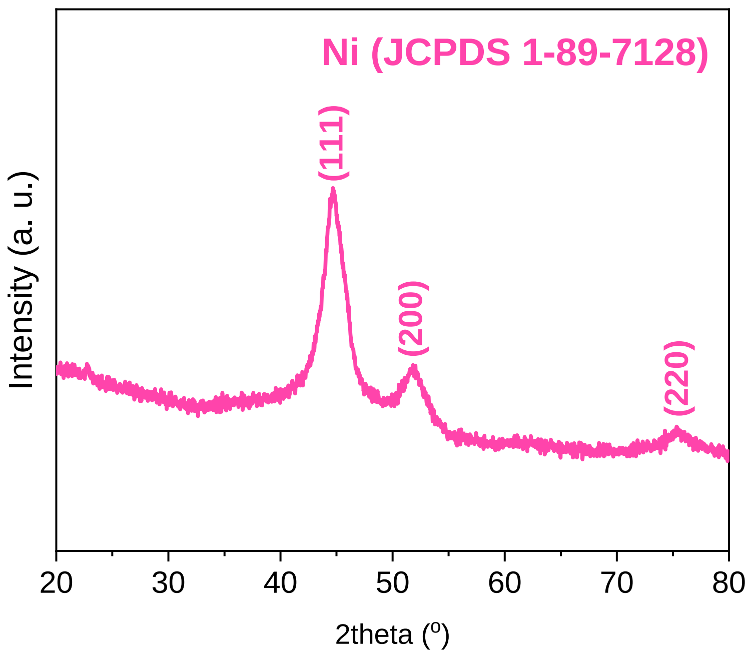

Figure S51. PXRD pattern of as-synthesized Ni nanoparticles (Ni NP).

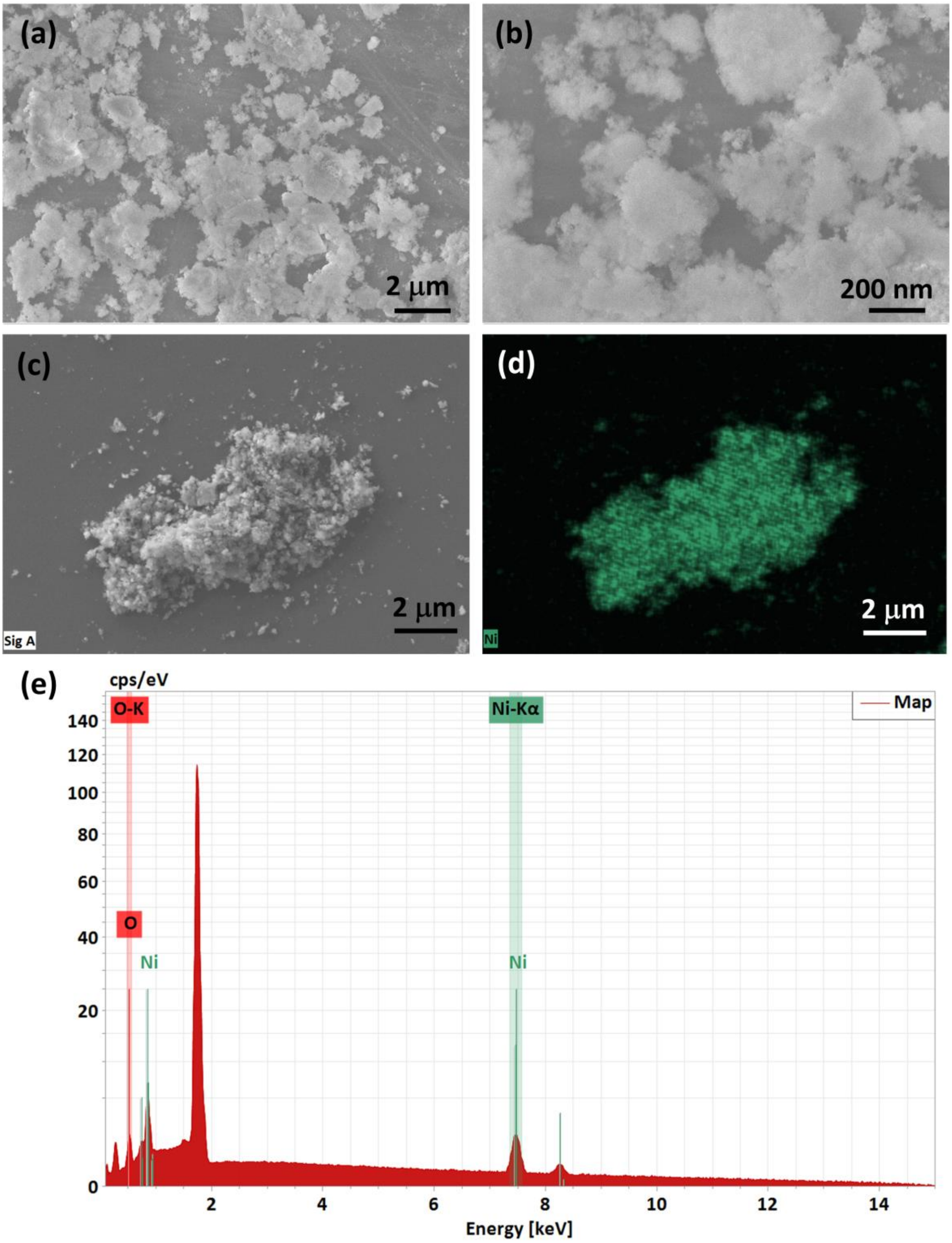

Figure S52. (a, b) SEM image of reference Ni NP at different magnifications. The as-synthesized Ni NP shows agglomerated particles. (c, d) Elemental mapping shows the homogeneous distribution of Ni (green) throughout the sample. (e) SEM-EDX spectrum confirms the presence of only Ni in the sample.

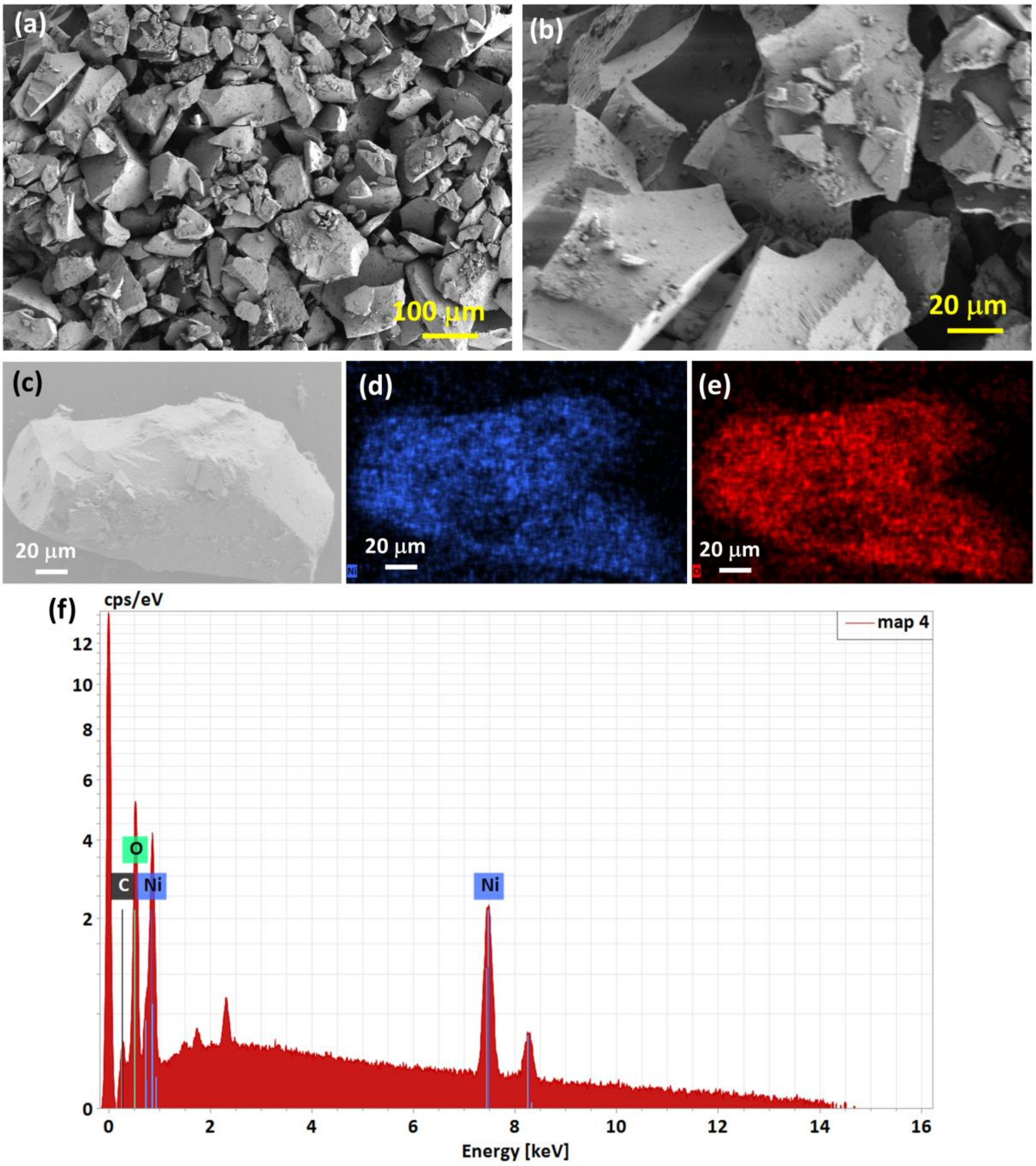

Figure S53. (a, b) SEM image of reference NiOOH at different magnifications. (c, d) Elemental mapping shows the homogeneous distribution of Ni (green) and O (red) throughout the sample. (e) SEM-EDX spectrum confirms the presence of Ni and O in the sample.

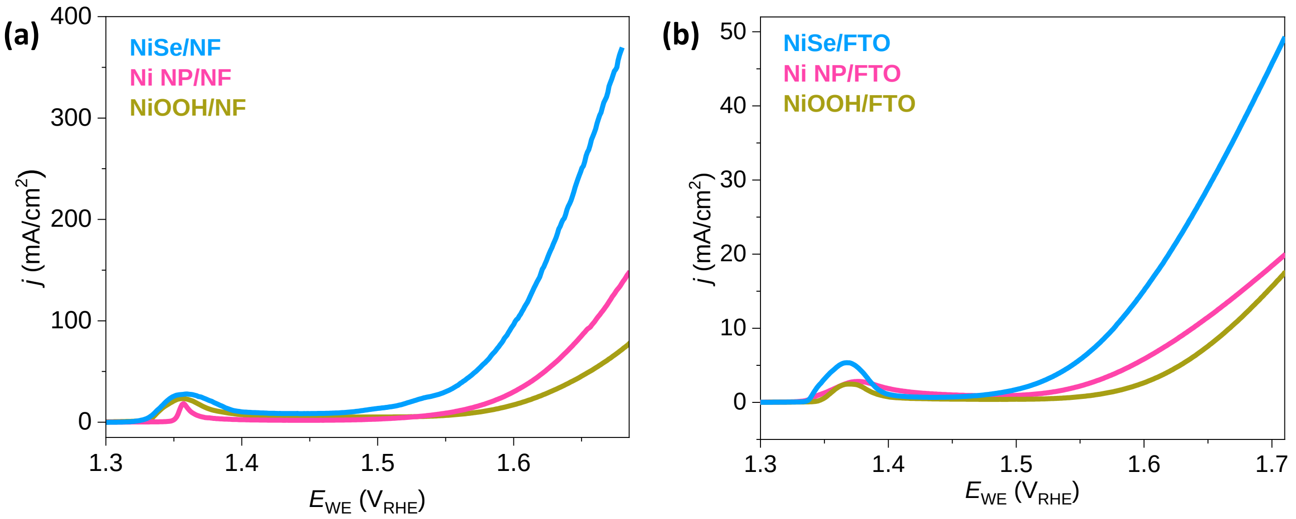

Figure S54. Electrochemical measurements for OER recorded in 1 M KOH, at 25 °C for NiSe, Ni NP and NiOOH on (a) NF at a scan speed of 1 mV/s, with 0.9 ± 0.1 mg/cm^2 loading, and on (b) FTO at a scan speed of 5 mV/s, with 0.4 ± 0.1 mg/cm^2 loading.

OER activity normalization

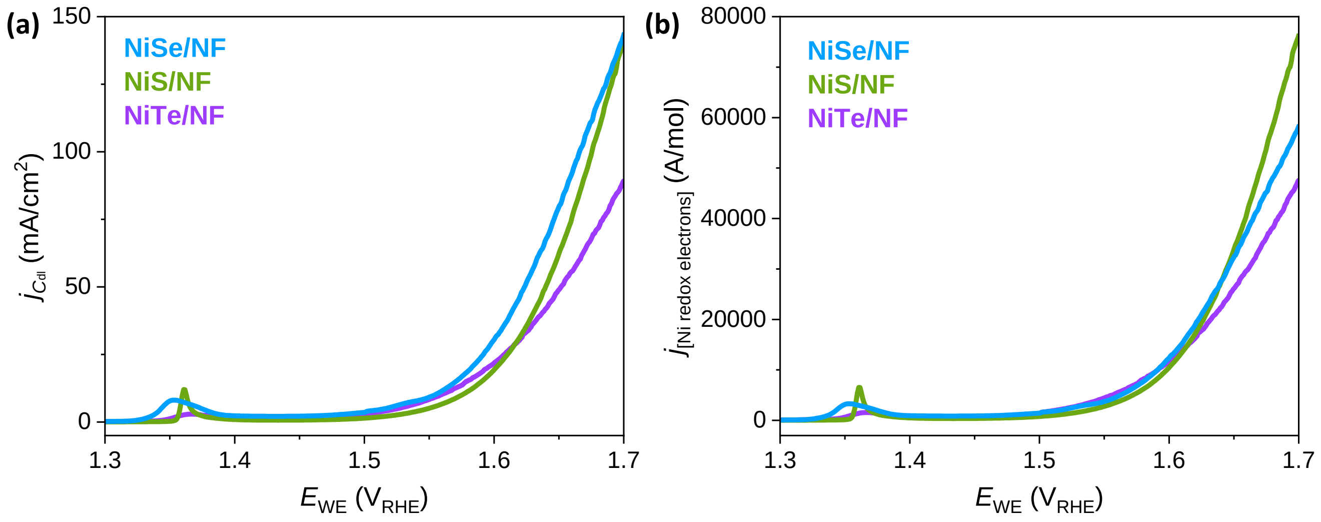

Figure S55. Steady-state OER activity of NiE/NF normalized by the (a) C_{dl} values and the (b) amount of redox active Ni electrons.

References

(1) Yao, S.; Driess, M. Lessons from Isolable Nickel(I) Precursor Complexes for Small Molecule Activation. *Acc. Chem. Res.* **2012**, *45* (2), 276–287. https://doi.org/10.1021/ar200156r.

(2) Alan Buckley, D. Preparation of Red Amorphous Selenium. *J. Chem. Soc. Abstr. Pap.* **1974**, *2* (9), 28–35.

(3) Sheldrick, G. M. SHELXT – Integrated Space-Group and Crystal-Structure Determination. *Acta Crystallogr. Sect. A Found. Adv.* **2015**, *71* (1), 3–8. https://doi.org/10.1107/S2053273314026370.

(4) Dolomanov, O. V.; Bourhis, L. J.; Gildea, R. J.; Howard, J. A. K.; Puschmann, H. OLEX2 : A Complete Structure Solution, Refinement and Analysis Program. *J. Appl. Crystallogr.* **2009**, *42* (2), 339–341. https://doi.org/10.1107/S0021889808042726.

(5) Hausmann, J. N.; Menezes, P. V.; Vijaykumar, G.; Laun, K.; Diemant, T.; Zebger, I.; Jacob, T.; Driess, M.; Menezes, P. W. In-Liquid Plasma Modified Nickel Foam: NiOOH/NiFeOOH Active Site Multiplication for Electrocatalytic Alcohol, Aldehyde, and Water Oxidation. *Adv. Energy Mater.* **2022**, *12* (38), 2202098. https://doi.org/10.1002/aenm.202202098.

(6) Yao, S.; Milsmann, C.; Bill, E.; Wieghardt, K.; Driess, M. From a Paramagnetic, Mononuclear Supersulfidonickel(II) Complex to a Diamagnetic Dimer with a Four-Sulfur Two-Electron Bond. *J. Am. Chem. Soc.* **2008**, *130* (41), 13536–13537. https://doi.org/10.1021/ja806101h.

(7) Yao, S.; Xiong, Y.; Zhang, X.; Schlangen, M.; Schwarz, H.; Milsmann, C.; Driess, M. Facile Dissociation of $[(LNi^{II})_2E_2]$ Dichalcogenides: Evidence for $[LNi^{II}E_2]$ Superselenides and Supertellurides in Solution. *Angew. Chem. Int. Ed.* **2009**, *48* (25), 4551–4554. https://doi.org/10.1002/anie.200901132.

(8) Rajamani, V.; Prewitt, C. T. The Crystal Structure of Millerite. *Canadian Mineralogist* **1974**, *12* (1), 253–257.

(9) Unoki, K.; Yoshiasa, A.; Kitahara, G.; Nishiayama, T.; Tokuda, M.; Sugiyama, K.; Nakatsuka, A. Crystal Structure Refinements of Stoichiometric Ni_3Se_2 and NiSe. *Acta Crystallogr. Sect. C Struct. Chem.* **2021**, *77* (4), 169–175. https://doi.org/10.1107/S2053229621002187.

(10) Chen, S.; Mi, J. L.; Zhang, P.; Feng, Y. H.; Yong, Y. C.; Shi, W. D. Control Synthesis of Nickel Selenides and Their Multiwalled Carbon Nanotubes Composites as Electrocatalysts for Enhanced Water Oxidation. *J. Phys. Chem. C* **2018**, *122* (45), 26096–26104. https://doi.org/10.1021/acs.jpcc.8b09259.

(11) Zhang, X.-Y.; Guo, B.-Y.; Chen, X.-Y.; Zhao, L.; Dong, B.; Yang, M.; Yu, J.-F.; Wang, L.; Liu, C.-G.; Chai, Y.-M. Template Confined Strategy for Constructing Nickel Cobalt Selenide Nanoarrays for Efficient Oxygen Evolution Reaction. *Mater. Today Energy* **2020**, *17*, 100468. https://doi.org/10.1016/j.mtener.2020.100468.

(12) Tian, L.; Wang, K.; Wo, H.; Pang, X.; Zhai, X.; Zhuang, W.; Li, T.; Chen, Y. Bundle-Shaped Cobalt–Nickel Selenides as Advanced Electrocatalysts for Water Oxidation. *Int. J. Hydrogen Energy* **2019**, *44* (5), 2868–2876. https://doi.org/10.1016/j.ijhydene.2018.12.074.

(13) Cai, C.; Mi, Y.; Han, S.; Wang, Q.; Liu, W.; Wu, X.; Zheng, Z.; Xia, X.; Qiao, L.; Zhou, W.; Zu, X. Engineering Ordered Dendrite-like Nickel Selenide as Electrocatalyst. *Electrochim. Acta* **2019**, *295*, 92–98. https://doi.org/10.1016/j.electacta.2018.10.083.

(14) Yang, H.; Huang, Y.; Teoh, W. Y.; Jiang, L.; Chen, W.; Zhang, L.; Yan, J. Molybdenum Selenide Nanosheets Surrounding Nickel Selenides Sub-Microislands on Nickel Foam as High-

Performance Bifunctional Electrocatalysts for Water Splitting. *Electrochim. Acta* **2020**, *349*, 136336. https://doi.org/10.1016/j.electacta.2020.136336.

(15) Li, X.; Zhang, L.; Huang, M.; Wang, S.; Li, X.; Zhu, H. Cobalt and Nickel Selenide Nanowalls Anchored on Graphene as Bifunctional Electrocatalysts for Overall Water Splitting. *J. Mater. Chem. A* **2016**, *4* (38), 14789–14795. https://doi.org/10.1039/C6TA07009D.

(16) Xu, X.; Song, F.; Hu, X. A Nickel Iron Diselenide-Derived Efficient Oxygen-Evolution Catalyst. *Nat. Commun.* **2016**, *7* (1), 12324. https://doi.org/10.1038/ncomms12324.

(17) Liu, P. F.; Zhang, L.; Zheng, L. R.; Yang, H. G. Surface Engineering of Nickel Selenide for an Enhanced Intrinsic Overall Water Splitting Ability. *Mater. Chem. Front.* **2018**, *2* (9), 1725–1731. https://doi.org/10.1039/C8QM00292D.

(18) Ghosh, S.; Samanta, M.; Das, B.; Maity, S.; Howli, P.; Sarkar, S.; Chattopadhyay, K. K. Hexagonal Nickel Selenide Nanoflakes Decorated Carbon Fabric: An Efficient Binder-Free Water Loving Electrode for Electrochemical Water Splitting. *Solid State Sci.* **2021**, *116*, 106613. https://doi.org/10.1016/j.solidstatesciences.2021.106613.

(19) Pan, Q.-R.; Li, S.-J.; Tong, K.; Xie, C.; Peng, L.; Li, N.; Wang, D.-Y.; Su, H. Engineering Ni^{3+} inside Nickel Selenide as Efficient Bifunctional Oxygen Electrocatalysts for Zn–Air Batteries. *J. Mater. Sci.* **2019**, *54* (12), 9063–9074. https://doi.org/10.1007/s10853-019-03520-w.

(20) Du, Y.; Cheng, G.; Luo, W. $NiSe_2$/$FeSe_2$ Nanodendrites: A Highly Efficient Electrocatalyst for Oxygen Evolution Reaction. *Catal. Sci. Technol.* **2017**, *7* (20), 4604–4608. https://doi.org/10.1039/C7CY01496A.

(21) Esmailzadeh, S.; Shahrabi, T.; Yaghoubinezhad, Y.; Barati Darband, G. Optimization of Nickel Selenide for Hydrogen and Oxygen Evolution Reactions by Response Surface Methodology. *J. Colloid Interface Sci.* **2021**, *600*, 324–337. https://doi.org/10.1016/j.jcis.2021.05.003.

(22) Wan, K.; Luo, J.; Zhang, X.; Subramanian, P.; Fransaer, J. Sulfur-Modified Nickel Selenide as an Efficient Electrocatalyst for the Oxygen Evolution Reaction. *J. Energy Chem.* **2021**, *62*, 198–203. https://doi.org/10.1016/j.jechem.2021.03.013.

(23) Feng, Z.; Zhang, H.; Wang, L.; Gao, B.; Lu, P.; Xing, P. Nanoporous Nickel-Selenide as High-Active Bifunctional Electrocatalyst for Oxygen Evolution and Hydrazine Oxidation. *J. Electroanal. Chem.* **2020**, *876*, 114740. https://doi.org/10.1016/j.jelechem.2020.114740.

(24) Kwak, I. H.; Im, H. S.; Jang, D. M.; Kim, Y. W.; Park, K.; Lim, Y. R.; Cha, E. H.; Park, J. $CoSe_2$ and $NiSe_2$ Nanocrystals as Superior Bifunctional Catalysts for Electrochemical and Photoelectrochemical Water Splitting. *ACS Appl. Mater. Interfaces* **2016**, *8* (8), 5327–5334. https://doi.org/10.1021/acsami.5b12093.

(25) Chaudhari, N. K.; Oh, A.; Sa, Y. J.; Jin, H.; Baik, H.; Kim, S. G.; Lee, S. J.; Joo, S. H.; Lee, K. Morphology Controlled Synthesis of 2-D Ni–Ni_3S_2 and Ni_3S_2 Nanostructures on Ni Foam towards Oxygen Evolution Reaction. *Nano Converg.* **2017**, *4* (1), 7. https://doi.org/10.1186/s40580-017-0101-6.

(26) Luo, P.; Zhang, H.; Liu, L.; Zhang, Y.; Deng, J.; Xu, C.; Hu, N.; Wang, Y. Targeted Synthesis of Unique Nickel Sulfide (NiS, NiS_2) Microarchitectures and the Applications for the Enhanced Water Splitting System. *ACS Appl. Mater. Interfaces* **2017**, *9* (3), 2500–2508. https://doi.org/10.1021/acsami.6b13984.

(27) Chinnadurai, D.; Rajendiran, R.; Kandasamy, P. Bimetallic Copper Nickel Sulfide Electrocatalyst by One Step Chemical Bath Deposition for Efficient and Stable Overall Water Splitting Applications. *J. Colloid Interface Sci.* **2022**, *606*, 101–112.

https://doi.org/10.1016/j.jcis.2021.07.145.
(28) Li, B.-Q.; Zhang, S.-Y.; Tang, C.; Cui, X.; Zhang, Q. Anionic Regulated NiFe (Oxy)Sulfide Electrocatalysts for Water Oxidation. *Small* **2017**, *13* (25), 1700610. https://doi.org/10.1002/smll.201700610.
(29) Ding, J.; Ji, S.; Wang, H.; Gai, H.; Liu, F.; Linkov, V.; Wang, R. Mesoporous Nickel-Sulfide/Nickel/N-Doped Carbon as HER and OER Bifunctional Electrocatalyst for Water Electrolysis. *Int. J. Hydrogen Energy* **2019**, *44* (5), 2832–2840. https://doi.org/10.1016/j.ijhydene.2018.12.031.
(30) Han, C.; Li, W.; Shu, C.; Guo, H.; Liu, H.; Dou, S.; Wang, J. Catalytic Activity Boosting of Nickel Sulfide toward Oxygen Evolution Reaction via Confined Overdoping Engineering. *ACS Appl. Energy Mater.* **2019**, *2* (8), 5363–5372. https://doi.org/10.1021/acsaem.9b00932.
(31) Ghosh, S.; Dasgupta, B.; Kalra, S.; Ashton, M. L. P.; Yang, R.; Kueppers, C. J.; Gok, S.; Alonso, E. G.; Schmidt, J.; Laun, K.; Zebger, I.; Walter, C.; Driess, M.; Menezes, P. W. Evolution of Carbonate-Intercalated γ-NiOOH from a Molecularly Derived Nickel Sulfide (Pre)Catalyst for Efficient Water and Selective Organic Oxidation. *Small* **2023**, *19* (16), 2206679. https://doi.org/10.1002/smll.202206679.
(32) He, W.; Ren, G.; Li, Y.; Jia, D.; Li, S.; Cheng, J.; Liu, C.; Hao, Q.; Zhang, J.; Liu, H. Amorphous Nickel–Iron Hydroxide Films on Nickel Sulfide Nanoparticles for the Oxygen Evolution Reaction. *Catal. Sci. Technol.* **2020**, *10* (6), 1708–1713. https://doi.org/10.1039/C9CY02345C.
(33) Bhat, K. S.; Barshilia, H. C.; Nagaraja, H. S. Porous Nickel Telluride Nanostructures as Bifunctional Electrocatalyst towards Hydrogen and Oxygen Evolution Reaction. *Int. J. Hydrogen Energy* **2017**, *42* (39), 24645–24655. https://doi.org/10.1016/j.ijhydene.2017.08.098.
(34) Qi, Y.; Yang, Z.; Peng, S.; Wang, M.; Bai, J.; Li, H.; Xiong, D. Self-Supported Cobalt–Nickel Bimetallic Telluride as an Advanced Catalyst for the Oxygen Evolution Reaction. *Inorg. Chem. Front.* **2021**, *8* (18), 4247–4256. https://doi.org/10.1039/D1QI00693B.
(35) Wang, Z.; Zhang, L. Nickel Ditelluride Nanosheet Arrays: A Highly Efficient Electrocatalyst for the Oxygen Evolution Reaction. *ChemElectroChem* **2018**, *5* (8), 1153–1158. https://doi.org/10.1002/celc.201701357.
(36) Görlin, M.; Ferreira de Araújo, J.; Schmies, H.; Bernsmeier, D.; Dresp, S.; Gliech, M.; Jusys, Z.; Chernev, P.; Kraehnert, R.; Dau, H.; Strasser, P. Tracking Catalyst Redox States and Reaction Dynamics in Ni–Fe Oxyhydroxide Oxygen Evolution Reaction Electrocatalysts: The Role of Catalyst Support and Electrolyte PH. *J. Am. Chem. Soc.* **2017**, *139* (5), 2070–2082. https://doi.org/10.1021/jacs.6b12250.
(37) Görlin, M.; Chernev, P.; Paciok, P.; Tai, C.-W.; Ferreira de Araújo, J.; Reier, T.; Heggen, M.; Dunin-Borkowski, R.; Strasser, P.; Dau, H. Formation of Unexpectedly Active Ni–Fe Oxygen Evolution Electrocatalysts by Physically Mixing Ni and Fe Oxyhydroxides. *Chem. Commun.* **2019**, *55* (6), 818–821. https://doi.org/10.1039/C8CC06410E.
(38) Li, N.; Bediako, D. K.; Hadt, R. G.; Hayes, D.; Kempa, T. J.; von Cube, F.; Bell, D. C.; Chen, L. X.; Nocera, D. G. Influence of Iron Doping on Tetravalent Nickel Content in Catalytic Oxygen Evolving Films. *Proc. Natl. Acad. Sci.* **2017**, *114* (7), 1486–1491. https://doi.org/10.1073/pnas.1620787114.
(39) Loos, S.; Zaharieva, I.; Chernev, P.; Lißner, A.; Dau, H. Electromodified NiFe Alloys as Electrocatalysts for Water Oxidation: Mechanistic Implications of Time-Resolved UV/Vis Tracking of Oxidation State Changes. *ChemSusChem* **2019**, *12* (9), 1966–1976.

https://doi.org/10.1002/cssc.201802737.
(40) Rahman, G.; Chae, S. Y.; Joo, O. Efficient Hydrogen Evolution Performance of Phase-Pure NiS Electrocatalysts Grown on Fluorine-Doped Tin Oxide-Coated Glass by Facile Chemical Bath Deposition. *Int. J. Hydrogen Energy* **2018**, *43* (29), 13022–13031. https://doi.org/10.1016/j.ijhydene.2018.05.049.
(41) Park, G. D.; Cho, J. S.; Kang, Y. C. Sodium-Ion Storage Properties of Nickel Sulfide Hollow Nanospheres/Reduced Graphene Oxide Composite Powders Prepared by a Spray Drying Process and the Nanoscale Kirkendall Effect. *Nanoscale* **2015**, *7* (40), 16781–16788. https://doi.org/10.1039/C5NR04252F.
(42) Kim, M.; Park, G. D.; Kang, Y. C. Investigation of the Potassium-ion Storage Mechanism of Nickel Selenide Materials and Rational Design of Nickel selenide-C Yolk-shell Structure for Enhancing Electrochemical Properties. *Int. J. Energy Res.* **2022**, *46* (5), 5800–5810. https://doi.org/10.1002/er.7523.
(43) Weidler, N.; Schuch, J.; Knaus, F.; Stenner, P.; Hoch, S.; Maljusch, A.; Schäfer, R.; Kaiser, B.; Jaegermann, W. X-Ray Photoelectron Spectroscopic Investigation of Plasma-Enhanced Chemical Vapor Deposited NiO_x, $NiO_x(OH)_y$, and $CoNiO_x(OH)_y$: Influence of the Chemical Composition on the Catalytic Activity for the Oxygen Evolution Reaction. *J. Phys. Chem. C* **2017**, *121* (12), 6455–6463. https://doi.org/10.1021/acs.jpcc.6b12652.
(44) Ratcliff, E. L.; Meyer, J.; Steirer, K. X.; Garcia, A.; Berry, J. J.; Ginley, D. S.; Olson, D. C.; Kahn, A.; Armstrong, N. R. Evidence for Near-Surface NiOOH Species in Solution-Processed NiO_x Selective Interlayer Materials: Impact on Energetics and the Performance of Polymer Bulk Heterojunction Photovoltaics. *Chem. Mater.* **2011**, *23* (22), 4988–5000. https://doi.org/10.1021/cm202296p.
(45) Ahmad, M.; Agarwal, K.; Mehta, B. R. An Anomalously High Seebeck Coefficient and Power Factor in Ultrathin Bi_2Te_3 Film: Spin–Orbit Interaction. *J. Appl. Phys.* **2020**, *128* (3). https://doi.org/10.1063/5.0007440.
(46) Reese, M. O.; Perkins, C. L.; Burst, J. M.; Farrell, S.; Barnes, T. M.; Johnston, S. W.; Kuciauskas, D.; Gessert, T. A.; Metzger, W. K. Intrinsic Surface Passivation of CdTe. *J. Appl. Phys.* **2015**, *118* (15), 155305. https://doi.org/10.1063/1.4933186.
(47) Trześniewski, B. J.; Diaz-Morales, O.; Vermaas, D. A.; Longo, A.; Bras, W.; Koper, M. T. M.; Smith, W. A. In Situ Observation of Active Oxygen Species in Fe-Containing Ni-Based Oxygen Evolution Catalysts: The Effect of pH on Electrochemical Activity. *J. Am. Chem. Soc.* **2015**, *137* (48), 15112–15121. https://doi.org/10.1021/jacs.5b06814.
(48) Menezes, P. W.; Yao, S.; Beltrán-Suito, R.; Hausmann, J. N.; Menezes, P. V.; Driess, M. Facile Access to an Active γ-NiOOH Electrocatalyst for Durable Water Oxidation Derived From an Intermetallic Nickel Germanide Precursor. *Angew. Chem. Int. Ed.* **2021**, *60* (9), 4640–4647. https://doi.org/10.1002/anie.202014331.
(49) Chen, Z.; Yang, H.; Mebs, S.; Dau, H.; Driess, M.; Wang, Z.; Kang, Z.; Menezes, P. W. Reviving Oxygen Evolution Electrocatalysis of Bulk La–Ni Intermetallics via Gaseous Hydrogen Engineering. *Adv. Mater.* **2023**, *35* (11), 2208337. https://doi.org/10.1002/adma.202208337.
(50) Mondal, I.; Hausmann, J. N.; Vijaykumar, G.; Mebs, S.; Dau, H.; Driess, M.; Menezes, P. W. Nanostructured Intermetallic Nickel Silicide (Pre)Catalyst for Anodic Oxygen Evolution Reaction and Selective Dehydrogenation of Primary Amines. *Adv. Energy Mater.* **2022**, *12* (25),

2200269. https://doi.org/10.1002/aenm.202200269.

(51) Gouda, L.; Sévery, L.; Moehl, T.; Mas-Marzá, E.; Adams, P.; Fabregat-Santiago, F.; Tilley, S. D. Tuning the Selectivity of Biomass Oxidation over Oxygen Evolution on NiO–OH Electrodes. *Green Chem.* **2021**, *23* (20), 8061–8068. https://doi.org/10.1039/D1GC02031E.

(52) Li, R.; Kuang, P.; Wang, L.; Tang, H.; Yu, J. Engineering 2D NiO/Ni_3S_2 Heterointerface Electrocatalyst for Highly Efficient Hydrogen Production Coupled with Benzyl Alcohol Oxidation. *Chem. Eng. J.* **2022**, *431* (P2), 134137. https://doi.org/10.1016/j.cej.2021.134137.

(53) Liu, G.; Zhang, X.; Zhao, C.; Xiong, Q.; Gong, W.; Wang, G.; Zhang, Y.; Zhang, H.; Zhao, H. Electrocatalytic Oxidation of Benzyl Alcohol for Simultaneously Promoting H_2 Evolution by a $Co_{0.83}Ni_{0.17}$/Activated Carbon Electrocatalyst. *New J. Chem.* **2018**, *42* (8), 6381–6388. https://doi.org/10.1039/C8NJ00446C.

(54) Xu, M.; Geng, J.; Xu, H.; Zhang, S.; Zhang, H. In Situ Construction of $NiCo_2O_4$ Nanosheets on Nickel Foam for Efficient Electrocatalytic Oxidation of Benzyl Alcohol. *Inorg. Chem. Front.* **2023**, *10* (7), 2053–2059. https://doi.org/10.1039/D2QI02526D.

(55) Ming, L.; Wu, X.-Y.; Wang, S.-S.; Wu, W.; Lu, C.-Z. Facile Growth of Transition Metal Hydroxide Nanosheets on Porous Nickel Foam for Efficient Electrooxidation of Benzyl Alcohol. *Green Chem.* **2021**, *23* (19), 7825–7830. https://doi.org/10.1039/D1GC02218K.

(56) You, B.; Liu, X.; Liu, X.; Sun, Y. Efficient H 2 Evolution Coupled with Oxidative Refining of Alcohols via A Hierarchically Porous Nickel Bifunctional Electrocatalyst. *ACS Catal.* **2017**, *7* (7), 4564–4570. https://doi.org/10.1021/acscatal.7b00876.

(57) Hu, S.; Sun, X.; Liu, Z.; Gao, L.; Li, X.; Yu, C.; Han, X.; Xie, J.; Sun, X. Annealing Activated Nickel–Molybdenum Oxide as an Efficient Electrocatalyst toward Benzyl Alcohol Upgrading. *Energy Adv.* **2024**, *3* (1), 281–286. https://doi.org/10.1039/D3YA00447C.

(58) Li, F.; Liu, C.; Lin, H.; Sun, Y.; Yu, H.; Xue, S.; Cao, J.; Jia, X.; Chen, S. High Activity of Bifunctional Ni2P Electrocatalyst for Benzyl Alcohol Oxidation Coupled with Hydrogen Evolution. *J. Colloid Interface Sci.* **2023**, *640*, 329–337. https://doi.org/10.1016/j.jcis.2023.02.121.

(59) Chen, X.; Zhong, X.; Yuan, B.; Li, S.; Gu, Y.; Zhang, Q.; Zhuang, G.; Li, X.; Deng, S.; Wang, J. Defect Engineering of Nickel Hydroxide Nanosheets by Ostwald Ripening for Enhanced Selective Electrocatalytic Alcohol Oxidation. *Green Chem.* **2019**, *21* (3), 578–588. https://doi.org/10.1039/C8GC03451F.

(60) Ghosh, S.; Hausmann, J. N.; Reith, L.; Vijaykumar, G.; Schmidt, J.; Laun, K.; Berendts, S.; Zebger, I.; Driess, M.; Menezes, P. W. Nitridated Nickel Mesh as Industrial Water and Alcohol Oxidation Catalyst: Reconstruction and Iron-Incorporation Matters. *Adv. Energy Mater.* **2024**, *14* (22), 2400356. https://doi.org/10.1002/aenm.202400356.

(61) Wan, J.; Mu, X.; Jin, Y.; Zhu, J.; Xiong, Y.; Li, T.; Li, R. Nitrogen-Doped Nickel-Molybdenum Oxide as a Highly Efficient Electrocatalyst for Benzyl Alcohol Oxidation. *Green Chem.* **2022**, *24* (12), 4870–4876. https://doi.org/10.1039/D2GC00989G.

(62) Zhong, J.; Shen, Y.; Zhu, P.; Yao, S.; An, C. Size-Effect on Ni Electrocatalyst: The Case of Electrochemical Benzyl Alcohol Oxidation. *Nano Res.* **2023**, *16* (1), 202–208. https://doi.org/10.1007/s12274-022-4679-6.

(63) Li, J.; Zhang, J.; Gao, W.; Cheng, X.; Zhao, X.; Gao, S.; Shang, N.; Wang, C. Synergistically Coupling of Ultrathin Ni_3N Layer with $Ti_3C_2T_x$-MXene Nanosheets for Efficient Benzyl Alcohol Oxidation Reactions and Hydrogen Production. *Chem. Eng. Sci.* **2024**, *288*, 119813.

https://doi.org/10.1016/j.ces.2024.119813.

(64) Du, X.; Xie, W.; Wang, Y.; Li, H.; Li, J.; Li, Y.; Song, Y.; Li, Z.; Lee, J. Y.; Shao, M. Steering Benzyl Alcohol Electrooxidation Coupled with Hydrogen Evolution via Hetero-interface Construction. *AIChE J.* **2024**, *70* (8), 1–12. https://doi.org/10.1002/aic.18469.

(65) Huang, H. H.; Yu, C.; Han, X.; Huang, H. H.; Wei, Q.; Guo, W.; Wang, Z.; Qiu, J. Ni, Co Hydroxide Triggers Electrocatalytic Production of High-Purity Benzoic Acid over 400 mA cm^{-2}. *Energy Environ. Sci.* **2020**, *13* (12), 4990–4999. https://doi.org/10.1039/d0ee02607g.

(66) Li, J.-K.; Wang, A.; Dong, X.-Y.; Huang, S.; Meng, Y.; Song, J.-L. Construction of 2D C,N-Co-Doped ZnO/Co_3O_4 over $Ni(OH)_2$ Mesoporous Ultrathin Nanosheets on Ni Foam as High-Performance Electrocatalysts for Benzyl-Alcohol Oxidation and Accelerating Hydrogen Evolution. *New J. Chem.* **2023**, *47* (12), 5970–5976. https://doi.org/10.1039/D3NJ00236E.

(67) Zhang, N.; Zou, Y.; Tao, L.; Chen, W.; Zhou, L.; Liu, Z.; Zhou, B.; Huang, G.; Lin, H.; Wang, S. Electrochemical Oxidation of 5-Hydroxymethylfurfural on Nickel Nitride/Carbon Nanosheets: Reaction Pathway Determined by In Situ Sum Frequency Generation Vibrational Spectroscopy. *Angew. Chem. Int. Ed.* **2019**, *58* (44), 15895–15903. https://doi.org/10.1002/anie.201908722.

(68) Zhang, J.; Yu, P.; Zeng, G.; Bao, F.; Yuan, Y.; Huang, H. Boosting HMF Oxidation Performance via Decorating Ultrathin Nickel Hydroxide Nanosheets with Amorphous Copper Hydroxide Islands. *J. Mater. Chem. A* **2021**, *9* (15), 9685–9691. https://doi.org/10.1039/D0TA11678E.

(69) Zhang, B.; Fu, H.; Mu, T. Hierarchical NiS_x/Ni_2P Nanotube Arrays with Abundant Interfaces for Efficient Electrocatalytic Oxidation of 5-Hydroxymethylfurfural. *Green Chem.* **2022**, *24* (2), 877–884. https://doi.org/10.1039/D1GC04206H.

(70) Barwe, S.; Weidner, J.; Cychy, S.; Morales, D. M.; Dieckhöfer, S.; Hiltrop, D.; Masa, J.; Muhler, M.; Schuhmann, W. Electrocatalytic Oxidation of 5-(Hydroxymethyl)Furfural Using High-Surface-Area Nickel Boride. *Angew. Chem. Int. Ed.* **2018**, *57* (35), 11460–11464. https://doi.org/10.1002/anie.201806298.

(71) Xing, M.; Zhang, D.; Liu, D.; Song, C.; Wang, D. Surface Engineering of Carbon-Coated Cobalt-Doped Nickel Phosphides Bifunctional Electrocatalyst for Boosting 5-Hydroxymethylfurfural Oxidation Coupled with Hydrogen Evolution. *J. Colloid Interface Sci.* **2023**, *629*, 451–460. https://doi.org/10.1016/j.jcis.2022.09.091.

(72) Kong, F.; Wang, M. Preparation of Sulfur-Modulated Nickel/Carbon Composites from Lignosulfonate for the Electrocatalytic Oxidation of 5-Hydroxymethylfurfural to 2,5-Furandicarboxylic Acid. *ACS Appl. Energy Mater.* **2021**, *4* (2), 1182–1188. https://doi.org/10.1021/acsaem.0c02418.

(73) Dasgupta, B.; Hausmann, J. N.; Beltrán-Suito, R.; Kalra, S.; Laun, K.; Zebger, I.; Driess, M.; Menezes, P. W. A Facile Molecular Approach to Amorphous Nickel Pnictides and Their Reconstruction to Crystalline Potassium-Intercalated γ-$NiOOH_x$ Enabling High-Performance Electrocatalytic Water Oxidation and Selective Oxidation of 5-Hydroxymethylfurfural. *Small* **2023**, *19* (33), 2301258. https://doi.org/10.1002/smll.202301258.

(74) Xu, P.; Bao, Z.; Zhao, Y.; Zheng, L.; Lv, Z.; Shi, X.; Wang, H.; Fang, X.; Zheng, H. Anionic Regulation and Heteroatom Doping of Ni-Based Electrocatalysts to Boost Biomass Valorization Coupled with Hydrogen Production. *Adv. Energy Mater.* **2024**, *14* (2), 1–11. https://doi.org/10.1002/aenm.202303557.

(75) Zhang, H.; Yang, Q.; Luo, S.; Liu, Z.; Huang, J.; Zheng, Y.; Hu, C.; Zhang, J.; Bao, X.; Yuan, P.; Yao, X. On the Activity and Selectivity of 5-Hydroxymethylfurfural Electrocatalytic Oxidation over Cation-Defective Nickel Hydroxides. *ACS Catal.* **2024**, *14* (12), 9565–9574. https://doi.org/10.1021/acscatal.4c01407.

(76) Zheng, L.; Zhao, Y.; Xu, P.; Lv, Z.; Shi, X.; Zheng, H. Biomass Upgrading Coupled with H_2 Production via a Nonprecious and Versatile Cu-Doped Nickel Nanotube Electrocatalyst. *J. Mater. Chem. A* **2022**, *10* (18), 10181–10191. https://doi.org/10.1039/D2TA00579D.

(77) Kahlstorf, T.; Niklas Hausmann, J.; Mondal, I.; Laun, K.; Zebger, I.; Sontheimer, T.; Menezes, P. W. Water-Soluble Nickel and Iron Salts for Hydroxymethylfurfural (HMF) and Water Oxidation: The Simplest Precatalysts? *Green Chem.* **2023**, *25* (21), 8679–8686. https://doi.org/10.1039/D3GC02119J.

(78) Yang, C.; Wang, C.; Zhou, L.; Duan, W.; Song, Y.; Zhang, F.; Zhen, Y.; Zhang, J.; Bao, W.; Lu, Y.; Wang, D.; Fu, F. Refining D-Band Center in $Ni_{0.85}Se$ by Mo Doping: A Strategy for Boosting Hydrogen Generation via Coupling Electrocatalytic Oxidation 5-Hydroxymethylfurfural. *Chem. Eng. J.* **2021**, *422* (February), 130125. https://doi.org/10.1016/j.cej.2021.130125.

(79) Chen, D.; Ding, Y.; Cao, X.; Wang, L.; Lee, H.; Lin, G.; Li, W.; Ding, G.; Sun, L. Highly Efficient Biomass Upgrading by a Ni−Cu Electrocatalyst Featuring Passivation of Water Oxidation Activity. *Angew. Chem. Int. Ed.* **2023**, *62* (37). https://doi.org/10.1002/anie.202309478.

(80) Zhang, J.; Gong, W.; Yin, H.; Wang, D.; Zhang, Y.; Zhang, H.; Wang, G.; Zhao, H. In Situ Growth of Ultrathin $Ni(OH)_2$ Nanosheets as Catalyst for Electrocatalytic Oxidation Reactions. *ChemSusChem* **2021**, *14* (14), 2935–2942. https://doi.org/10.1002/cssc.202100811.

(81) Wang, W.; Kong, F.; Zhang, Z.; Yang, L.; Wang, M. Sulfidation of Nickel Foam with Enhanced Electrocatalytic Oxidation of 5-Hydroxymethylfurfural to 2,5-Furandicarboxylic Acid. *Dalt. Trans.* **2021**, *50* (31), 10922–10927. https://doi.org/10.1039/D1DT02025K.

(82) Cheng, Z.; Deng, W.; Chen, R.; Tan, L.; Xie, C.; Ma, M.; Tan, Y. Promoted Formation of Catalytically Active Species in Ni Nanoparticles by Low Content Ru Doping for Electrocatalytic Oxidation of 5-Hydroxymethylfurfural. *ACS Sustain. Chem. Eng.* **2023**, *11* (36), 13441–13450. https://doi.org/10.1021/acssuschemeng.3c03489.

(83) Li, S.; Wang, S.; Wang, Y.; He, J.; Li, K.; Xu, Y.; Wang, M.; Zhao, S.; Li, X.; Zhong, X.; Wang, J. Doped Mn Enhanced NiS Electrooxidation Performance of HMF into FDCA at Industrial-Level Current Density. *Adv. Funct. Mater.* **2023**, *33* (24), 1–10. https://doi.org/10.1002/adfm.202214488.

(84) Song, X.; Liu, X.; Wang, H.; Guo, Y.; Wang, Y. Improved Performance of Nickel Boride by Phosphorus Doping as an Efficient Electrocatalyst for the Oxidation of 5-Hydroxymethylfurfural to 2,5-Furandicarboxylic Acid. *Ind. Eng. Chem. Res.* **2020**, *59* (39), 17348–17356. https://doi.org/10.1021/acs.iecr.0c01312.

(85) Xiao, H.; Shin, H.; Goddard, W. A. Synergy between Fe and Ni in the Optimal Performance of (Ni,Fe)OOH Catalysts for the Oxygen Evolution Reaction. *Proc. Natl. Acad. Sci. U. S. A.* **2018**, *115* (23), 5872–5877. https://doi.org/10.1073/pnas.1722034115.

(86) Moysiadou, A.; Lee, S.; Hsu, C.-S.; Chen, H. M.; Hu, X. Mechanism of Oxygen Evolution Catalyzed by Cobalt Oxyhydroxide: Cobalt Superoxide Species as a Key Intermediate and Dioxygen Release as a Rate-Determining Step. *J. Am. Chem. Soc.* **2020**, *142* (27), 11901–

11914. https://doi.org/10.1021/jacs.0c04867.

(87) Ghosh, S.; Bagchi, D.; Mondal, I.; Sontheimer, T.; Jagadeesh, R. V.; Menezes, P. W. Deciphering the Role of Nickel in Electrochemical Organic Oxidation Reactions. *Adv. Energy Mater.* **2024**, *14* (22), 2400696. https://doi.org/10.1002/aenm.202400696.
